# Stress response in the daily lives of simulation repeaters. A randomized controlled trial assessing stress evolution over one year of repetitive immersive simulations

**DOI:** 10.1371/journal.pone.0220111

**Published:** 2019-07-25

**Authors:** Daniel Aiham Ghazali, Cyril Breque, Philippe Sosner, Mathieu Lesbordes, Jean-Jacques Chavagnat, Stéphanie Ragot, Denis Oriot

**Affiliations:** 1 Emergency Department and Emergency Medical Service, University Hospital of Bichat, Paris, France; 2 Ilumens, Simulation Center, University of Paris-Diderot, Paris, France; 3 ABS Lab, Simulation Center, Faculty of Medicine, University of Poitiers, Poitiers, France; 4 Diagnosis and Therapeutic Center, University Hospital Hôtel-Dieu, Paris, France; 5 Cardiology Department, University Hospital of Poitiers, Poitiers, France; 6 Adult Psychiatric Unit, Hospital of Poitiers, Poitiers, France; 7 Statistical Department and Clinical Investigation Center (CIC 1402), INSERM (French National Health and Medical Research Institute), University Hospital of Poitiers, Poitiers, France; 8 Pediatric Emergency Department, University Hospital of Poitiers, Poitiers, France; Public Library of Science, UNITED KINGDOM

## Abstract

**Background:**

Simulations in healthcare reproduce clinical situations in stressful conditions. Repeated stress exposure might influence the learning process in simulation as well as real-life.

**Objectives:**

1) To record heart rate and heart rate variability evolution during one-day simulation over one year; 2) To analyze the effect of repetitive high-fidelity simulations on the risk of post-traumatic stress disorder.

**Study design:**

Single-center, investigator-initiated RCT. 48 participants were randomized in 12 multidisciplinary teams of French Emergency Medical Services to manage infant shock in high-fidelity simulations. In the experimental group, 6 multidisciplinary teams were exposed to 9 different simulation sessions over 1 year. In the control group, 6 multidisciplinary teams participated in only 3 simulation sessions, in common with those of the experimental group (initial, intermediate after 6 months, and finally after 1 year). Heart rate (HR) and heart rate variability (HRV) were analyzed on a 24-hour Holter from the day prior to simulation until the end of simulation. Questionnaires of Impact of Event Scale-Revised at 7 days and Post-traumatic Check-List Scale at 1 month were used to detect possible post-traumatic stress disorder in participants. p<0.05 was considered significant.

**Results:**

Stress increased during each simulation in the two groups. After analysis on the 24-hour period, there was no significant difference between the two groups during the initial simulation session in terms of heart rate and heart rate variability. In the 24-hour period of the intermediate and final simulation sessions, the level of stress was higher in the control group during the diurnal (p = 0.04) and nocturnal periods (p = 0.01). No participant developed post-traumatic stress disorder after the 72 simulation sessions.

**Conclusions:**

Despite the stress generated by simulation, the more the sessions were repeated, the less were their repercussions on the daily lives of participants, reflected by a lower sympathetic activity. Moreover, repetition of simulations did not lead to post-traumatic stress disorder.

**Trial registration:**

ClinicalTrials.gov NCT02424890.

## Introduction

Management of life-threatening events is a challenge in Emergency Medicine. Human error and system failures substantially contribute to adverse outcomes in health care [1]. Simulation helps to improve team performance and to reduce human errors [2] with an appreciable impact on patient safety [3]. Use of high-fidelity simulation (HFS) in a realistic environment improves management in critical conditions due to multidisciplinary team (MDT) training [4]. Simulation-based education is a method used in adult [5] and paediatric [6] emergency departments to teach technical and non-technical skills [7]. Moreover, stress occurring during emergency management of patients requires consideration, since it can compromise their safety [8]. Emergency teams face unexpected and unpredictable situations requiring prompt decision-making in stressful conditions [9]. Consequently, these HFSs tend to reproduce clinical situations in a realistic environment with sources of stress. Many studies have assessed stress during emergency HFS based on the physiological parameters of stress [10,11] or on perceived stress [12]. However, the relationship between self-reported and physiological stress is not as clear [13]. In a previous publication, we suggested that stress should be assessed using combined parameters of physiological and perceived stress [14]. Several authors have demonstrated that stress increases during HFS [15,16]. Moreover, simulator and electronic devices also provide stress [17]. Simulation itself and its environmental conditions generate stress that *in fine* can impact participants’ performances. Usually, stress is studied only during the phase of simulation, at times including debriefing [15]. We previously found that stress increased during simulation and decreased during and after debriefing [14,18]. Repeated simulations using salivary cortisol did not blunt it during the simulation phase [19]. However, to our knowledge, up until now no study has analyzed level of stress in participants during the whole day of planned simulation training and its evolution when simulations are repeated. It seems quite unlikely that the simulations in and of themselves would induce full-blown PTSD in emergency medicine team members, even though these experiences are known to be quite unpleasant and stressful. However, no study has analyzed the impact of repeated stress exposure in terms of post-traumatic stress disorder (PTSD). It is unknown whether such conditions might psychologically and physiologically impact participants. Repeated stress exposure might influence the learning process in simulation as well as real-life. Over 24 hours the heart rate variability (HRV), commonly recognized as a tool for assessment of the autonomic nervous system (ANS), of each participant was analyzed. HRV represents “sympathovagal balance” and is now considered as a more sensitive assessment of stress than heart rate (HR) activity [20]. Spectral domain (i.e. frequency analysis) of HRV corresponds to the fast Fournier transform algorithm of time domain of HRV (pNN50, proportion of interval differences of successive QRS complexes greater than 50ms). It is an indicator of sympathetic nervous system activity. Frequency analysis distinguishes the activities of sympathetic and parasympathetic contrary to temporal analysis that produces a “global” analysis of sympathovagal balance. HRV in spectral domain does not constantly represent dominance of sympathetic nervous system activity because the contributions of sympathetic activity to this metric substantially vary according to testing conditions. In any event, spectral analysis of HRV can be used during HFS to assess sympathetic nervous system activity related to increased stress level [14]. Stress might be expressed during the entire day of simulation and could have a long-time effect with a risk of PTSD if exposure is repeated. The aim of this prospective study was to analyze physiological stress of participants in two randomized groups according to the frequency of repetition of simulations. Stress response was analyzed over 24 hours, including before, during and after a simulation session, and to assess its evolution during repeated sessions over one year. Possible occurrence of PTSD was also searched over one year of stressful simulations.

The primary objective was to analyze sympathovagal balance by assessing in each participant the evolution of electrophysiological markers of stress during the 24-hour period. Measurements were also carried out before, during and after each simulation session in a program of repeated pediatric simulation-based training (SBT) over one year.

The secondary objectives were: 1) To look for a professional status effect, i.e., difference in stress response between physicians, residents, nurses, and ambulance drivers; 2) To look for a gender difference; 3) To study the effect of repetitive HFS on the risk of PTSD.

## Methods

### Study

The present study was a part of a single-center, investigator-initiated RCT including 12 months of simulation sessions [21]. It took place in the Simulation Laboratory, ABS Lab–INSERM (French national health and medical research institute) #1402, Faculty of Medicine, University of Poitiers, France. This RCT was registered by ClinicalTrials.gov under # NCT02424890. The study protocol, information form, and consent form were approved by the Comité de Protection des Personnes III de la region Ouest (Western France Person Protection Committee III) and registered under # 13.05.16 (S1 and S2 Files). The CONSORT Checklist is given in S1 Checklist.

### Population

**Fig 1:**
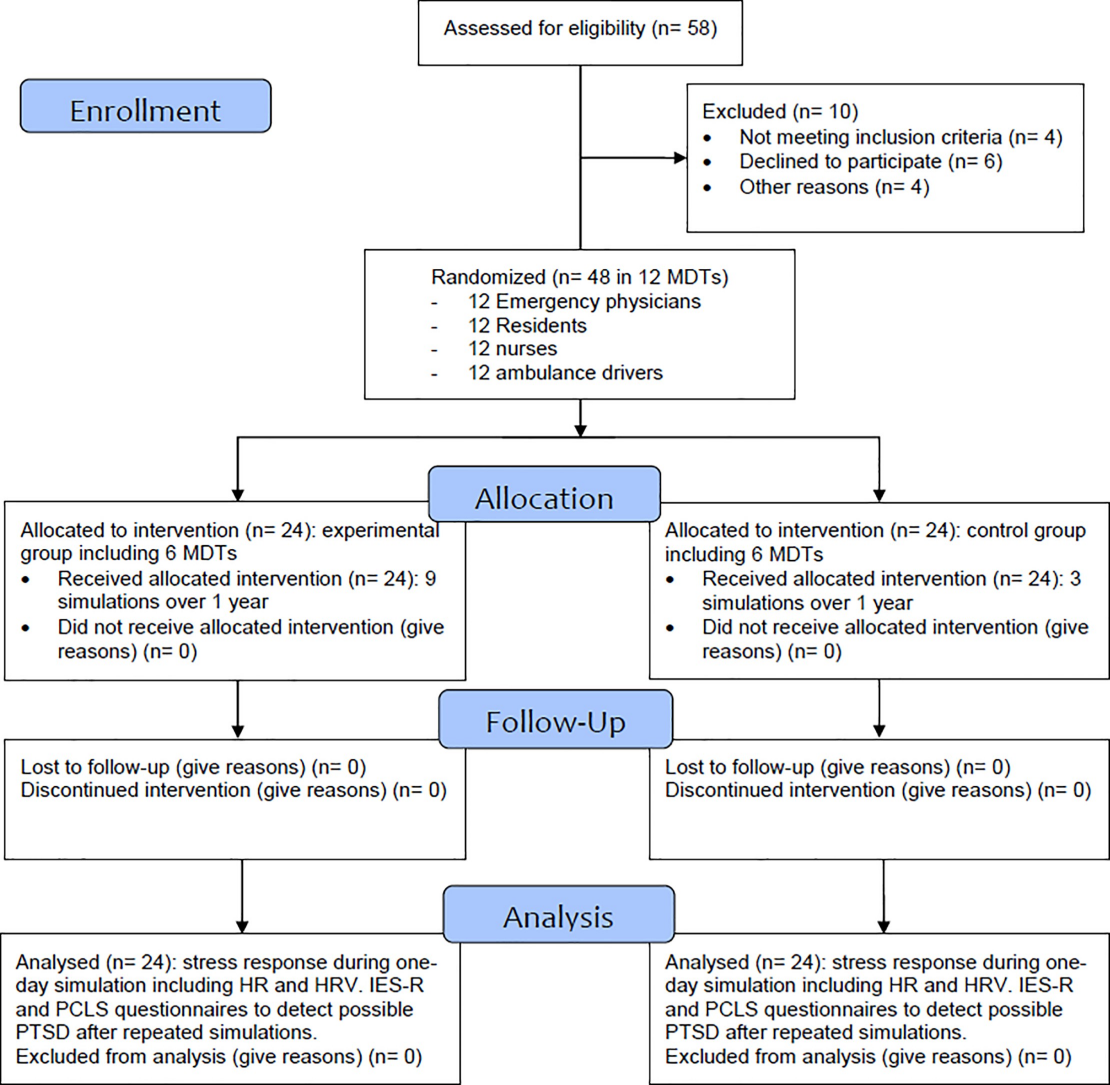
CONSORT 2010 flow diagram.

Forty-eight participants (Fig 1) were randomized in 12 MDTs of French Emergency Medical Services (EMS) to manage prehospital pediatric emergencies (including 4 members: physician, resident, nurse, and ambulance driver). Because of an estimated refusal rate of 50%, we randomly considered 24 persons of each status for involvement in the study. Participants were contacted by email for presentation of the study and consent to participate. In case of agreement, a final consent form was signed before the first session. Twelve participants for each status were drawn by lots by the trial coordinator among each status population (until all consented) and randomized to form different teams. Participants knew that they would be randomized in a team to carry out three or nine simulations over one year to manage infant shock in HFS. A second randomization was performed on the 12 MDTs by the methodologist to successively constitute teams from the first to the twelfth. The first six teams and the last six teams were allocated to the experimental and control groups respectively. Each participant had less than 7 years of professional experience in EMS. All participants had professional degrees and certifications considered as appropriate to their professional discipline according to the European Resuscitation Council guidelines [22]. No participant had a past history such as disease that could induce stress-related modifications, or worsen due to stress and/or psychiatric disease modifying stress response. Moreover, participants were undergoing no treatment like steroids or hormone replacement therapy that could interfere with stress markers. No participant had a night-shift the day prior to the simulation and participants were asked if they had been confronted with a stressful event the day prior to or the day of the simulation. Inclusion criteria, exclusion criteria, and the rest of the study protocol were previously published [21].

### Intervention

**Fig 2:**
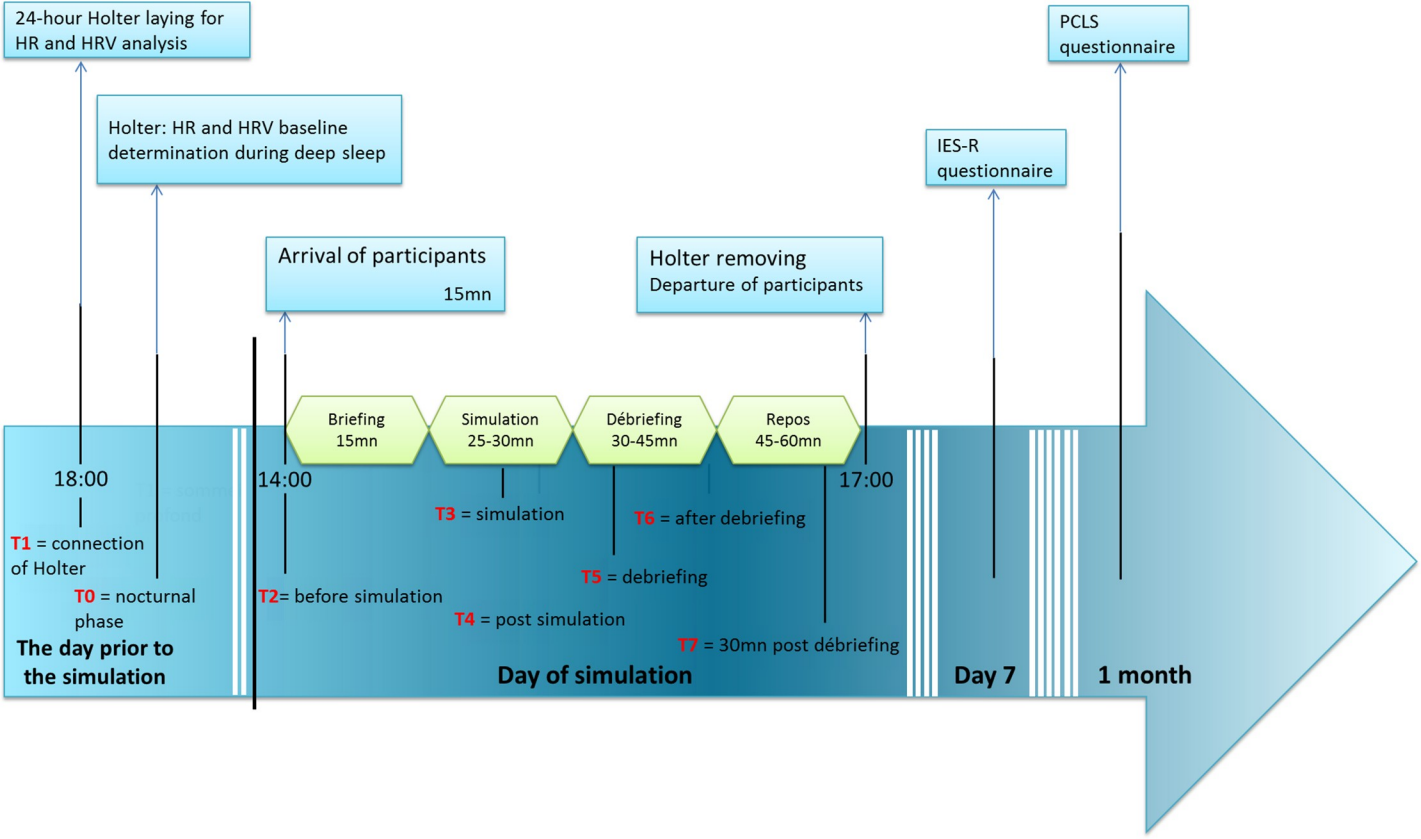
Study design. HR: heart rate; HR: heart rate variability; IES-R: Impact of Event Scale–Revised; PCLS: Post-traumatic Check-List Scale. T0 to T7: times of HR and HRV analysis in addition to the analysis over 24-hour period.

In the experimental group, 6 MDTs were exposed to 9 different simulation sessions over 1 year (every 6 weeks). In the control group, 6 MDTs had only 3 simulation sessions in common with those of the experimental group (initial, intermediate after 6 months and finally after 1 year). All in all, 72 simulations were carried out. The choice of number of simulation sessions for the experimental and control groups was designed to analyze performance evolution, one of the goals of the larger RCT [21]. Frequency of repetition would vary from monthly [23] to biannual repetition [24] to minimize a risk of declining performance [25]. Immersive simulation sessions used scenarios of infant shock requiring intra-osseous access on a high-fidelity manikin (SimNewB, Laerdal). Scenarios were preprogrammed to be identical for all teams in terms of objectives (technical and non-technical). If an appropriate action was not performed, the mannequin deteriorated but did not “die”. There is considerable controversy over whether to allow the manikin to die, since its “death” can influence stress and performance. Consequently, to avoid bias in comparison between two groups, and in analysis of stress evolution during repeated sessions, all simulations were carried out in similar conditions. They were scheduled on the same day of the week (Thursday) at 2:00 pm for the sake of comparison of stress parameters (Fig 2). Moreover, sessions were scheduled on participants' days off and not preceded by a night shift, thereby virtually guaranteeing a night of sleep for every participant before the day of simulation. HR and HRV baseline (T0) were determined during deep sleep. Each session included 15 min of briefing, 25–30 min of simulation, and a 30–45 min “good-judgment” debriefing [26]. Debriefing with good judgment uses the advocacy-inquiry technique as a conversational technique to discover gaps in participants’ performance related to cognitive and behavioral attributes [27]. Heart rate variability in spectral (i.e. frequency) domain was obtained with Synscope (Sorin Group) software during 24h of recording, starting on the day prior to simulation until the break after debriefing [14]. Potential PTSD was detected by questionnaires: Impact of Event Scale-Revised (IES-R) on the 7th day after the event [28] and Post-traumatic Check-List Scale (PCLS) at 1 month [29]. These questionnaires were related to a specific event to which PTSD symptoms might be keyed. In the present study, participants were informed that PCLS and IES-R referred specifically to the last simulation performed. Participants were asked to fill out the questionnaires. If they did not, they received an E-mail reminder after one and two and four days. If they did not answer to the questionnaires, IES-R and PCLS score were considered as missing data.

### Comparison

HR and HRV variation before, during and after simulation and over 24 hours were compared between the two randomized groups according to the frequency of repetition of simulations during the three common simulations (initial, intermediate, and final sessions). Holter laying was performed the day prior the simulation at 18:00 and removal took place on the day of the simulation at 17:00. Seven times were analyzed (Fig 2): T1: connection of Holter (day prior to the simulation at 18:00); T2: immediately before simulation (at 14:00); T3: during simulation; T4: after simulation; T5: during debriefing, T6: after debriefing, and T7: 30 min after debriefing. Analysis over 24 hours included diurnal and nocturnal phases. Individuals’ sleeping and waking times were assessed and used to determine diurnal and nocturnal phases. Stress response was analyzed according to professional status and gender.

### Outcomes

HR and HRV were analyzed. Spectral domain can be divided into two components: low-frequency (LF; 0.04–0.15 Hz) and high-frequency (HF; 0.15–0.4 Hz). LF reflects sympathetic and parasympathetic activities and HF reflects parasympathetic activities. The LF/HF ratio operates as an indicator of sympathetic nervous system activity related to increased stress level [20]. Questionnaires were e-mailed to participants on day 7 for IES-R and at one month for PCLS. Participants were asked to respond within two days. IES-R score ranged from 0 to 88 and PCLS from 17 to 85. PTSD was defined by IES-R ≥ 36/88 [28] and/or PCLS ≥ 44/85 [29].

### Statistics

Analysis was performed with Statview version 4.5 (SAS Institute Inc., Cary, NC). The number of required subjects was calculated by the methodologist of the Statistical Department and Clinical Investigation Center (CIC 1402) to highlight a possible relationship between stress and performance, which was considered to be significant if the correlation coefficient R2 reached a minimum value of 0.50 [21]. For a risk of the first kind at 5%, with a power of 90% and a bilateral situation, the number of subjects to be included was calculated at 48 (Proc POWER, SAS). Quantitative variables were described as mean ± standard deviation, and categorical variables were summarized by raw number (n) and percentage (%). The Kolmogorov-Smirnov test was used to check for normal distribution for assessed measures. Time variations ((measure–baseline)/baseline) of electrophysiological markers of stress were compared between baseline (T0, determined during deep sleep) and the different times before, during and after simulation (T1 to T7). Analysis of time variation was carried out to avoid individual variability in physiological conditions when comparing participants' stress response evolution. Stress measurement evolution between initial, intermediate, and final simulation sessions (time effect), and in the two groups (group effect) used ANOVA for repeated measures (RM-ANOVA). Post hoc multiple comparisons using the Scheffe test were carried out to compare groups at different times in order to explore any significant Group x Time interaction effects in the ANOVA. Hedge’s g test was used to analyze size effect on the standardized mean difference evolution of stress measurement between the two groups in the initial, intermediate, and final simulation sessions [30]. It corrects for bias in small samples of independent groups [31]. Thresholds for effect size were small for 0.2≤g≤0.5, medium for 0.5≤g≤0.8 and large for g≥0.8 [32]. Stress responses were compared between professional groups (status effect), and between males and females (gender effect) using ANOVA. p <0.05 was considered significant.

## Results

### Population

There were 26 men (54.2%) and 22 women (45.8%). The experimental group included 11 men (45.8%) and 13 women (54.2%), and the control group was composed of 14 men (58.3%) and 10 women (41.7%). The distribution in both groups was similar (p = 0.39). Fifteen participants were smokers (31.2%) with similar distribution in both groups (respectively 9 and 6 in the experimental and control groups, p = 0.16). According to the recommendations of the WHO on physical activity throughout the week [33], 34 participants engaged in at least 150 minutes of moderate-intensity aerobic activity or at least 75 minutes of vigorous-intensity aerobic activity or an equivalent combination. Major engagement in sporting activities (e.g. marathon, more than 5 hours throughout the week) concerned 7 participants (14.6%). Practice of yoga or meditation concerned 3 participants (6.2%), while 6 participants had no physical activity (12.5%). The test conducted across all four categories of professional status (Table 1) found no significant difference between the two groups for number of years of EMS experience (p = 0.21), and parameters of HR and HRV before the first simulations (p = 0.91 and p = 0.69 respectively). There was a significant gender difference for heart rate (60.02±8.20 for men and 71.40±8.25 for women, p<0.001), and LF/HF ratio (3.59±1.70 for men and 2.45±1.22 for women, p = 0.006). Participants were questioned about very extreme stressors; no participant had expressed a stressful event the day prior or the day of the simulation.

**Table 1:** Characteristics of participants according to professional status.

(n = 48)	Physicians	Residents	Nurses	Ambulance drivers	p
**Number of year of experience in EMS**	4.3±1.9	NA	5.2±1.9	5.6±1.4	0.21
**HR**	65±7	66±10	64±11	65±10	0.91
**LF/HF ratio**	3.2±1.7	3.1±1.3	2.9±1.3	2.9±2.0	0.69

EMS: Emergency Medical Services; HR: Heart rate (bpm); LF/HF ratio: analysis of heart rate variability in spectral domain: Low frequency (0.04–0.15Hz) and high frequency (0.15–0.45Hz) ratio. Comparison between the four professional status used ANOVA and p<0.05 was considered significant.

### Primary objective: 24-hour Holter evolution over one year

#### Analysis of HR and HRV during simulation

**Fig 3:**
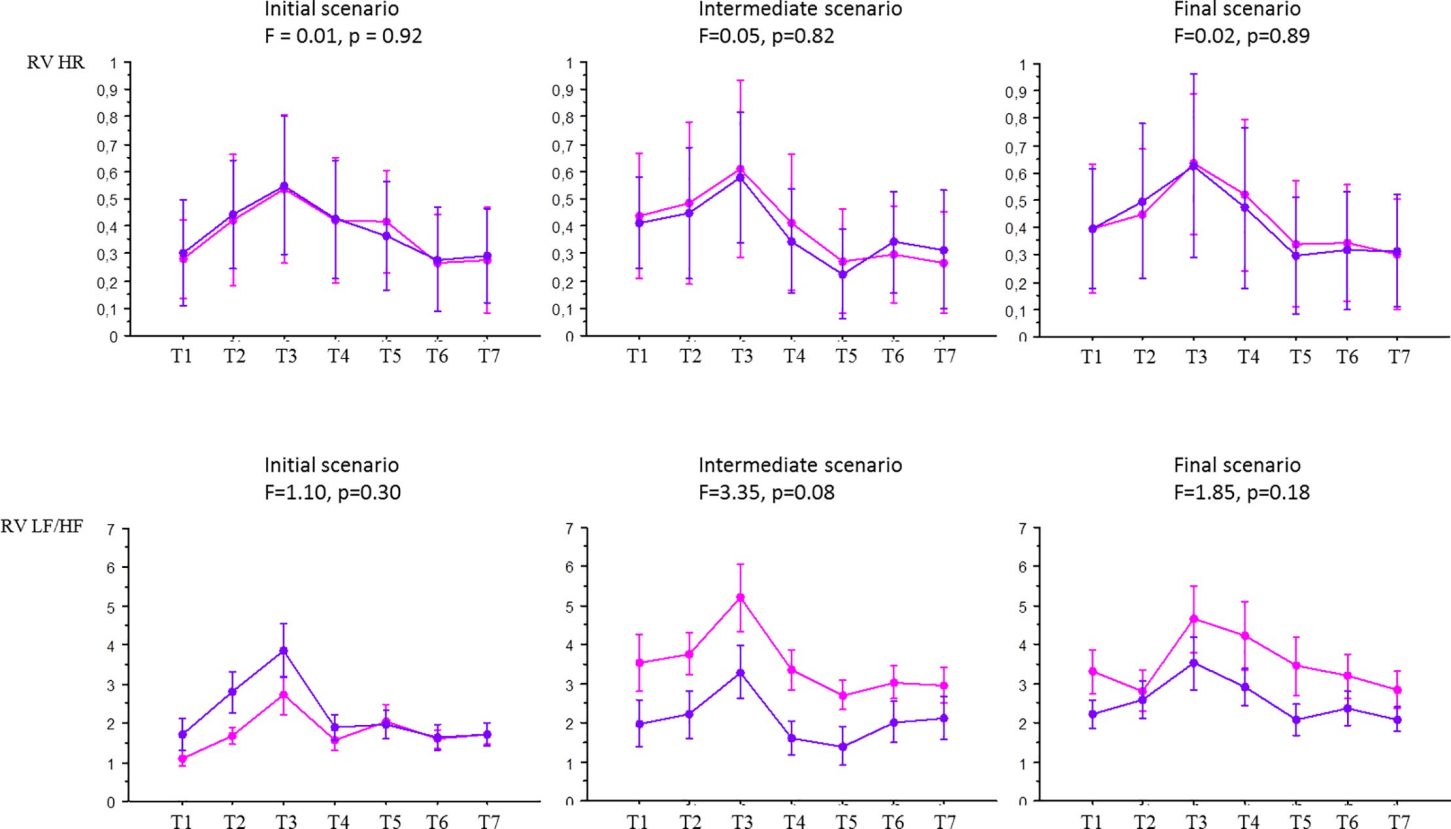
Comparison of the evolution of HR and HRV with repeated simulation sessions in the experimental and the control groups pertaining to each and every one of the seven times T1 through T7. Designated with a pink circle: Experimental group (n = 24) Designated with a blue circle: Control group (n = 24) HR: heart rate; LF/HF: LF/HF ratio (heart rate variability in frequency domain); RV: relative variation; T1: day prior to simulation; T2: before simulation; T3: during simulation; T4: after simulation; T5: before debriefing; T6: after debriefing; T7: 30min after debriefing. ANOVA was used to compare the evolution of HR and HRV in the two groups. F and p value are given for each comparison.

Relative variation of HR and HRV in frequency domain (i.e. LF/HF ratio) was analyzed at T1 to T7 during the 72 simulations. HR and LF/HF ratio increased during all the simulation sessions and they all decreased during and after debriefing sessions (F = 63.1, p<0.0001 for HR, and F = 26.9, p<0.0001 for LF/HF ratio). The Scheffe post-hoc test found a significant difference in all sessions between T2 and T3, and T3 and T5. In other words, stress level significantly increased during simulation whatever the repetition regimen of simulations– 3 or 9. There was no significant difference between the two groups during the initial, intermediate, and final simulation sessions for HR and LF/HF ratio (Fig 3). Comparison of HR using ANOVA was: F = 0.01, p = 0.92 for the initial session, F = 0.05, p = 0.82 for the intermediate session, and F = 0.02, p = 0.89 for the final session. Comparison of HRVs using ANOVA was: F = 1.10, p = 0.30 for the initial session, F = 3.35, p = 0.08 for the intermediate session, and F = 1.85, p = 0.18 for the final session.

#### Analysis of HR and HRV over 24 hours

There was no significant difference in HR between the experimental and the control group during the day of the three common simulation sessions. No significant difference was initially found for the LF/HF ratio. A significant variation was found for HRV between the two groups in diurnal and nocturnal periods after the initial session. There existed an interaction between group and time in LF/HF ratio evolution between initial and intermediate sessions, and also between intermediate and final sessions in diurnal period (p = 0.04, g = 0.61) and in nocturnal period (p = 0.01, g = 0.82). Significant interaction means that LF/HF ratio evolution over time is not the same between the two groups. LF/HF ratio was higher in the control group for the intermediate and final sessions. All results are given on Table 2.

**Table 2:** Comparison of Holter parameters between experimental and control groups during the common simulation sessions.

Period	Holter parameters	Experimental group (n = 24)			Control group (n = 24)			ANOVA	Hedge’s g test
		Initial	Intermediate	Final	Initial	Intermediate	Final		
		(M±SD)	(M±SD)	(M±SD)	(M±SD)	(M±SD)	(M±SD)	p	
Diurnal	HR	78.03±7.49	77.13±10.37	77.33±10.98	79.30±7.21	79.26±8.49	77.91±7.99	a = 0.54	
								b = 0.68	
								c = 0.81	
Diurnal	LF/HF ratio	4.70±2.32	4.72±2.12*	4.55±2.27*	4.89±2.05	5.18±3.21*	5.64±3.42*	a = 0.69	0.61
								b = 0.62	
								**c = 0.04**	
Nocturnal	HR	66.23±10.03	64.71±12.25	65.51±11.23	64.46±9.72	62.78±9.26	63.10±10.34	a = 0.42	
								b = 0.54	
								c = 0.97	
Nocturnal	LF/HF ratio	2.93±1.26	3.48±1.99*	3.21±1.39*	3.15±1.86	4.05±2.95*	3.92±2.86*	a = 0.37	0.82
								b = 0.60	
								**c = 0.01**	

ANOVA: ANOVA for repeated measures (RM-ANOVA); a: group effect; b: time effect; c: interaction (group x time) effect

*: Post hoc multiple comparisons using the Scheffe test were carried out to compare groups at different times in order to explore any significant Group x Time interaction effects in the ANOVA (p < 0.05). Hedge’s g test: analyze of size effect on the standardized mean difference evolution.

HR: heart rate; LF/HF: analysis of heart rate variability in spectral domain: Low frequency (0.04–0.15Hz) and high frequency (0.15–0.45Hz) ratio.

M±SD: mean ± standard deviation. Simulations common to the experimental and control groups: initial: the first day; intermediate: after 6 months; final after 1 year.

### Secondary objectives

#### Comparative analysis of HR and HRV

There was no significant difference in HR and HRV according to professional status. HR and HRV were significantly different between males and females. There was a significant difference for HR in diurnal and nocturnal periods and for HRV in diurnal period according to gender (Table 3).

**Table 3:** Study of status and gender effects on Holter parameter evolution.

(n = 48)	Status effect (F = )	p	Gender effect (F = )	p
**HR Diurnal period**	0.38	0.78	4.46	**0.04**
**LF/HF Diurnal period**	0.67	0.58	4.80	**0.04**
**HR Nocturnal period**	0.24	0.86	11.01	**0.004**
**LF/HF Nocturnal period**	2.62	0.08	1.39	0.25

HR: heart rate (bpm); LF/HF: analysis of heart rate variability in spectral domain: Low frequency (0.04–0.15Hz) and high frequency (0.15–0.45Hz) ratio.

Status effect: difference in Holter parameter evolution according to professional status (emergency physician, resident, nurse, ambulance driver); gender effect: difference in Holter parameter evolution according to gender.

F test: Comparison of stress responses according to professional status and gender using ANOVA. p<0.05 was considered significant.

#### Perceived stress

**Fig 4:**
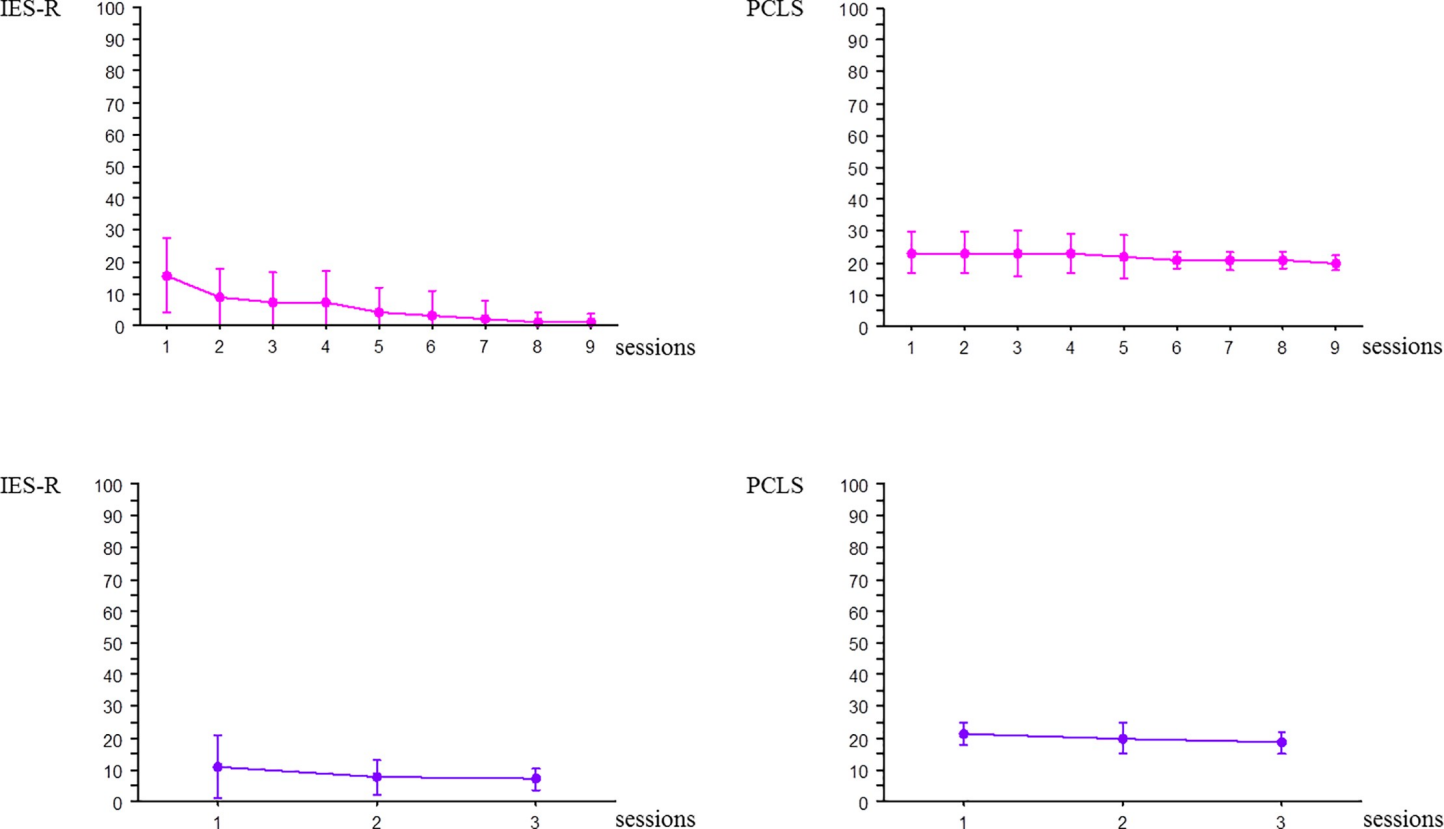
Evolution of IES-R and PCLS scores over time during the 9 simulation sessions in the experimental group and the 3 simulation sessions in the control group. Designated with a pink circle: Experimental group (n = 24) Designated with a blue circle: Control group (n = 24) IES-R: Impact of Event Scale–Revised; PCLS: Post-traumatic Check-List Scale.

Rate of response was 90% and 89% for IES-R and PCLS questionnaires respectively. The first 6 months, i.e. the 5th session for experimental group and the 2nd session for the control group, all participants completed both questionnaires. Then rate of response was 76% for IES-R and 73.6% for PCLS. No participant developed PTSD. IES-R score was 11.7±1.9 with a maximum of 34 and PCLS score was 19.0±0.8 with a maximum of 29. Scores were analyzed according to group, professional status, and gender. In the experimental group, a significant decrease in IES-R was observed (F = 4.92, p<0.0001). The Scheffe post-hoc test found a significant difference between the 1st session and the following one from the 5th session onward. The PCLS score remained unchanged (F = 1.06, p = 0.39). Starting with the 5th session, standard deviations decreased and there was less heterogeneity between individuals for both IES-R and PCLS scores (Fig 4). No variation was present in the control group for IES-R score (F = 2.86, p = 0.07) or PCLS score (F = 0.86, p = 0.42) (Fig 4). The control group’s lack of reduction in IES-R scores from session 2 (intermediate session) to 3 (final session) contrasted with the experimental group’s continual and significant reduction in IES-R scores from sessions 5 through 9. There was no status effect, either for IES-R score (F = 2.10, p = 0.10) or PCLS score (F = 2.16, p = 0.09). There was no gender significant difference for IES-R score (F = 0.58, p = 0.80) and PCLS score (F = 0.48, p = 0.87).

## Discussion

### Main results

All teams had similar composition including physician, resident, nurse, and ambulance driver with a similar experience. Distribution of smokers was similar in the two groups. All teams performed simulations in similar conditions and environment. There was no significant difference between the two groups in HR and HRV before the first simulation and over the 24-hour period of the initial session. Consequently, and even if there is individual variation, experimental and control groups can be compared and no adjustment was carried out. Holter parameter evolution was different according to frequency of repetition of simulations. On the other hand, an effect due to interaction between time and group was noted: in the 24-hour period of the intermediate and final sessions, LF/HF ratio was significantly lower in the experimental group than in the control group, reflecting a lower level of stress. Stress response varied according to gender (HR was higher in females whereas LF/HF was higher in males). There was no significant difference according to professional status. No participant developed PTSD, but IES-R score decreased in the experimental group with greater homogeneity between participants after the 5^th^ session. To our knowledge, no simulation study has previously studied the occurrence of PTSD in the SBT environment.

### Primary objective

HRV has been used in simulation studies for objective assessment of stress in time domain [34], in frequency domain [35] or in both [18]. We previously demonstrated that during HSF of pediatric life-threatening events, stress level, assessed by electrophysiological parameters and salivary cortisol, increased in all participants and decreased during and after debriefing, whatever the frequency of repetition of sessions [14,18]. We also found that HR and HRV in the frequency domain (LF/HF ratio) increased during all repeated sessions in both groups. In another study pertaining to the same randomized control trial, results suggested that salivary cortisol similarly increased during all repeated simulations and decreased during debriefing in both experimental and control groups [19]. On this subject, the Yerkes-Dodson law on tendency for optimal performance models the relationship between performance and stress as an inverted-U curve: stress improves performance up to a peak, and subsequently drives it down [36]. Applying this law to our results, we speculate that stress might be an adaptive response helping to improve performance during management of life-threatening situations. In the present study, stress persisted during all simulations. To our knowledge, the impact of repeated stress exposure on participants during SBT had not previously been studied. Based on the analysis of the 24-hour Holter, a different LF/HF ratio evolution over time in the experimental and control groups was found. We speculate that despite the stress generated by simulation, the more the sessions were repeated, the less the impact on participants' daily lives, a finding reflected by lower ANS activity. Our interpretation was that a standardized simulation with a full briefing and debriefing with good judgment [26] allowed participants to get used to simulation sessions when they were repeated every 6 weeks. Consequently, even in the case of increased stress during simulation as an adaptive response, ANS activity decreased during the day. We concluded that participants were less nervous about simulation. On the other hand, when sessions were repeated only once every 6 months, habituation did not occur and ANS activity remained identical over the 24-hour periods during the 1^st^, 2^nd^, and 3^rd^ sessions. Interpretation of electrophysiological measurements should be reinforced with other measurements insofar as the LF/HF ratio does not constantly represent a dominance of sympathetic nervous system activity. Salivary alpha-amylase activity or plasma noradrenaline concentrations are often considered to be surrogate markers of sympathetic activation in response to stress, and as biological markers, they can be of use [37].

### Secondary objectives

Participants with differing habits (smoking, physical activity …) may render ANS parameters heterogeneous. In addition to HR and HRV, analysis of time variation for each participant i.e. relative variation of HR and HRV between T0 and other times of measurement facilitated comparison of stress response. The present study showed, as previously described, that all participants exhibited a similar stress response irrespective of their status [14,38]. As expected, there were significant gender differences according to stress response [39,40]. These results suggest that since higher stress is invariably expressed during simulation, stress response should be studied in all team members [14]. And due to the heterogeneity of stress response, gender should be considered as an independent variable in all mixed-gender studies on simulation [41]. All in all, future study should analyze performance evolution according to the participants’ gender, speculating that relationship between performance and stress evolution might differ according to the gender since stress response is different.

Despite repeated simulations, no participant developed PTSD. The National Center for PTSD suggests a cutpoint range of 30 to 35 for a general population using the PCL for DSM 4. When a cutpoint of 44 is used, sensitivity is 97% and specificity is 87%, whereas for a cutpoint of 34 sensitivity is 78% and specificity is 94% [42]. All participants had a score below 30. The use of standardized debriefing with “good-judgment” [26] may have contributed to the absence of PTSD, as suggested by a decrease in HR and LF/HF ratio during all the debriefings. Moreover, simulated death is a useful tool enabling learners to cope with the death of patients [43] and to improve performance [44]. Conversely, emotional reactions to simulated death can have a serious psychological impact, particularly when death results from the actions or inaction of learners [43]. Repeated exposure to mortality in simulation may unnecessarily increase the level of anxiety a participant associates with simulation [44], whereas non-occurrence of death of the simulated patient in our experiments may have helped to limit risk of PTSD. However, there is considerable controversy over whether to allow a manikin to die. Many experts in simulation recommend that if death is a possibility, trainees should be informed of this eventuality before each session [43,45]. Other authors find death of the manikin to be acceptable and even beneficial to trainees, and conclude that it need not be preliminarily announced [46]. Nevertheless, death of the manikin was stressful to participants in this study [46]. More broadly, it may impact performance. Some authors have suggested that cognitive load is increased [47] whereas others consider it compromised [48], and still others have found that it has little or no impact on performance [46]. Consequently, in order to avoid bias in comparison between two groups, and when analyzing performance or stress evolution during repeated sessions, all simulations should be carried out under conditions as similar to each other as possible. A future study should analyze the impact of manikin death on anxiety and PTSD when simulations are repeated.

Regarding the evolution of PTSD markers, we found a significant difference between the two groups at the 7^th^ day: a significant decrease in IES-R was observed when simulations were repeated every 6 weeks, whereas the scores remained stable when they were repeated every 6 months. These results, combined with decreased ANS activity on the day of simulation when they were repeated every 6 weeks, suggest that the sessions may have been experienced as non-traumatic. Due to possible habituation and to the standardized conditions and settings of simulation, they did not interfere with daily life. Starting with the 5th session, standard deviation of PTSD score decreased and there was less heterogeneity between individuals in both IES-R and PCLS scores, thereby reinforcing the hypothesis that repeated simulation of management of life-threatening situations yields no psychological consequences. On the other hand, repetition of simulation every 6 months might have been experienced similarly during all 3 sessions because they were too distant in time from one another. Consequently, stable ANS activity on the day of simulation and an unchanged IES-R score were observed. The low PCLS score at one month seemed to show that simulation performed under increased stress had no psychological impact. Therefore, repeated SBT in life-threatening emergency management may entail little or no risk for learners. Finally, it seemed that by the 7^th^ simulation session, all of the experimental group participants were responding with the lowest possible value on all IES-R items. We hypothesized that the repeated stress exposure during simulations might provide protection from the development of PTSD symptoms that arise due to the real-life traumatic events to which team members are exposed in the performance of their duties. Indeed, decreased HR and HRV associated with a lower level of IES-R suggests that exposure to stressors during repeated SBT could aid in stress coping and shift stress from a maladaptive to an adaptive level. Exposure to stressors elicits a spectrum of responses that are particularly pronounced in the hippocampus, where they also appear to influence hippocampal-dependent cognitive function and emotionality [49]. Factors that influence the consequences include the chronicity, severity, predictability and controllability of the stressors [49]. A future study should investigate the impact of repeated simulation training on the stress felt and experienced physiologically in real-life situations.

### External validity

We think that the study design assessing management of an infant in shock by an EMS team could be applicable to other medical situations requiring MDT management of life-threatening events in stressful environments: emergency room, intensive care unit, delivery room or operating room. Therefore, the results we have reported could possibly be generalized to simulated training of emergency physicians, intensivists, obstetricians, anesthesiologists, neonatologists, or surgeons concerned by the unpredictable situations inherent to life-threatening events.

### Limitations

We are aware that the present study is not without limitations. Firstly, the LF/HF ratio does not constantly represent a dominance of sympathetic nervous system activity; the LF component does not necessarily and systematically reflect sympathetic activity under any and all conditions. Moreover, the contributions of sympathetic activity to this metric vary substantially according to testing conditions, of which the variability may lead to differing interpretations [50,51]. HR and HRV evolution can be affected by factors such as diet, exercise and activities. In our study, the weight of these factors was limited by prohibition of a night-shift prior to the simulation day, a measure taken in order to ensure nighttime sleep. Sports activities were likewise prohibited from the day prior to simulation until the end of simulation. Participants were also asked if they had experienced a serious or stressful event the day before or on the day of the simulation. However, a lack of accounting for exposure to real-life stressors over a 12-month period (e.g., witnessing sudden patient deaths) may have prevented the present study from exhaustively interpreting stress response. And given the small sample size, randomization may not have been sufficient to overcome random noise associated with stressors outside the study. In addition, the fact that all participants were filmed and observed may have increased their stress level. Some participants said they were bothered by the electrodes set up to record Holter parameters, and they may have modified their level of stress. However, this factor could not have changed the relative variation of the stress parameters allowing for study of stress response. There was a significant gender difference and stress response would have been less heterogeneous if the study had been limited to either male of female participants. However, this would not have reflected the real-life situations encountered by mixed teams managing emergencies. Finally, the reduction in variance of PTSD scores (IES-R and PCLS) over time is remarkable. A lower rate of response after the first six months may have largely contributed to this result. However, rate of response was similar in the two groups and may not be adequately explain significantly different evolution of IES-R score over time in the two groups.

## Conclusion

The present study is the first to demonstrate that stress during HFS of life-threatening events is recurrent when simulation sessions are repeated. The greater the frequency of repetition, the lesser the impact on trainees' daily lives. The relevant electrophysiological markers decreased over 24 hours, and stress did not lead to PTSD. These results are important, insofar as repeated simulations are aimed at improving performance and management of patients in critical situations and under stressful conditions. Future studies should assess the impact of stress when simulations are repeated more frequently.

## Supporting information

S1 Checklist CONSORT 2010 checklist. (DOC)

S1 File Protocole SIM-STRES-2 du 16.07.2013. (PDF)

S2 File SIM_STRES_Protocol for CPP and ANSM_english_version. (PDF)

## Abbreviations

ANS: autonomic nervous system
HF: high-frequency
HFS: high-fidelity simulation
HRV: heart rate
IES-R: Impact of Event Scale-Revised
LF: low-frequency
MDT: multidisciplinary team
PCLS: Post-traumatic Check-List Scale
PTSD: post-traumatic stress disorder
SBT: simulation-based training
