# Supplementary material for: Stress response in the daily lives of simulation repeaters. A randomized controlled trial assessing stress evolution over one year of repetitive immersive simulations
Source: PLoS One. 2019 Jul 25;14(7):e0220111. doi: 10.1371/journal.pone.0220111 (PMC6657860; doi:10.1371/journal.pone.0220111)
Supplement: S1 File — (PDF) [file pone.0220111.s002.pdf]

Effets du stress et de la simulation sur la performance d'une équipe lors de la prise en charge d'un nourrisson en état de choc

Sim-Stress

Version n°2 en date du 16/07/2013

PROMOTEUR
Centre Hospitalier Universitaire de Poitiers
2 rue de la Milétrie
BP 577
86021 POITIERS cedex
Tél : 05.49.44.46 65
Fax : 05.49.44.30.58

INVESTIGATEUR COORDONNATEUR
Dr Daniel Aiham GHAZALI
Urgences – SAMU86
Centre Hospitalier Universitaire de Poitiers
2 rue de la Milétrie
BP 577
86021 Poitiers cedex
Tél : 05 49 44 37 08 (CHU) / 05 49 45 43 87 (UFR Médecine)
Fax : 05 49 44 39 01
E-mail : a.d.ghazali@chu-poitiers.fr

METHODOLOGISTE
Dr Stéphanie RAGOT
CIC – INSERM 0802
Centre Hospitalier Universitaire de Poitiers
2 rue de la Milétrie - 86021 Poitiers cedex
Tél : 05 49 44 49 13 / Fax : 05 49 44 46 91
E-mail : s.ragot@chu-poitiers.fr

CE DOCUMENT CONFIDENTIEL EST LA PROPRIETE DU CHU DE POITIERS

**PROMOTEUR :**	**Centre Hospitalier Universitaire de Poitiers**<br>2 rue de la Milétrie-BP 577<br>86 021 Poitiers cedex<br>Tél : 05.49.44.46.65<br>Fax : 05.49.44.30.58
**PROTOCOLE D'ESSAI CLINIQUE**	
**CODE ESSAI**<br><br>**N° ID-RCB**	Sim-Stress<br><br>2013-A00648-37
**TITRE COMPLET**<br><br><br><br>**INDICATION(S) (CIBLE)**	Effets du stress et de la simulation sur la performance d'une équipe lors de la prise en charge d'un nourrisson en état de choc<br><br>Non applicable
**INVESTIGATEUR COORDONNATEUR**	Dr Daniel Aiham GHAZALI<br>Centre Hospitalier Universitaire de Poitiers<br>Service de Urgences-SAMU86<br>2 rue de la Milétrie- BP 577 - 86021 Poitiers cedex<br>Tél : 05 49 44 37 08 / Fax : 05 49 44 39 01<br>E-mail : a.d.ghazali@chu-poitiers.fr
**CPP**	*Approuvé le …*<br>*Par le Comité de Protection des Personnes ouest III.*
**ANSM**	Date d'autorisation :<br>N° d'autorisation :

CE <u>DOCUMENT CONFIDENTIEL</u> EST LA PROPRIETE DU CHU DE POITIERS.
AUCUNE INFORMATION NON PUBLIEE FIGURANT DANS CE DOCUMENT NE PEUT ETRE DIVULGUEE SANS AUTORISATION ECRITE PREALABLE DU CHU DE POITIERS.

SOMMAIRE

PROTOCOLE D'ESSAI CLINIQUE 2
SOMMAIRE 3
SIGNATURES 6
RESUME 7
1. INFORMATIONS GENERALES 10
1.1. Titre 10
1.2. Promoteur 10
1.3. Coordination et suivi de l'etude 10
1.4. Investigateurs 10
1.5. Vigilance 11
1.6. Data-management 11
1.7. Methodologiste - biostatisticien 11
2. JUSTIFICATION SCIENTIFIQUE ET DESCRIPTION GENERALE DE L'ETUDE 12
2.1. Denomination et description de la situation 12
2.2. Generalites 12
2.3. Le travail en equipe 12
2.4. Le stress 13
2.5. La simulation 14
2.6. Resume des benefices, le cas echeant, et des risques previsibles pour les personnes se pretant a la recherche 14
2.6.1. Bénéfices 14
2.6.2. Risques 14
2.6.3. Balance bénéfices / risques 15
2.7. Declaration du respect du protocole, des bonnes pratiques cliniques et des dispositions legislatives et reglementaires en vigueur dans le cadre de ce protocole 15
2.8. References 15
3. OBJECTIFS DE L'ETUDE 18
3.1. Objectif principal 18
3.2. Objectifs secondaires 18
4. CONCEPTION DE L'ETUDE 18
4.1. Critere d'evaluation principal 18
4.2. Criteres d'evaluation secondaires 18
4.2.1. Evaluation du stress 18
4.2.2. Evaluation de l'évolution de la performance 19
4.3. Description des interventions 19
4.4. Deroulement de l'etude 21
4.4.1. Schéma général de recherche lors d'une séance de simulation en équipe 21
4.4.2. Quantification des séances et des différentes évaluations 21
4.5. Description des mesures prises pour reduire et eviter les biais 22
4.5.1. Tirage au sort 22
4.5.2. Homogénéité de la population d'étude 22
4.5.3. Méthodes d'évaluation par échelles d'hétéro-évaluation 22
4.6. Duree de l'etude 22
4.7. Description des regles d'arret definitif ou temporaire 23
4.7.1. Arrêt de participation d'un participant dans cette étude 23
4.7.2. Arrêt d'une partie ou de la totalité de la recherche 23
4.8. Identification de toutes les donnees a recueillir directement dans le cahier d'observation, qui seront considerees comme des donnees sources 23
5. CRITERES D'INCLUSION ET DE NON INCLUSION 23
5.1. Criteres d'inclusion 23
5.2. Criteres de non inclusion 24
5.3. Criteres de recrutement 24
5.4. Procedure d'exclusion de la recherche 24
5.4.1. Critères et modalités d'exclusion d'une personne de la recherche 24
5.4.2. Modalités et calendrier de recueil pour ces données 25
5.4.3. Modalités de suivi de ces personnes 25
6. ANALYSES 25

6.1. EVALUATION DE L'EFFET DU STRESS SUR LA PERFORMANCE 25
6.2. EVALUATION DE L'EFFET DE LA REPETITION DES SEANCES DE SIMULATION 25
7. EVALUATION DE LA PERFORMANCE ET DU STRESS 25
7.1. DESCRIPTION DES PARAMETRES D'EVALUATION DE LA PERFORMANCE 25
7.2. DESCRIPTION DES PARAMETRES D'EVALUATION DU STRESS 26
7.3. METHODES ET CALENDRIER PREVUS POUR MESURER, RECUEILLIR ET ANALYSER LES PARAMETRES D'EVALUATION DE LA PERFORMANCE 26
7.3.1. Mesure de la performance 33
7.3.2. Mesure du stress 26
8. EVALUATION DE LA SECURITE 26
8.1. PARAMETRES D'EVALUATION DE LA SECURITE 26
8.2. METHODES ET CALENDRIER PREVUS POUR MESURER, RECUEILLIR ET ANALYSER LES PARAMETRES D'EVALUATION DE LA SECURITE 27
8.3. PROCEDURES MISES EN PLACE EN VUE DE L'ENREGISTREMENT ET DE LA NOTIFICATION DES EVENEMENTS INDESIRABLES 27
8.3.1. Définitions 27
8.3.2. Rôle de l'investigateur 27
8.3.3. Rôle du promoteur 29
8.3.4. Exposition in utero 30
8.3.5. Comité de surveillance 30
8.4. MODALITES ET DUREE DU SUIVI DES PERSONNES SUITE A LA SURVENUE D'EVENEMENTS INDESIRABLES 30
9. STATISTIQUES 30
9.1. DESCRIPTION DES METHODES STATISTIQUES PREVUES, Y COMPRIS DU CALENDRIER DES ANALYSES INTERMEDIAIRES PREVUES 30
9.1.1. Analyse descriptive 30
9.1.2. Evaluation de l'effet du stress sur la performance 31
9.1.3. Evaluation des variations des scores de performance 31
9.1.4. Comparaison de la variation des scores de performance entre les groupes A et B = évaluation de l'effet de la simulation répétée sur la performance 31
9.1.5. Comparaison de la variation des scores de performance entre les groupes A et B = évaluation de l'effet de la simulation répétée sur le stress 31
9.1.6. Reproductibilité inter-observateurs 31
9.2. NOMBRE PREVU DE PATIENTS A INCLURE DANS CETTE ETUDE ET JUSTIFICATION STATISTIQUE 31
9.3. DEGRE DE SIGNIFICATION STATISTIQUE PREVU 32
9.4. CRITERES STATISTIQUES D'ARRET DE LA RECHERCHE 32
9.5. METHODE DE PRISE EN COMPTE DES DONNEES MANQUANTES, INUTILISEES OU NON VALIDES 32
9.6. GESTION DES MODIFICATIONS APPORTEES AU PLAN D'ANALYSE DE LA STRATEGIE INITIALE 32
10. DROIT D'ACCES AUX DONNEES ET DOCUMENTS SOURCE 32
10.1 ACCES AUX DONNEES 32
10.2 DOCUMENTS SOURCE 32
10.3 CONFIDENTIALITE DES DONNEES 32
11. CONTROLE ET ASSURANCE DE LA QUALITE 33
12. CONSIDERATIONS ETHIQUES 33
12.1 COMITE DE PROTECTION DES PERSONNES 33
12.2 MODIFICATIONS SUBSTANTIELLES 33
12.3 INFORMATION DU PATIENT ET FORMULAIRE DE CONSENTEMENT ECLAIRE ECRIT 33
12.4 DEFINITION DE LA PERIODE D'EXCLUSION 34
12.5 PRISE EN CHARGE RELATIVE A LA RECHERCHE 34
12.6 INDEMNISATION DES SUJETS 34
12.7 INSCRIPTION AU FICHIER NATIONAL DES PERSONNES SE PRETANT A UNE RECHERCHE BIOMEDICALE 34
13. TRAITEMENT DES DONNEES ET CONSERVATION DES DOCUMENTS ET DONNEES 34
13.1. CAHIER D'OBSERVATION 34
13.2. SAISIE ET TRAITEMENT DES DONNEES 34
13.3. CNIL 34
13.4. ARCHIVAGE 35
14. ASSURANCE 35
15. FAISABILITE DE L'ETUDE 35
16. REGLES RELATIVES A LA PUBLICATION 35

17. PROPRIETE – EXPLOITATIONS DES RESULTATS ET VALORISATION ... 36
18. LISTE DES ANNEXES ... 36
18.1. NOTICE D'INFORMATION DU PARTICIPANT A LA RECHERCHE ... 37
18.2. FORMULAIRE DE CONSENTEMENT DU PARTICIPANT A LA RECHERCHE ... 40
18.3. DECLARATION D'HELSINKI ... 41
18.4. FORMULAIRE DE DECLARATION D'UN EVENEMENT INDESIRABLE GRAVE ... 46
18.5. ECHELLE D'EVALUATION DE LA PERFORMANCE GLOBALE D'UNE EQUIPE ... 48
18.6. ECHELLE D'EVALUATION DE LA POSE D'UNE VOIE INTRA-OSSEUSE ... 51
18.7. ECHELLE D'EVALUATION DU LEADERSHIP BAT ... 53
18.8. ECHELLE CLINIQUE DU TRAVAIL EN EQUIPE CTS ... 54
18.9. ECHELLE DE MESURE DE L'ANXIETE STAI ... 55
18.10. QUESTIONNAIRE DE STRESS POST-EVENEMENTIEL IES-R ... 56
18.11. QUESTIONNAIRE DE STRESS POST-EVENEMENTIEL PCLS ... 57
18.12. STRATEGIES DE GESTION DU STRESS ... 58

SIGNATURES

SIGNATURE DE L'INVESTIGATEUR

J'ai lu l'ensemble des pages du protocole de l'essai clinique dont le CHU de Poitiers est le promoteur. Je confirme qu'il contient toutes les informations nécessaires à la conduite de l'essai. Je m'engage à réaliser l'essai en respectant le protocole et les termes et conditions qui y sont définis. Je m'engage à réaliser l'essai en respectant :

- les principes de la "Déclaration d'Helsinki",
- les règles et recommandations de bonnes pratiques cliniques internationales (ICH-E6) et française (règles de bonnes pratiques cliniques pour les recherches biomédicales portant sur des médicaments à usage humain - décisions du 24 novembre 2006),
- la législation nationale et la réglementation relative aux essais cliniques,
- la conformité avec la Directive Essais Cliniques de l'UE [2001/20/EC] dont une copie de chaque m'a été remise par le promoteur.

Je m'engage également à ce que les investigateurs et les autres membres qualifiés de mon équipe aient accès aux copies de ce protocole et des documents relatifs à la conduite de l'essai pour leur permettre de travailler dans le respect des dispositions figurant dans ces documents.

NOM: Dr Daniel Aiham GHAZALI

Signature : …………………………………………….. Date : ___________________

SIGNATURE DU PROMOTEUR

Promoteur :

NOM : Mr. Jean-Pierre DEWITTE

Signature : ……………………………………………… Date : ___________________

RESUME

TITRE	Effets du stress et de la simulation sur la performance d'une équipe lors de la prise en charge d'un nourrisson en état de choc
PROMOTEUR	Centre Hospitalier Universitaire de Poitiers<br>2 rue de la Milétrie - BP 577<br>86021 Poitiers cedex<br>Tél : 05.49.44.46.65<br>Télécopie : 05.49.44.30.58
INVESTIGATEUR	Daniel Aiham GHAZALI<br>Centre Hospitalier Universitaire de Poitiers<br>Urgences – SAMU86<br>2 rue de la Milétrie- BP 577 - 86021 Poitiers cedex<br>Tél : 05 49 45 37 08 / Fax : 05 49 44 39 01<br>E-mail : a.d.ghazali@chu-poitiers.fr
JUSTIFICATION / CONTEXTE	La sûreté des patients dépend de l'efficacité des gestes, du respect des algorithmes et de la qualité du travail en équipe. Le stress modifie tous ces paramètres. Nous étudierons en simulation la prise en charge pluridisciplinaire d'un nourrisson en état de choc nécessitant la pose d'une voie intra-osseuse (VIO), ainsi que l'effet de la répétition des simulations en climat de stress.
OBJECTIF PRINCIPAL	Evaluer ***l'effet du stress sur la performance*** d'une équipe, avec trois volets :<br>o La performance globale de l'équipe : respect de l'algorithme de prise en charge et réalisation des gestes indiqués.<br>o La VIO : technique de pose et succès.<br>o Le travail en équipe : leadership et communication.
OBJECTIFS SECONDAIRES	***Evaluer l'effet de la simulation répétée sur l'évolution de la performance*** d'une équipe dans le maintien de la sûreté du nourrisson en état de choc, avec trois volets :<br>o La performance globale de l'équipe : respect de l'algorithme de prise en charge et réalisation des gestes indiqués.<br>o La VIO : technique de pose et succès.<br>o Le travail en équipe : leadership et communication.<br>***Evaluer l'effet de la simulation répétée sur le stress*** d'une équipe dans le maintien de la sûreté du nourrisson en état de choc, avec trois évaluations du stress :<br>o Le cortisol salivaire.<br>o Le Holter : variabilité de la fréquence cardiaque, intervalle RR' et autres paramètres. Des mesures ponctuelles de pression artérielle et fréquence cardiaques seront faites.<br>o Les questionnaires de stress STAI, IES-R et PCLS.
CRITERE DE JUGEMENT PRINCIPAL	Notre critère d'évaluation principal est la **performance de l'équipe** dans la prise en charge simulée d'un nourrisson en état de choc. Cela sera évalué de trois façons :<br>- La performance globale d'une équipe par le score obtenu sur l'échelle globale de performance en cours de validation (Oriot 2013) (cf. Annexe).<br>- La performance de la pose d'une VIO : par l'échelle d'évaluation IOPAS – *Intraosseous Performance Assessment Scale* (Oriot 2012), délais décisionnels et de mise en place (cf. Annexe).<br>- La performance du travail en équipe :<br>• Le leadership par l'échelle BAT – *Behavioural Assessment Tool* (Anderson 2010) (cf. Annexe).<br>• L'évaluation globale du travail en équipe par l'échelle CTS - *Clinical Teamwork Scale*, CTS (Guise 2008)] (cf.

	Annexe).
CRITERES DE JUGEMENT SECONDAIRES	Le stress qui sera évalué de 3 façons différentes :<br>- Biologique : dosage du cortisol salivaire par technique de Radio-Immuno-Essai (Radio-Immuno-Assay, RIA). Cette technique est validée et utilisée pour l'évaluation de l'effet du stress (Wagner 2010, Beko 2010, Dovio 2010). Ce dosage sera réalisé la veille, avant et après simulation ainsi qu'à la suite du débriefing.<br>- Electrophysiologique : mesure de la variation de la fréquence cardiaque, de sa variabilité, du pNN50 et du spectre de l'intervalle R-R' (Wilhelm 2005) de chaque participant par enregistrement Holter, avant (pendant environ 21h), pendant et après simulation (pendant 3 heures) (Task Force 1996). Acquisition de 4 appareils Holter (Sorin Group*). Mesures ponctuelles de la pression artérielle et de la fréquence cardiaques, reflets du système nerveux autonome. Ces mesures seront faites la veille, avant et après simulation ainsi qu'à la suite du débriefing.<br>- Psychologique :<br>• Mesure par le questionnaire d'auto-évaluation STAI – *State-Trait Anxiety Inventory* (Spielberger 1983) de l'état anxieux la veille, avant et après simulation ainsi qu'à la suite du débriefing (cf. Annexe).<br>• Mesure du stress post-événementiel précoce par l'échelle de stress IES-R – *Impact of Event Scale-Revised*, traduite et validée en français (Brunet 2003), 7 jours après la séance de simulation<br>• Mesure du stress post-événementiel durable par l'échelle PCLS – *Posttraumatic Check-List Scale* (Weathers 1993), un mois après la séance de simulation.
MÉTHODOLOGIE / SCHÉMA DE L'ETUDE	Recherche biomédicale interventionnelle avec prélèvements sur volontaires sains. Etude monocentrique à recrutement régional, prospective, randomisée, contrôlée.<br>Deux groupes randomisés A et B de 6 équipes, composées chacune de 4 personnes (1 médecin senior, 1 interne, 1 infirmière, 1 ambulancier), auront reçu préalablement le même enseignement théorique et pratique sur la pose d'une VIO. Le groupe A aura 9 simulations en 12 mois et le B, seulement 3.
CRITERES D'INCLUSION DES SUJETS	- La participation à la recherche se fera sur la base du volontariat.<br>- Age ≥ 18 ans.<br>- Sujet libre, sans tutelle ou curatelle ni subordination.<br>- Participants bénéficiant d'un régime de Sécurité Sociale.<br>- Consentement libre et éclairé.<br>- Accord écrit pour l'évaluation par vidéo à des fins exclusives de recherche.<br>- Equipe de quatre personnes :<br>1/- Médecin urgentiste de la région Poitou-Charentes et des alentours ayant une expérience professionnelle de moins de sept ans et ayant obtenu le Diplôme Universitaire des Gestes d'Urgence en Pédiatrie dans les 3 dernières années, faisant suite aux dernières recommandations de réanimation avancée pédiatrique établies en 2010 par l'AHA (American Heart Association) (Kleinman 2010) et l'ERC (European Resuscitation Council) (Barient 2010);<br>2/- Interne en médecine inscrit au DESC de Médecine d'Urgence à l'Université de Poitiers, formés aux gestes

	d'urgence en pédiatrie. Ils ont validés un stage clinique dans un service des urgences pédiatriques ou le Diplôme Universitaire des Gestes d'Urgence en Pédiatrie;<br>3/- Infirmier(e) du SAMU-SMUR du CHU de Poitiers ayant une expérience professionnelle de moins de sept ans et ayant obtenu le diplôme EPILS – European Pediatric Immediate Life Support – lors des deux dernières années ;<br>4/- Ambulancier(e) du SAMU-SMUR du CHU de Poitiers ayant une expérience professionnelle de moins de sept ans.
CRITERES DE NON-INCLUSION DES SUJETS	- Age < 18 ans<br>- Participation actuelle à une autre étude de recherche clinique avec une période d'exclusion d'un mois entre les deux protocoles de recherche<br>- Participants ne bénéficiant pas d'un régime de Sécurité Sociale<br>- Femmes enceintes ou allaitantes, femmes en âge de procréer ne disposant pas d'une contraception efficace (hormonal/mécanique : per os, injectable, transcutané, implantable, dispositif intra-utérin, ou bien chirurgical : ligature des trompes, hystérectomie, ovariectomie totale)<br>- Antécédents médicaux et/ou psychiatriques pouvant influencer l'état de stress.<br>- Antécédents cardiaques ou neurologiques convulsivants.<br>- Port de pacemaker ou de défibrillateur implantable.<br>- Participant prenant des médicaments cardiotropes et/ou des bronchodilatateurs β2-mimétiques (modification possible des paramètres électrophysiologiques cardiaques).
CRITERES D'EXCLUSION EN COURS D'ETUDE (SORTIES D'ETUDE)	- Non-respect des dates et horaires d'évaluation pouvant compromettre les séances de simulation ou influencer les paramètres étudiés.<br>- Exclusion de toute une équipe en cas d'abandon d'un des membres de cette équipe.
TRAITEMENTS / STRATEGIES / PROCEDURES	2 groupes randomisés A et B de 6 équipes chacun, composées elles-mêmes chacune de 4 personnes (1 médecin senior, 1 interne, 1 infirmier(ère), 1 ambulancier(e)) seront exposés à des prises en charge pluridisciplinaires simulées d'un nourrisson en état de choc (parmi une banque de scénarios prédéfinis) nécessitant la pose d'une VIO. Groupe A : 9 séances de simulation en 12 mois<br>Groupe B : 3 séances de simulation en 12 mois.
NOMBRE DE SUJETS	48
DUREE DE LA RECHERCHE	Durée de la période d'inclusion : 1 an<br>Durée de la participation pour chaque volontaire : 1 an<br>Durée totale de l'étude : 2 ans<br>Date de début théorique : mai 2013<br>Date de fin théorique : mai 2015
RETOMBEES ATTENDUES	Le stress diminue la performance d'équipe et la simulation répétée en climat de stress permet de maintenir une performance élevée.

1. INFORMATIONS GENERALES

1.1. Titre

Effets du stress et de la simulation sur la performance d'une équipe lors de la prise en charge d'un nourrisson en état de choc

1.2. Promoteur

1.2.1. Identité

Centre Hospitalier Universitaire de Poitiers
2 rue de la Milétrie-BP 577
86 021 Poitiers cedex
Tél : 05 49 44 46.65
Fax : 05 49 44 30 58

1.2.2. Signature du protocole au nom du promoteur

Jean-Pierre DEWITTE, Directeur Général
Centre Hospitalier Universitaire de Poitiers
2, Rue de la Milétrie - BP 577
86021 Poitiers cedex
Tél : 05 49 44 39 29
Fax : 05 49 44 39 80

1.2.3. Responsable de la recherche au niveau du promoteur

Le Directeur de la Recherche
Centre Hospitalier Universitaire de Poitiers
2 rue de la Milétrie-BP 577
86 021 Poitiers cedex
Tél : 05 49 44 46 65
Fax : 05 49 44 30 58

1.3. Coordination et suivi de l'étude

Dr Daniel Aiham GHAZALI
Centre Hospitalier Universitaire de Poitiers
Service de Urgences-SAMU86
2 rue de la Milétrie - BP 577 - 86021 Poitiers cedex

et un Attaché de Recherche Clinique désigné par le promoteur

1.4. Investigateurs

1.4.1. Investigateur coordonnateur

Dr Daniel Aiham GHAZALI
Centre Hospitalier Universitaire de Poitiers
Service de Urgences-SAMU86
2 rue de la Milétrie- BP 577 - 86021 Poitiers cedex
Tél : 05 49 45 43 87/ Fax : 05 49 44 39 01
E-mail : a.d.ghazali@chu-poitiers.fr

1.4.3. Investigateurs associés

Pr Denis ORIOT
Centre Hospitalier Universitaire de Poitiers
Urgences Pédiatriques
Service Médico-Chirurgical de Pédiatrie
2 rue de la Milétrie- BP 577 - 86021 Poitiers cedex
Tél : 05 49 44 61 71/ Fax : 05 49 44 37 59
E-mail : d.oriot@chu-poitiers.fr; denis.oriot@gmail.com

Pr Michel SCEPI
Centre Hospitalier Universitaire de Poitiers
Service de Urgences-SAMU86

2 rue de la Milétrie- BP 577 - 86021 Poitiers cedex
Tél : 05 49 44 40 88/ Fax : 05 49 44 39 01
E-mail : m.scepi@chu-poitiers.fr; michel.scepi@gmail.com

Dr Christine MILLET
Centre Hospitalier Universitaire de Poitiers
Laboratoire de Biophysique
2 rue de la Milétrie- BP 577 - 86021 Poitiers cedex
Tél : 05 49 44 49 59/ Fax : 05 49 44 40 58
E-mail : c.millet@chu-poitiers.fr

Dr Philippe SOSNER
Centre Hospitalier Universitaire de Poitiers
Service de Cardiologie
2 rue de la Milétrie- BP 577 - 86021 Poitiers cedex
Tél : 05 49 44 48 13/ Fax : 05 49 44 48 14
E-mail : philippe.sosner@chu-poitiers.fr

Dr Jean-Jacques CHAVAGNAT
Centre Hospitalier Henri Laborit
Pôle de santé publique et de logistique médicale
Pavillon Pierre Janet
370 avenue Jacques Cœur - 86021 poitiers cedex
Tél : 05 49 44 58 13/ Fax : 05 49 44 58 14
Jean-jacques.chavagnat@ch-poitiers.fr

1.5. Vigilance

Dr .Sophie DURANTON
Centre Hospitalier Universitaire de Poitiers
Direction de la Recherche Clinique
2 rue de la Milétrie - BP 577 - 86 021 Poitiers cedex
Tél : 05.49.44.30.50/ Fax : 05.49.44.30.58
E-mail : sophie.duranton@chu-poitiers.fr

1.6. Data-Management

Unité de Data-Management et Bibliométrie
Direction de la Recherche
Centre Hospitalier Universitaire de Poitiers
2 rue de la Milétrie - BP 577 - 86021 Poitiers cedex
Tel : 05.49.44.30.08/ Fax: 05.49.44.30.58
E-mail : farid.guetarni@chu-poitiers.fr

1.7. Méthodologiste – biostatisticien

Dr Stéphanie RAGOT
CIC – INSERM 0802
Centre Hospitalier Universitaire de Poitiers
2 rue de la Milétrie - BP 577 - 86021 Poitiers cedex
Tél : 05-49-44-46-89/ Fax : 05 49 44 46 91
E-mail : s.ragot@chu-poitiers.fr; stephanie.ragot@univ-poitiers.fr

2. JUSTIFICATION SCIENTIFIQUE ET DESCRIPTION GENERALE DE L'ETUDE

2.1- Dénomination et description de la situation clinique

Nous prendrons « l'état de choc chez un nourrisson » comme modèle de stress professionnel lors d'une prise en charge en équipe pluridisciplinaire.

2.2. Généralités

Un nourrisson de six mois, amené dans les bras de ses parents, a été pris en charge en salle d'accueil des urgences vitales (SAUV), pour un état de choc hypovolémique par déshydratation dans un contexte de gastro-entérite aiguë. L'équipe soignante, dont le leader était un jeune médecin, fut prise d'un immense stress devant l'impossibilité de mettre en place une voie veineuse périphérique, malgré de nombreuses tentatives. La mise en place d'une voie intra-osseuse (VIO), voie d'abord recommandée dans ce contexte (Biarent 2010), n'a pas été évoquée en l'absence de maîtrise de ce geste. Que faire ? Le pronostic vital de cet enfant n'a été épargné que grâce au renfort d'un médecin formé à la pratique de la VIO, permettant de faire une expansion volémique rapide et d'assurer le traitement du choc.

Comment garantir la sûreté d'un tel nourrisson en état de choc lors de sa prise en charge en SAUV ? Ce cas clinique reflète bien les trois éléments qui interviennent dans la sûreté (*safety*) d'un patient : la maîtrise des gestes techniques (taux de succès ou rendement – *efficiency*), tels la VIO (Oriot 1994), le respect des algorithmes (efficacité – *effectiveness*) (Biarent 2010) et la qualité (*quality*) du travail en équipe (Eppich 2008). La performance est la résultante de tous ces éléments pour maintenir la sûreté du patient.

La prise en charge d'un nourrisson en détresse vitale correspond à une situation clinique complexe où l'objectif est la réalisation et la coordination de nombreux gestes, par plusieurs acteurs, selon un algorithme préalablement établi par les recommandations internationales. La performance, c'est-à-dire la qualité globale de la prise en charge, dépend à la fois des actions menées en accord avec l'algorithme mais aussi de la qualité du travail en équipe pour coordonner la prise en charge du patient (Rosen 2008). Le stress, généré par le fait même qu'il s'agisse d'un nourrisson en détresse vitale, est un facteur pouvant modifier tous ces paramètres – maîtrise des gestes techniques, respect des algorithmes et travail en équipe – et donc la performance globale de la prise ne charge de l'enfant.

2.3 Le travail en équipe

Une des caractéristiques constituantes d'une équipe est que les membres de l'équipe doivent interagir les uns avec les autres afin de réaliser la tâche de l'équipe avec succès (Beaubien 2004). Le travail d'équipe devient un élément crucial pour un soin efficace au patient. Mais peu d'enseignements se sont penchés sur la formation au travail d'équipe (Shapiro 2008). Par un enseignement ciblé sur les équipes, il est possible d'établir une culture du travail en équipe dans un groupe de cliniciens, améliorant la sécurité des patients (Morey 2002). Le fonctionnement en équipe impose des compétences non techniques comme la répartition des tâches, le contrôle de la communication dans l'équipe, la conscience de la situation et la prise de décision (Yee 2005). Les recherches réalisées dans le domaine des compétences non techniques ont conduit au développement d'échelles d'évaluation du comportement et de la communication. Parmi les outils existants, l'échelle CTS (Clinical Teamwork Scale) a été utilisée et validée en situation d'urgence en simulation, avec une excellente reproductibilité inter-observateurs, attestée par un coefficient de corrélation intra-classe de 0,98 (Guise 2008).

Par ailleurs, un leadership d'équipe, fort et efficace, est absolument nécessaire pour atteindre la performance, car le leader a un triple rôle : développer l'esprit d'équipe, résoudre ses problèmes et apporter une motivation au travail en équipe. Les compétences requises pour être leader sont une grande perception de la dynamique d'équipe et une compréhension de l'impact des interactions personnelles et de la communication sur le fonctionnement d'une équipe (Shapiro 2008). L'enseignement du leadership est largement insuffisant et mérite d'être développé pour atteindre un degré d'excellence. L'évaluation du leadership est complexe ; en simulation, elle utilise le plus souvent l'échelle validée BAT (Behavioural Assessment Tool) (Anderson 2010).

2.4 Le stress

L'effet du stress de l'équipe soignante dans les prises en charge des patients doit être pris en compte, car il compromet la sûreté des patients (Gaba 2002). Le stress recouvre l'ensemble des perturbations biologiques, électro-physiologiques et psychiques provoquées par une agression quelconque sur un organisme. Il provoque une réponse émotionnelle – en particulier, l'anxiété aiguë – et une altération des processus cognitifs (Kirschbaum 1996, Wolf 2003). L'industrie aéronautique a reconnu depuis longtemps les effets du stress sur la performance, avec le risque de déficit en performance et d'erreurs humaines (Kohn 2000, Loewenthal 2000). Des pertes de performance ont aussi été rapportées dans les domaines militaire et sportif (Driskell 1991, Anshel 2002, Wallenius 2004). Le stress diminue la reconnaissance des erreurs par perte de la ligne attentionnelle et majore les réponses inadaptées au cours de prises en charge (Sexton 2000). Alors que la reconnaissance des erreurs et des facteurs de stress, associée à l'entraînement par simulation, améliorent la performance des équipes aéronautiques et augmente la sécurité des passagers (Sexton 2000).

Le stress a un important retentissement psychologique. L'état de stress post-traumatique (ESPT) est une pathologie fréquente en clinique psychiatrique et chez des personnes ayant subi un (ou plusieurs) événement(s) traumatique(s) au cours de leur vie (Yao 2003). Une des échelles d'appréciation de l'impact du stress post-traumatique à un mois est le *Post-traumatic Check List Scale* (PCLS), permettant une approche fiable de l'ESPT par auto-évaluation (Weathers 1993). Il existe d'autres échelles d'évaluation du stress validées en anglais, traduites et validées en français telle l'Échelle Révisée d'Impact de l'Événement (*Impact of Events Scale-Revised*, IES-R), très largement utilisée et reconnue au niveau international (Brunet 2003). Cette dernière a été créée par Weiss et Marmar en 1996, construite à partir de l'Échelle d'Impact de l'Événement d'Horowitz en 1979 (Horowitz 1979), et traduite et validée en français par Brunet en 2003 (Laurent 2007). L'IES-R mesure la présence de stress traumatique lié à des événements violents récents. La version initiale de l'Échelle d'Horowitz comprenait deux dimensions : les conduites d'évitement et de répétition associées à la symptomatologie traumatique. En 1996, Weiss et Marmar ont ajouté une troisième dimension : l'hyperactivité neurovégétative. Vingt-deux items évaluent ainsi l'intensité de chaque symptôme, selon une échelle de Likert d'« extrêmement » à « pas du tout » (Laurent 2007). Elle a été validée auprès de nombreuses victimes d'accidents ou d'agressions diverses. Elle différencie bien les sujets en état de stress aigu de ceux présentant un d'état de stress post-traumatique (Brunet 2003). Un score de 22 indiquerait un stress aigu et un score de 36 suggérerait la présence d'un ESPT, sans toutefois poser un diagnostic (Brunet 2003).

Le stress aigu des professionnels entraîne la mise en œuvre de différentes stratégies de gestion du stress (Anshel 2002). Il a été démontré qu'une mauvaise gestion du stress avait des effets négatifs, notamment sur la performance (Hassan 2006). Il a été récemment rapporté en simulation que la gestion du stress de chirurgiens était indépendante de l'expérience professionnelle (Wetzel 2010). Cette équipe a utilisé l'interview des acteurs après simulation afin de déterminer lesquelles des 6 stratégies définies ont été utilisées.

Le stress entraîne des modifications biologiques comme l'augmentation du taux de cortisol dans l'organisme. Tout comme le cortisol plasmatique, le cortisol salivaire est un reflet fidèle du stress avec l'avantage majeur de ne pas nécessiter de prélèvement invasif (Weibel 2003). Le cortisol salivaire est un bon marqueur biologique du stress aigu et a été dosé dans différents modèles de stress (Schreinicke 1990, Wagner 2010). Il est couramment utilisé pour suivre les athlètes ; il est plus élevé durant ou au décours d'une compétition que lors d'une séance d'entraînement quel que soit le niveau des sportifs (Maso 2002). Cette élévation du cortisol salivaire serait liée au stress physiologique et psychologique lors d'une compétition (Filaire 1997). De même, dans des modèles de stress durable, comme l'exposition aux nuisances sonores liées au trafic routier (Wagner 2010) ou le déplacement en avion (Berthon 2001), il a été rapporté une augmentation significative du taux de cortisol salivaire.

Le stress est également à l'origine de perturbations physiologiques comme l'hypertension artérielle, l'accélération de la fréquence cardiaque, des modifications de sa variabilité et des modifications du spectre de l'intervalle RR' de l'électrocardiogramme. Plusieurs variables peuvent être mesurées par Holter et traduire un stress : pression artérielle, fréquence cardiaque (FC) moyenne et sa variation, variation de l'écart-type de la FC moyenne, pourcentage d'intervalles RR' > 500 msec, Index de Variabilité (*Variability Index*, pourcentage de la différence moyenne entre deux RR' successifs), indice combiné comme le pNN50, l'ASDRR'/5 (mesure sur 5 minutes des RR' par la moyenne des écart-types des RR' par tranches de 5 min) et le SDARR'/5 (écart-types des moyennes des RR' par tranche de 5 min) (Anonymous 1996, Task Force 1996). L'analyse de l'intervalle RR' est une méthode non

invasive efficace pour évaluer le système nerveux autonome en condition de stress (Zheng 1997). Une corrélation a été établie entre l'état de stress et la diminution de l'intervalle RR' (Sloan 1994). L'effet global du stress, confirmé par augmentation du cortisol salivaire, est à l'origine d'une augmentation de la pression artérielle et d'une diminution de l'intervalle RR' (Lucini 2002). Ce marqueur a été utilisé pour apprécier l'effet du stress sur le risque cardiovasculaire de patients présentant une altération pathologique du système nerveux autonome, responsable d'une hypertension artérielle (Lucini 2005). Par ailleurs, l'intervalle RR' est également corrélé à l'effet du stress sur des personnes indemnes de toute pathologie cardiovasculaire, mais responsable d'une augmentation de la pression artérielle, comme les états de stress de la vie quotidienne (Lucini 2005), ou le stress per-anesthésique permettant de mesurer *a contrario* la profondeur de la sédation (Nishiyama 2010).

En médecine, l'effet du stress sur les professionnels est sous-estimé. Certains parlent même d'un déni des erreurs commises et de l'effet du stress sur la prise en charge du patient en comparaison avec le milieu de l'aviation (Sexton 2000). Certains auteurs ont pu mettre en évidence un état de stress post-traumatique chez les personnels du SAMU en France (Laurent 2007, Vaiva 2008), avec l'utilisation notamment de l'Echelle Révisée d'Impact de l'Evénement (IES-R) (Horowitz 1979, Brunet 2003, Craemer 2003). Très récemment, il a été démontré, en simulation, que le stress (mesuré par questionnaire, variabilité de la fréquence cardiaque et cortisol salivaire) altérait la performance chirurgicale et était un élément critique de la qualité des soins (Wetzel 2010). Cette équipe a utilisé un questionnaire d'auto-évaluation du stress analysant le fond anxieux et l'état anxieux déclenché par la situation. Il s'agit du State-Trait Anxiety Inventory (STAI) élaboré par Spielberger (Spielberger 1983).

2.5 La simulation

La connaissance médicale ne se borne pas à un savoir théorique, fut-il exhaustif, elle inclut aussi un savoir-faire pratique par la réalisation de gestes médico-techniques permettant d'acquérir une aptitude et de développer une compétence et un savoir-être relationnel vis-à-vis de l'équipe soignante, des patients et des familles. La simulation est un outil pédagogique permettant un tel apprentissage. La simulation médicale est systématisée dans les programmes pédagogiques de médecine d'urgence d'Amérique du Nord (Issenberg 1999, Issenberg 2008). En effet, en 2009, cette méthode d'apprentissage a même été reconnue par le Sénat Américain (*The Enhancing SIMULATION Act of 2009*), comme la méthode standard d'apprentissage des soins d'urgence (www.medsim.org, Forbes and Kennedy 2009). Pour atteindre un haut niveau de performance, l'aptitude (compétence) de chaque acteur doit être améliorée. La simulation permet d'atteindre cet objectif pédagogique par un apprentissage ex-vivo (en toute sécurité pour le patient et l'opérateur) et une évaluation formatrice de la qualité des différentes aptitudes pratiques acquises. Bien que la formation sur simulateur permette de réduire les erreurs, il est plus efficace d'associer à la formation sur simulateur une formation en équipe (Blum 2004, Birnbach 2008). La simulation permet d'améliorer le travail en équipe, notamment dans les services d'urgences adulte (Shapiro 2004) ou pédiatriques (Gilfoyle 2007, Eppich 2008).

2.6. Résumé des bénéfices, le cas échéant, et des risques prévisibles pour les personnes se prêtant à la recherche

2.6.1. Bénéfices

2.6.1.1 Bénéfice individuel

Le bénéfice pour un participant à la recherche est de recevoir gracieusement un enseignement par simulation – enseignement pratique innovant et évalué, sur les difficultés médicales et relationnelles rencontrées lors de prises en charge pédiatriques complexes.

2.6.1.2 Bénéfice collectif

Le bénéfice collectif est la formation par simulation de professionnels de santé du Centre Hospitalier Universitaire de Poitiers et des centres hospitaliers du Poitou-Charentes.

2.6.2 Risques

2.6.2.1 Risque individuel

2.6.2.1.1 Risques et contraintes physiques

Les risques liés à la participation sont l'inconfort causé par le port du Holter pendant 24 heures et la réalisation de trois prélèvements salivaires.

2.6.2.2. Risque collectif

Néant.

2.6.2.3 Préciser les EI et EIG attendus

Néant.

2.6.3. Balance bénéfices / risques

Cette étude offre une balance bénéfices/risques très positive.

2.7. Déclaration du respect du protocole, des bonnes pratiques cliniques et des dispositions législatives et réglementaires en vigueur dans le cadre de ce protocole

Le promoteur et l'investigateur s'engagent également à ce que cette recherche soit conduite :

- conformément au protocole,
- conformément aux bonnes pratiques cliniques françaises,
- conformément aux dispositions législatives et réglementaires actuellement en vigueur en France.

2.8. Description de la population à étudier

Nous étudierons des équipes multidisciplinaires composées de quatre membres : un médecin senior, un médecin junior (interne), un(e) infirmier(e) et un(e) ambulancier(e).

Pour chacun de ces statuts la liste exhaustive sera établie servant ainsi de base de sondage. Les participants tirés au sort seront contactés par courrier électronique pour présentation de l'étude et pour demande d'accord de participation (L'obtention de l'accord définitif, signé sera réalisé la veille de la première séance en présence du médecin coordination de l'étude). Les modalités du tirage au sort sont décrites paragraphe 4.5 .1

2.9. Références

1 Advanced Initiatives in medical simulation. Available at http://www.medsim.org/documents/H.R.855.pdf Accessed March 17, 2009
2. Anderson JDM, Meckler G, Boyle K, LeFlore J, Warre J. How to use the Behavioral Assessment Tool: assessment of CRM-based behavioral skills in simulation using a validated, reliable tool. Available at http://www.ipssw2010.com/img/IPSSW2010%20Programme%20final.pdf Accessed March 24, 2011
3. Anonymous. Heart rate variability. Standards of measurement, physiological interpretation, and clinical use. Task Force of the European Society of Cardiology and the North American Society of Pacing and Electrophysiology. Eur Heart J. 1996;17:354-81
4. Anshel MH, Anderson DI. Coping with acute stress in sport : Linking athletes' coping style, coping strategies, affect, and motor performance. Anxiety Stress Coping Int J 2002;15:193-209
5. Beaubien JM, Baker DP. The use of simulation for training teamwork skills health care: how low can you go? QualSaf Health Care 2004;13:i51-6
6. Beko G, Varga I, Glaz E, Sereg M, Feldman K, Toth M et al. Cutoff values of midnight salivary cortisol for the diagnosis of overt hypercortisolism are highly influenced by methods. Clinica Chimica Acta 2010;411:364-7
7. Berthon P, Lac G. Etude des réactions du cortisol salivaire au déplacement en avion pour une équipe de rugby. Science et Sports 2001;16:45-7
8. Biarent D, Bingham R, Eich C, López-Herce J, Maconochie I, Rodríguez-Núnez A et al. European Resuscitation Council Guidelines for Resuscitation 2010. Section 6. Paediatric life support. Resuscitation 2010;81:1364-88
9. Birnbach DJ, Salas E. Can medical simulation and team training reduce errors in labor and delivery? Anesthesiol Clin 2008;26:159-68
10. Blum RH, Reamer DB, Carroll JS, Sunder N, Felstein DM, Cooper JB. Crisis resource management training for an anaesthesia faculty: a new approach to continuing education. Med Educ 2004;38:45-55
11. Brunet A, Saint-Hilaire A, Jehel L, King S. Validation of a French version of the impact of Event Scale-Revised. Can J Psychiatry 2003;48:56-61

12. Creamer M, Bell R, Failla S. Psychometric properties of the Impact of Event Scale-Revised. Behav Res Ther 2003;41:1489-96
13. DASH. www.harvardmedsim.org/dash.html
14. Dovio A, Roveda E, Sciolla C, Montaruli A, Raffaelli A, Saba A et al. Intense physical exercise increases systemic 11beta-hydroxysteroid dehydrogenase type 1 activity in healthy adult subjects. Eur J Appl Physiol 2010;108:681-7
15. Driskell JE, Salas E. Overcoming the effects of stress on military performance: Human factors, training, and selection strategies. In: Gal R, Mangelsdorff D, eds, Handbook of military psychology, John Wiley and Sons Ed, Oxford 1991:183-93
16. Eppich WJ, Brannen M, Hunt FA. Team training: implications for emergency and critical care paediatrics. Curr Opin Pediatr 2008;20:255-60
17. Filaire E, Duché P, Robert A, Lac G. Influence d'une compétition officielle et d'une session d'entraînements sur les concentrations de cortisol salivaire. Science et Sports 1997;12:66-71
18. Forbes and Kennedy. HR 855 - A Bill to amend the Public Health Service Act to authorize medical simulation enhancement programs, and for other purposes. February 4, 2009
19. Gaba DM, Howard SK. Patient safety: fatigue among clinicians and the safety of patients. N Engl J Med 2002;347:1249-55
20. Gilfoyle E, Gottesman R, Razack S. Development of a leadership skills workshop in paediatric advanced resuscitation. Med Teach 2007;29:276-83
21. Guise JM, Deering SH, Kanki BG, Osterweil P, Li H, Mori M et al. Validation of a tool to measure and promote clinical teamwork. Simul Healthc 2008;3:217-23
22. Hassan I, Weyers P, Maschuw K et al. Negative stress-coping strategies among novices in surgery correlate with poor virtual laparoscopic performance. Br J Surg 2006;93:1554-9
23. Horowitz M, Wilner N, Alvarez W. Impact of Event Scale: a measure of subjective stress. Psychosom Med 1979;41:209-18
24. Issenberg SB, McGaghie WC, Hart IR, Mayer JW, Felner JM, Petrusa ER et al. Simulation technology for health care professionals skills training and assessment. JAMA 1999;282:861-6
25. Issenberg SB, Scalese RJ. Simulation in health care education. Perspect Biol Med 2008;51:31-46
26. Kleinman ME, Chameides L, Schexnayder SM, Samson RA, Hazinski MF, Atkins DL et al. American Heart Association Guidelines for Cardiopulmonary American Heart Association Guidelines for Cardiopulmonary 2010. Part 14: Pediatric Advanced Life Support. Circulation. 2010;122:S876-S908
27. Kirschbaum C, Wolf OT, May M et al. Stress and treatment-induced elevations of cortisol levels associated with impaired declarative memory in healthy adults. Life Sci1996;58:1475-83
28. Kohn LT, Corrigan J, Donaldson MS, Committee on Quality of health Care in America. To err is human: building a safer health system. National Academy Press Ed, Washington DC 2000
29. Laurent A, Chahraoui K, Carli P. Les répercussions psychologiques des interventions médicales urgentes sur le personnel SAMU. Etude portant sur 50 intervenants SAMU. Annales Médico Psychologiques 2007;165:570-8
30. Loewenthal KM, Eysenck M, Harris D et al. Stress, distress and air traffic incidents: Job dysfunction and distress in airline pilots in relation to contextually-assessed stress. Stress Med 2000;16:179-83
31. Lucini D, Norbiato G, Clerci M, Pagani M. Hemodynamic and autonomic adjustments to real life stress conditions in humans. Hypertension 2002;39:184-8
32. Lucini D, Di Fede G, Parati G, Pagani M. Impact of chronic psychosocial stress on autonomic cardiovascular regulation in otherwise healthy subjects. Hypertension 2005;46:1201-6
33. Maso F, Cazorla G, Godemet M, Michaux O, Lac G, Robert A. Influence d'une compétition de rugby sur le taux de cortisol salivaire. Science et Sports 2002;17:302-5
34. Morey JC, Simon R, Jay DG, Wears RL, Salisbury M, Dukes KA et al. Error reduction and performance improvement in the emergency department through formal teamwork training: evaluation results of the Med Teams project. Health Serv Res 2002;37:1553-81
35. Nishiyama T. Recent advance in patient monitoring. Korean J Anesthesiol 2010;59:144-59
36. Oriot D, Cardona J, Berthier M, Nasimi A, Boussemart T. La voie intra-osseuse : une voie d'abord vasculaire méconnue en France. Arch Pédiatr 1994;1:684-8
37. Oriot D, Darrieux E, Boureau-Voultoury A, Ragot S, Scépi M. Validation of a performance assessment scale for simulated intraosseous access. Sim Healthcare 2012;7:171-5
38. Rosen MA, Salas E, Wu TS, Silvestri S, Lazzara EH, Lyons R et al. Promoting teamwork: an event-based approach to simulation-based teamwork training for emergency medicine residents. Acad Emerg Med 2008;15:1190-8

39. Rudolph JW, Simon R, Dufresne RL, Raemer DB. There's no such thing as "non judgmental" debriefing: a theory and method for debriefing with good judgment. Simul Healthc 2006;1:49-55
40. Rudolph JW, Simon R, Raemer DB, Eppich WJ. Debriefing as formative assessment: closing performance gaps in medical education. Acad Emerg Med 2008;15:1010-6
41. Schreinicke G, Hinz A, Kratzsch J, Hüber B, Voigt G.Stress-related changes of saliva cortisol in VDU operators. Int Arch Occup Environ Health 1990;62:319-21
42. Sexton JB, Thomas EJ, Helmreich LR. Error, stress, and teamwork in medicine and aviation: cross sectional surveys. BMJ 2000;320:745-9
43. Shapiro MJ, Morey JC, Small SD, Langford V, Kaylor CJ, Jagminas L et al. Simulation based teamwork training for emergency department staff: does it improve clinical team performance when added to an existing didactic teamwork curriculum? Qual Saf Health Care 2004;13:417-21
44. Shapiro MJ, Gardner R, Godwin SA, Jay GD, Lindquist DG, Salisbury ML et al. Defining team performance for simulation-based training: methodology, metrics, and opportunities for emergency medicine. Acad Emerg Med 2008;15:1088-97
45. Sloan RP, Shapiro PA, Bagiella E, Boni SM, Paik M, Bigger JT Jr et al. Effect of mental stress throughout the day on cardiac autonomic control. Biol Psychol 1994;37:89-99
46. Spielberger CD. Manual for the Ste-Trait Anxiety Inventory (STAI). Consulting Psychologisyts Press, Palo Alto, CA, 1983
47. Task Force of the European Society of Cardiology and the North American Society of Pacing and Electrophysiology. Heart variability: standards of measurements, physiological interpretation and clinical use. Circulation 1996;93:1043-65
48. Vaiva G, Jehel L, Cottencin O, Ducrocq F, Duchet C, Omnes C et al. Prevalence of trauma-related disorders in the French WHO study. Encephale. 2008;34:577-83
49. Wagner J, Cik M, Marth E, Santner BI, Gallasch E, Lackner A et al. Feasibility of testing three salivary stress biomarkers in relation to naturalistic traffic noise exposure. Int J Hyg Environ Health 2010;213:153-5
50. Wallenius C. Military observers' reactions and performance when facing danger. Mil Psychol2004;16:211
51. Weathers FW, Litz BT, Herman DS, Huska JA, Keane TM. The PTSD Checklist (PCLS): Reliability, validity, and diagnostic utility. 9th Annual Conference of the ISTSS, San Antonio, Texas, USA, 1993.
52. Weibel L, Gabrion I, Aussedat M, Kreutz G. Work-related stress in an emergency medical dispatch center. Ann Emerg Med 2003;41:500-6
53. Wetzel CM, Black SA, Hanna GB, Athanasiou T, Kneebone RL, Nestel D et al. The effects of stress and coping on surgical performance during simulations. Ann Surg 2010;251:171-6
54. Wilhelm FH, Grossman P, Roth WT. Assessment of heart rate variability during alterations in stress: complex demodulation vs. spectral analysis. Biomed SciInstrum 2005;41:346-51
55. Wolf OT. HPA axis and memory. Best Pract Res Clin Endocrinol Metab 2003 ;17 :287-99
56. Yao SN, Cottraux J, Note I, De Mey-Guillard C, Mollard E, Ventureyra V. Evaluation de l'état de stress post-traumatique : validation d'une échelle, la PCL-S. Encephale 2003;29:232-8
57. Yee B, Naik VN, Joo HS, Savoldelli GL, Chung DY, Houston PL et al. Non-technical skills in anesthesia crisis management with repeated exposure to simulation-based education. Anesthesiology 2005;103:241-8
58. Zheng D, Shen L, Wu G, Jiang Y, Zhou Y, Wang W. Spectral analysis of R-R intervals in adolescent persons during mental stress. J Biomed Engin 1997;14:38-41

3. OBJECTIFS DE L'ETUDE

3.1. Objectif principal

Evaluer ***l'effet du stress sur la performance*** d'une équipe, avec trois volets :

- o La performance globale de l'équipe : respect de l'algorithme de prise en charge et réalisation des gestes indiqués.
- o La VIO : technique de pose et succès.
- o Le travail en équipe : leadership et communication.

3.2. Objectifs secondaires

- ***Evaluer l'effet de la simulation répétée sur l'évolution de la performance*** d'une équipe dans le maintien de la sûreté du nourrisson en état de choc, avec trois volets :
    - o La performance globale de l'équipe : respect de l'algorithme de prise en charge et réalisation des gestes indiqués.
    - o La VIO : technique de pose et succès.
    - o Le travail en équipe : leadership et communication.
- ***Evaluer l'effet de la simulation répétée sur le stress*** d'une équipe dans le maintien de la sûreté du nourrisson en état de choc, avec trois évaluations du stress :
    - o Le cortisol salivaire
    - o Le Holter : variabilité de la fréquence cardiaque, intervalle RR' et autres paramètres
    - o Les questionnaires de stress STAI, IES-R et PCLS

4. CONCEPTION DE L'ETUDE

4.1. Critère d'évaluation principal

Notre critère d'évaluation principal est la **performance de l'équipe** dans la prise en charge simulée d'un nourrisson en état de choc. Cela sera évalué de trois façons :

- La performance globale d'une équipe par le score obtenu sur l'échelle globale de performance en cours de validation (Oriot 2013) (cf. Annexe).
- La performance de la pose d'une VIO : par l'échelle d'évaluation IOPAS – *Intraosseous Performance Assessment Scale* (Oriot 2012), délais décisionnels et de mise en place (cf. Annexe).
- La performance du travail en équipe :
    - Le leadership par l'échelle BAT – *Behavioural Assessment Tool* (Anderson 2010) (cf. Annexe).
    - L'évaluation globale du travail en équipe par l'échelle CTS - *Clinical Teamwork Scale*, CTS (Guise 2008)] (cf. Annexe).

4.2. Critères d'évaluation secondaires

4.2.1 Evaluation du stress

Le stress qui sera évalué de 3 façons différentes :

- Biologique : dosage du cortisol salivaire par technique de Radio-Immuno-Essai (Radio-Immuno-Assay, RIA). Cette technique est validée et utilisée pour l'évaluation de l'effet du stress (Wagner 2010, Beko 2010, Dovio 2010). Ce dosage sera réalisé la veille, avant et après simulation.
- Electrophysiologique :
    - mesure de la variation de la fréquence cardiaque, de sa variabilité, du pNN50 et du spectre de l'intervalle R-R' (Wilhelm 2005) de chaque participant par enregistrement Holter, avant (pendant environ 21h), pendant et après simulation (pendant 3 heures) (Task Force 1996). Acquisition de 4 appareils Holter (Sorin Group*).
    - Mesure de la pression artérielle (PA) la veille, juste avant et après simulation, après le débriefing. Cette mesure sera couplée à une mesure ponctuelle de la fréquence cardiaque (FC). Les mesures de PA et FC seront obtenues par le même appareil. Ces mesures ont pour but d'étudier le système nerveux autonome sous l'effet du stress.

- Psychologique :
  - Mesure par le questionnaire d'auto-évaluation STAI – *State-Trait Anxiety Inventory* (Spielberger 1983) de l'état anxieux la veille, juste avant et après simulation, après le débriefing (cf. Annexe). Nous avons choisi de travailler sur une version réduite validée plutôt que la version standardisée officielle. En effet, devant la multiplicité des mesures et du temps dédié hors simulation pour les participants, nous avons décidé d'utiliser cette version moins chronophage. Par ailleurs, cette version réduite couplée aux échelles de mesures du stress post-évènementiel est suffisante pour étudier le stress psychologique.
  - Mesure du stress post-événementiel précoce par l'échelle de stress IES-R – *Impact of Event Scale-Revised*, traduite et validée en français (Brunet 2003), 7 jours après la séance de simulation
  - Mesure du stress post-événementiel durable par l'échelle PCLS – *Posttraumatic Check-List Scale* (Weathers 1993), un mois après la séance de simulation.

Enfin, les stratégies de gestion du stress par le médecin sénior seront déduites des évaluations précédentes et appréciées de 2 façons différentes en s'inspirant des axes précisés par Wetzel (Wetzel 2010) :

- L'observation pendant la simulation
- La relecture des vidéos par les superviseurs

4.2.2 Evaluation de l'évolution de la performance

Pour étudier l'évolution nous calculerons la variation relative des différents variables scores de performance. La variation relative sera définie par le calcul (score final – score T0)/score T0

4.3. Description des interventions

- Formation initiale des médecins seniors

Tous les médecins seniors auront eu, préalablement à la recherche, une formation identique sur la mise en place d'une VIO chez le nourrisson (cours théorique et pratique sur task-trainer) avec évaluation théorique et pratique avec l'échelle d'évaluation mise au point au Laboratoire de Simulation (Oriot 2012) lors du Diplôme Universitaire des Gestes d'Urgence en Pédiatrie (Laboratoire de Simulation, Faculté de Médecine de Poitiers, Pr Oriot).

- Mannequin et scénarios

Nous utiliserons comme mannequin haute-fidélité le SimNewB* (Laerdal*) du Laboratoire de Simulation de Poitiers et notre banque de scénarios de simulation d'état de choc chez le nourrisson (chaque scénario sera tiré au sort parmi les cas restants) qui amèneront les équipes à prendre en charge en SAUV simulée un nourrisson en état de choc avec nécessité de pose d'une VIO. Nous utiliserons neuf scénarios d'état de choc chez l'enfant tirés au sort parmi les 18 suivants : choc hypovolémique (3e secteur digestif, déshydratation aiguë sur gastroentérite, brûlures étendues, acidocétose diabétique, diabète insipide, cerebral salt wasting post-traumatique, insuffisance surrénale par hyperplasie congénitale des surrénales), choc hémorragique (hématome sous-galéal, hémoptysie fulminante par hypertension artérielle pulmonaire, traumatisme sévère avec hémorragie d'origine rétro-péritonéale, traumatisme sévère avec hémorragie d'origine péritonéale et fémorale), choc anaphylactique d'origine médicamenteuse et alimentaire, choc cardiogénique (myocardiopathie virale, tachycardie supra-ventriculaire), choc septique (purpura fulminans), choc par anémie hémolytique (paludisme avec hémolyse aiguë), arrêt cardio-respiratoire compliquant un état de choc décompensé.

- Stress

Le stress sera celui qui est lié aux scénarios eux-mêmes (accident hypoxique, décompensation hémodynamique, survenue de convulsions), à l'environnement rendant l'ambiance stressante (bruit du scope et des alarmes) et l'issue intempestive des parents simulés dans le déchocage si cela est prévu dans le scénario.

- Superviseurs et évaluation

Deux superviseurs indépendants évalueront toutes les séances de simulation du protocole de recherche Sim-Stress. Ces superviseurs, font partie du laboratoire de simulation du CHU de Poitiers, sont choisis de manière aléatoire. Ils ont chacun une spécialité différente et sont tous formateurs en simulation, habilités à réaliser des débriefings.
Cette équipe de superviseurs est composée du Pr Denis Oriot (réanimateur pédiatrique, Amélie Boureau (urgentiste pédiatrique), Franck Petitpas (réanimateur chirurgical) et Youcef Guechi (urgentiste adulte). Ils analyseront la performance globale avec le respect de l'algorithme, la pose de la VIO et le travail en équipe – leadership et communication. La moyenne entre les deux scores des superviseurs sera utilisée comme valeur de référence. La reproductibilité inter-observateur de l'échelle utilisée sera calculée.

- Vidéo

Toutes les séances de simulation seront filmées, de façon à pouvoir relire les vidéos et analyser les actions et les interventions réalisées pendant le scénario (évaluation de la compétence globale et des stratégies utilisées pour gérer le stress).

De plus, les vidéos seront revues par deux autres observateurs indépendants ne connaissant pas les intervenants pour évaluer le travail en équipe. La moyenne entre les deux scores des superviseurs sera utilisée comme valeur de référence. La reproductibilité inter-observateurs de l'échelle d'évaluation du travail en équipe (leadership et communication) sera calculée. Parallèlement seront évaluées les stratégies mises en œuvre par le médecin senior pour la gestion du stress. Une comparaison sera réalisée avec les superviseurs observant *de visu* les séances de simulation.

- Débriefing

Toutes les séances de simulation seront suivies d'un débriefing, mené selon la méthode du « *good judgment* » de Rudolph (Rudolph 2006, Rudolph 2008) par des superviseurs préalablement formés à cette technique (Formation Universitaire à l'Université de Paris Descartes).

- Répétition des séances de simulation

La fréquence des répétitions des séances de simulation sera un des éléments variant et définira l'appartenance à l'un des deux groupes randomisés, nécessaire pour répondre aux objectifs secondaires. Elle sera soit toutes les 6 semaines (9/an), soit tous les 6 mois (3/an).

4.4. Déroulement de l'étude

4.4.1 <u>Schéma général de recherche lors d'une séance de simulation en équipe</u>

Variables		Veille	Pré-sim	Sim	Post-sim	Débrief	Post-débrief	H+2	1 Sem	1 mois
**PERFORMANCE**	**Performance globale**			X						
	**VIO**			X						
	**Leadership (BAT)**			X						
	**Travail en équipe (CTS)**			X						
**STRESS**	**Cortisol salivaire**	X	X		X		X			
	**Holter – FC, pNN50**	X	X	X	X	X	X	X		
	**PA et FC**	X	X		X		X			
	**Anxiété (STAI)**	X	X		X		X			
	**Stress post-événementiel (EIS-R) à J7**								X	
	**Stress post-événementiel (PCLS) à 1 mois**									X

Ce plan sera répété à l'occasion de chaque séance de simulation, c'est-à-dire toutes les 6 semaines (pour les équipes du groupe A) ou tous les 6 mois (pour les équipes du groupe B).

4.4.2 <u>Quantification des séances et des différentes évaluations</u>

Nombre de participants inclus dans la recherche

- 12 équipes de 4 personnes = **48 participants**

Nombre de séances de simulation en équipe :

- Participants du groupe A : 6 équipes x 9 séances = 54 séances de simulation
- Participants du groupe B : 6 équipes x 3 séances = 18 séances de simulation
- TOTAL = 54 + 18 = **72 séances de simulation en équipe**

Nombre de séances individuelles de simulation :

- Groupe A : 6 équipes x 4 x 9 séances = 216
- Groupe B : 6 équipes x 4 x 3 séances = 72
- TOTAL = 216 + 72 = **288 séances individuelles de simulation**

Nombre d'enregistrement Holter

- Groupe A : 9 enregistrements holter par participant
- Groupe B : 3 enregistrements holter par participant
- Total : 1 enregistrement holter par séance soit **288 enregistrements Holter**

Nombre de mesures de pression artérielle et de fréquence cardiaque

Les mesures seront faites la veille de la séance puis en pré et post simulation ainsi qu'en post débriefing, soit 4 fois par séance

- Groupe A : 36 mesures par participant
- Groupe B : 12 mesures par participant

- Total : 288 x 4 = **1152 mesures de pression artérielle et fréquence cardiaque**

Nombre de dosages du cortisol salivaire

Le cortisol salivaire sera dosé la veille de la séance puis en pré et post simulation ainsi qu'en post débriefing, soit 4 fois par séance

- Groupe A : 36 dosages du cortisol par participant
- Groupe B : 12 dosages du cortisol par participant
- Total : 288 x 4 = **1152 dosages du cortisol**

4.5. Description des mesures prises pour réduire et éviter les biais

4.5.1 Tirage au sort

- Tirage au sort pour constitution d'échantillons représentatifs

Les listes des personnes correspondant à chacun des 4 statuts professionnels précédemment définis seront établies et serviront de base de sondage pour effectuer un tirage au sort dans chacune des catégories. Ce tirage au sort permettra de constituer des échantillons représentatifs pour chacun des statuts professionnels, permettant ainsi d'éviter un biais de sélection. La taille de ces échantillons sera le double de la taille nécessaire, soit 24 personnes, afin de tenir compte des refus éventuels de participation. Les personnes sélectionnées par le tirage au sort seront contactées dans l'ordre de la liste de tirage au sort jusqu'à obtenir 12 personnes dans chaque groupe donnant leur accord de participation.

Les équipes comprenant un représentant de chacun des statuts (soit 4 personnes) seront constituées en suivant l'ordre de la liste de tirage au sort (équipe 1 = 1er personne de la liste de TAS à condition qu'elle ait donné son accord de participation et que tous les critères d'inclusion/ non inclusion soient respectés dans chacun des 4 groupes professionnels). 12 équipes seront ainsi constituées.

- Tirage au sort pour répartition aléatoire des 2 programmes de simulation entre les équipes.

Une fois les équipes constituées, un tirage au sort sera réalisé selon une liste établie par le méthodologiste de l'étude afin de répartir les 12 équipes en 2 groupes de 6 équipes :

Le groupe A réunira 6 équipes de 4 personnes et chacune de ces équipes aura une séance de simulation toutes les ***6 semaines***, soit 9 séances en 12 mois.
Le groupe B réunira 6 équipes de 4 personnes et chacune de ces équipes aura une séance de simulation tous les ***6 mois***, soit 3 séances en 12 mois.

4.5.2 Homogénéité de la population d'étude

Nous avons établis des critères d'inclusion relativement étroits afin d'homogénéiser la formation et/ou l'expérience professionnelle des participants pour chacun des statuts.

4.5.3 Méthodes d'évaluation par échelles d'hétéro-évaluation

Toutes les évaluations faisant appel à des hétéro-évaluations seront faites par deux superviseurs indépendant l'un de l'autre et en aveugle des groupes d'affectation ainsi que de la direction des scénarios. Le calcul des coefficients de corrélation intra-classe sera réalisé pour chacune des échelles d'évaluation utilisée, même si celles-ci sont déjà validées, afin de mesurer la reproductibilité inter-observateur.

4.6. Durée de l'étude

Durée totale de l'étude estimée à : **24 mois**
Durée totale de participation à l'étude pour le participant : **12 mois**

Dès la première inclusion, le promoteur doit informer sans délai l'autorité compétente et le CPP de la date effective de démarrage de l'étude (Date effective de démarrage = date de signature du consentement par la première personne qui se prête à la recherche).
La date de fin d'étude sera transmise par le promoteur à l'ANSM et au CPP dans un délai de 90 jours.
La date de fin de la recherche correspond au terme de la participation de la dernière personne qui se prête à la recherche, ou le cas échéant, **au terme défini dans le protocole.**

4.7. Description des règles d'arrêt définitif ou temporaire

4.7.1. Arrêt de participation d'un participant dans cette étude

Les participants pourront retirer leur consentement et demander à sortir de l'étude à n'importe quel moment et quelle qu'en soit la raison. En cas de sortie prématurée, l'investigateur doit en documenter les raisons de façon aussi complète que possible.
L'investigateur pourra interrompre temporairement ou définitivement la participation d'un participant à l'étude pour toute raison qui servirait au mieux les intérêts du sujet en particulier en cas d'événements indésirables graves.

4.7.2. Arrêt d'une partie ou de la totalité de la recherche

L'étude peut être interrompue prématurément en cas de survenue d'événements indésirables inattendus, graves nécessitant une revue du profil de la stratégie.
De même, des événements imprévus au vu desquels les objectifs de l'étude ne seront vraisemblablement pas atteints, peuvent amener le promoteur à interrompre prématurément l'étude.
Le CHU de POITIERS se réserve le droit d'interrompre l'étude, à tout moment, s'il s'avère que les objectifs d'inclusion ne sont pas atteints.
En cas d'arrêt prématuré de l'étude, l'information sera transmise par le promoteur dans un délai de 15 jours au CPP.

4.8. Identification de toutes les données à recueillir directement dans les cahiers d'observation, qui seront considérées comme des données sources

Un CRF papier sera mis à la disposition des investigateurs qui reporteront directement les données cliniques des participants inclus.
Les données recueillies le CRF pourront ainsi être considérées comme des données sources. L'investigateur devra dater et signer cet exemplaire a la fin du recueil des données.

5. CRITERES D'INCLUSION ET DE NON INCLUSION

5.1. Critères d'inclusion

- La participation à la recherche se fera sur la base du volontariat.
- Age ≥ 18 ans.
- Sujet libre, sans tutelle ou curatelle ni subordination.
- Participants bénéficiant d'un régime de Sécurité Sociale.
- Consentement libre et éclairé.
- Accord écrit pour l'évaluation par vidéo à des fins exclusives de recherche.
- Equipe de quatre personnes :

  1/- Médecin urgentiste de la région Poitou-Charentes et des alentours ayant une expérience professionnelle de moins de sept ans et ayant obtenu le Diplôme Universitaire des Gestes d'Urgence en Pédiatrie dans les 3 dernières années, faisant suite aux dernières recommandations de réanimation avancée pédiatrique établies en 2010 par l'AHA (American Heart Association) (Kleinman 2010) et l'ERC (European Resuscitation Council) (Barient 2010);

  2/- Interne en médecine inscrit au DESC de Médecine d'Urgence à l'Université de Poitiers, formés aux gestes d'urgence en pédiatrie. Ils ont validés un stage clinique dans un service des urgences pédiatriques ou le Diplôme Universitaire des Gestes d'Urgence en Pédiatrie;

  3/- Infirmier(e) du SAMU-SMUR du CHU de Poitiers ayant une expérience professionnelle de moins de sept ans et ayant obtenu le diplôme EPILS – European Pediatric Immediate Life Support – lors des deux dernières années ;

  4/- Ambulancier(e) du SAMU-SMUR du CHU de Poitiers ayant une expérience professionnelle de moins de sept ans.

5.2. Critères de non inclusion

- Age < 18 ans
- Participation actuelle à une autre étude de recherche clinique avec une période d'exclusion d'un mois entre les deux protocoles de recherche
- Participants ne bénéficiant pas d'un régime de Sécurité Sociale
- Femmes enceintes ou allaitantes, femmes en âge de procréer ne disposant pas d'une contraception efficace (hormonal/mécanique : per os, injectable, transcutané, implantable, dispositif intra-utérin, ou bien chirurgical : ligature des trompes, hystérectomie, ovariectomie totale), femmes ménopausées.
- Antécédents médicaux (Infection sévère, maladie métabolique avec modification des réponses neuroendocriniennes au stress, maladie grave type infarctus à l'origine d'un stress pathologique) et/ou psychiatriques pouvant influencer l'état de stress (syndrome anxio-dépressif, anxiété généralisée, dépression quel qu'en soit le stade, troubles psychotiques).
- Antécédents cardiaques ou neurologiques convulsivants.
- Port de pacemaker ou de défibrillateur implantable.
- Participant prenant des médicaments influençant le stress : cardiotropes et/ou des bronchodilatateurs β2-mimétiques (modification possible des paramètres électrophysiologiques cardiaques), corticoïdes et hormonothérapie substitutive (modification des paramètres biologiques de stress), traitement psychotrope (modification des paramètres psychologiques du stress).

Critères de sortie de l'étude

- Non-respect des dates et horaires d'évaluation pouvant compromettre les séances de simulation ou influencer les paramètres étudiés.
- Exclusion de toute une équipe en cas d'abandon d'un des membres de cette équipe.

5.3. Modalités de recrutement

Selon le statut des participants, le mode de recrutement diffèrera :

- 12 Médecins seniors (urgentistes) de la région Poitou-Charentes et des alentours ayant tous reçu le même enseignement théorique et pratique sur la prise en charge d'un nourrisson en état de choc et sur la pose d'une VIO, du fait de leur validation du Diplôme Universitaire des Gestes d'Urgence en Pédiatrie lors des trois dernières années.
- 12 Internes en médecine inscrits au DESC de Médecine d'Urgence de l'Université de Poitiers et en stage dans les hôpitaux du Poitou-Charentes. Ils sont formés à la prise en charge d'un nourrisson en état de choc et sur la pose de VIO.
- 12 Infirmier(e)s du CHU de Poitiers (Urgences Adultes – SAMU/SMUR).
- 12 Ambulancier(e)s du CHU de Poitiers (SAMU/SMUR).

Pour chacun de ces statuts la liste exhaustive sera établie servant ainsi de base de sondage. Les participants tirés au sort seront contactés par courrier électronique pour présentation de l'étude et pour demande d'accord de participation (L'obtention de l'accord définitif, signé sera réalisé la veille de la première séance en présence du médecin coordination de l'étude).
Les modalités du tirage au sort sont décrites paragraphe 4.5 .1

5.4. Procédure d'exclusion de la recherche

5.4.1. Critères et modalités d'exclusion d'une personne de la recherche

Les sujets ayant retiré leur consentement et demandé à sortir de l'étude seront exclus.
L'investigateur pourra interrompre la participation d'un sujet à l'étude pour toute raison qui servirait au mieux les intérêts du sujet en particulier en cas d'événements indésirables graves.
Un participant sera exclu de la recherche en cas de non-respect des dates et horaires d'évaluation pouvant compromettre les séances de simulation ou influencer les paramètres étudiés.
L'exclusion d'un membre d'une équipe résultera en l'exclusion de toute l'équipe et la nécessité d'inclure à nouveau une équipe au complet.

5.4.2. Modalités et calendrier de recueil pour ces données

Les données des participants ayant retiré leur consentement seront analysées uniquement si les participants ont donné un avis favorable.

5.4.3. Modalités de suivi de ces personnes

La sortie d'étude d'un participant ne changera en rien les relations professionnelles vis-à-vis de cette personne.
En cas d'événement indésirable, grave ou non, un suivi précis pourra être envisagé en fonction de la gravité de l'événement indésirable et de la sévérité.

6. ANALYSES

L'analyse portera sur plusieurs questions de recherche :

6.1 Evaluation de l'effet du stress sur la performance

Dans un premier temps, nous évaluerons l'effet du ***stress*** sur la ***performance*** d'une équipe, avec trois volets :

- Attester de l'existence d'un stress biologique, électrophysiologique et psychologique lors du déroulement des différents scénarios.
- Evaluer l'impact du stress sur la performance globale de l'équipe (respect de l'algorithme).
- Evaluer l'impact du stress sur la pose d'une VIO : technique de pose (échelle d'évaluation), délai décisionnel et de réalisation.
- Evaluer l'impact du stress sur le travail en équipe : évaluation du leadership (échelle d'évaluation) et communication (échelle d'évaluation).

6.2 Evaluation de l'effet de la répétition des séances de simulation

Dans un second temps, nous évaluerons l'effet de la ***simulation répétée*** sur ***l'évolution de la performance*** d'une équipe dans le maintien de la sûreté d'un nourrisson en état de choc, avec trois volets :

- Evaluer l'impact de la répétition des séances de simulation sur la performance globale : respect de l'algorithme.
- Evaluer l'impact de la répétition des séances de simulation sur la pose d'une VIO : technique de pose (échelle d'évaluation), délai décisionnel et de réalisation.
- Evaluer l'impact de la répétition des séances de simulation sur le travail en équipe : évaluation du leadership (échelle d'évaluation) et communication (échelle d'évaluation).

Enfin, nous étudierons l'effet de la simulation répétée sur le stress en évaluant les différents marqueurs du stress (biologique, électrophysiologique et psychologique) et l'impact de la répétition des séances de simulation sur le développement des stratégies de gestion du stress.
Nous pourrons ainsi savoir si la répétition des séances de simulation, attendue pour améliorer la performance, se fait avec le maintien d'un niveau de stress élevé avec mise en place de stratégies de gestion du stress, ou bien avec une diminution du niveau de stress parallèle à l'augmentation de la répétition des séances de simulation.
L'évaluation de l'effet de la stimulation répétée se fera par comparaison des 2 groupes : les équipes recevant 9 séances de simulation versus les équipes bénéficiant seulement de 3 séances comme décrit au paragraphe 9

7. EVALUATION DE LA PERFORMANCE ET DU STRESS

7.1. Description des paramètres d'évaluation de la performance

Nos critères de jugement principaux sont ***les échelles d'évaluation concernant la performance globale de l'équipe, celle de la pose de la VIO et celle du travail en équipe***.
Ils sont décrits décrits dans le paragraphe 4.1

Par ailleurs, nous utiliserons une double évaluation (observation pendant la simulation et relecture des vidéos) afin d'analyser les stratégies de gestion du stress mises en œuvre par le médecin senior au cours des séances de simulation.

7.2. Description des paramètres d'évaluation du stress

Nos critères de jugement secondaires sont les ***marqueurs du stress*** évalués chez chaque participant. Ils sont décrits dans le paragraphe 4.2.1

7.3. Méthodes et calendrier prévus pour mesurer, recueillir et analyser les paramètres d'évaluation de la performance et du stress

7.3.1 Mesure de la performance

La mesure de la performance fera appel à une hétéro-évaluation réalisée à l'aide de plusieurs échelles d'évaluation (évaluation de la performance globale, évaluation de la pose d'une VIO, évaluation du leadership, évaluation du travail d'équipe), par deux superviseurs indépendants pendant la séance de simulation.
La relecture des vidéos leur permettra secondairement de déterminer les stratégies de gestion du stress mises en œuvre par le leader de l'équipe.

7.3.2 Mesure du stress

La mesure du stress fera appel à différents outils qui mériteront un traitement particulier :

- Le cortisol salivaire sera prélevé la veille de la séance de simulation (vers 18h), le jour de la séance de simulation, juste avant et juste après la séance (avant le débriefing) et après le débriefing. Les tubes seront conservés au réfrigérateur prévu à cet effet au Laboratoire de Simulation, puis adressé au laboratoire de Biochimie du CHU de Poitiers pour analyse (Dr Christine Millet et Dr Aiham Ghazali).
- Le Holter sera posé sur les participants par le Dr A. Ghazali pour les hommes et par Emmanuelle Fournier pour les femmes, la veille de la simulation (vers 18h) et restera porté pour une durée de 24 heures au total. La séance de simulation aura lieu de 14 à 15h au Laboratoire de Simulation, ce qui fait que le Holter restera en place 3 heures après la fin de la séance. Les cassettes seront retirées, collectées et lues une fois par semaine dans le service de Cardiologie du CHU de Poitiers (Dr Philippe Sosner et Dr Aiham Ghazali).
- Une mesure non invasive de de la pression artérielle sera effectuée la veille de la séance de simulation (vers 18h), le jour de la séance de simulation, juste avant et juste après la séance (avant le débriefing) et après le débriefing. Chaque mesure de pression artérielle est associée à une mesure ponctuelle de la fréquence cardiaque.
- La mesure du stress psychologique fera appel à trois questionnaires :
    - Le STAI est un auto-questionnaire évaluant l'état anxieux de base et le stress aigu provoqué que les participants rempliront la veille de la simulation et le jour de la simulation, avant la séance et juste après la séance (avant le débriefing).
    - L'EIS-R est un questionnaire permettant d'évaluer le stress post-événementiel (ici, l'événement est la séance de simulation) de façon proche, à 7 jours. Il sera envoyé aux participants par courrier électronique. Les réponses seront obtenues par retour de mail et recueillies par un assistant de recherche.
    - Le PCLS est un questionnaire permettant d'évaluer le stress post-événementiel (ici, l'événement est la séance de simulation) de façon différée, à un mois Il sera envoyé aux participants par courrier électronique. Les réponses seront obtenues par retour de mail et recueillies par un assistant de recherche.

8. EVALUATION DE LA SECURITE

8.1. Paramètres d'évaluation de la sécurité

La tolérance clinique au cours de la recherche sera objectivée par l'analyse des évènements indésirables et/ou intercurrents survenant au cours de la recherche et qui devront être systématiquement reportés sur le cahier d'observation.

8.2. Méthodes et calendrier prévus pour mesurer, recueillir et analyser les paramètres d'évaluation de la sécurité

Les événements indésirables seront évalués à chaque séance au cours de son déroulement.
Il est demandé à chaque investigateur de noter : la gravité de l'événement, le type d'événement, l'intensité, la relation avec la séance de simulation (improbable, probable, non évaluable), la date de survenue, la durée, le traitement entrepris.

8.3. Procédures mises en place en vue de l'enregistrement et de la notification des événements indésirables

8.3.1. Définitions

- **Evénement indésirable (EvI)** : toute manifestation nocive survenant chez une personne qui se prête à une recherche biomédicale que cette manifestation soit liée ou non à la recherche ou aux procédures expérimentales sur lesquelles porte cette recherche.
- **Evénement indésirable grave (EvIG)** : la gravité est définie par l'une des constatations suivantes :
    - décès
    - mise en jeu du pronostic vital (menace vitale immédiate, au moment de l'événement, et ce, indépendamment des conséquences qu'aurait une thérapeutique correctrice ou palliative)
    - incapacité ou handicap important ou durable
    - hospitalisation
    - événement potentiellement grave (événement clinique indésirable ou résultat de laboratoire à caractère grave ou considéré comme tel par l'investigateur)
- **Effet indésirable (EI)** : toute réaction nocive et non désirée liée à la stratégie
- **Effet indésirable grave (EIG)** : évènement indésirable grave imputable à la stratégie.
- **Effet indésirable inattendu** : effet indésirable dont la nature, la sévérité, l'intensité ou l'évolution ne concorde pas avec les informations figurant dans la brochure de l'investigateur.
- **Imputabilité :** relation entre l'EvI et la stratégie de l'étude. L'EvI lié à la stratégie deviendra un EI. Les facteurs à prendre en compte pour la détermination de l'imputabilité sont :
    - la chronologie des évènements,
    - la disparition de l'EvI lors de l'arrêt de la stratégie et/ou la réapparition en cas de ré-intervention,
    - la notion d'antécédent d'évènement similaire lors de la mise en place de la stratégie,
    - l'existence d'une autre étiologie.
    - **Intensité** : l'intensité des EvI est évaluée par l'investigateur, selon la classification suivante :
    - léger de grade 1 : EvI généralement transitoire et sans retentissement sur les activités normales,
    - modéré de grade 2 : EvI suffisamment gênant pour retentir sur les activités normales,
    - sévère de grade 3 : EvI modifiant considérablement le cours normal des activités du participant, ou invalidant, ou constituant une menace pour la vie du participant.

Remarque : le critère d'intensité ne doit pas être confondu avec le critère de gravité qui sert de guide pour définir les obligations de déclaration

8.3.2. Rôle de l'investigateur

8.3.2.1. Notification des événements indésirables graves

8.3.2.1.1. Informations à transmettre au promoteur

Chaque EvIG sera décrit sur le formulaire à cet effet (« Déclaration Initiale d'Evénement Indésirable Grave » ou « Déclaration de Suivi d'Evènement Indésirable Grave ») en essayant d'être le plus exhaustif possible. Les informations à transmettre sont les suivantes :

- identification du participant (numéro, code, date de naissance, date d'inclusion, sexe, poids, taille),
- gravité de l'EvI,
- date de début et de fin de l'EvI,
- description claire et détaillée de l'EvI (diagnostic, symptômes, intensité, chronologie, actions entreprises et résultats),
- évolution de l'EvI,

- maladies en cours ou antécédents pertinents du participant,
- traitements reçus par le participant,
- lien de causalité de l'EvI avec la séance de simulation, les éventuels traitements associés, la recherche ou d'autres critères.

L'investigateur doit également joindre au rapport d'EvIG, à chaque fois que possible :
- une copie du compte-rendu d'hospitalisation (si le participant a été hospitalisé),
- éventuellement, une copie du rapport d'autopsie,
- une copie de tous les résultats d'examens complémentaires réalisés, y compris les résultats négatifs pertinents en y joignant les valeurs normales du laboratoire,
- tout autre document qu'il juge utile et pertinent.

Ces documents seront anonymisés et porteront le n° d'identification du participant.

8.3.2.1.2. Modalités de notification au promoteur

Tout EvIG, quelle que soit sa relation de causalité avec la recherche (à l'exception de ceux qui sont recensés dans le protocole comme ne nécessitant pas une déclaration immédiate), doit être déclaré :
- par fax : 05 49 44 30 58.

On peut joindre le responsable de la Vigilance du CHU de Poitiers (Direction de la Recherche) le Dr. Sophie DURANTON par téléphone au 05.49.44.03.50 ou par fax au 05 49 44 30.58 ou par mail sophie.duranton@chu-poitiers.fr

8.3.2.1.3. Délai de notification au promoteur

Tout EvIG doit impérativement être déclaré dans les **24 heures** de sa survenue (ou dès que le médecin investigateur en a connaissance) au promoteur de l'étude.
La déclaration initiale peut être suivie de compléments d'informations pertinentes dans les **8 jours** en cas d'événement fatal ou menaçant le pronostic vital et dans les **15 jours** dans les autres cas.

8.3.2.1.4. Période de notification au promoteur

L'investigateur a la responsabilité de noter et de rapporter tous les EvIG survenant pendant l'ensemble de l'étude :
- à partir de la date de signature du consentement,
- pendant toute la durée de suivi du participant prévue par l'essai,
- et jusqu'à 4 semaines après la fin du suivi du participant.

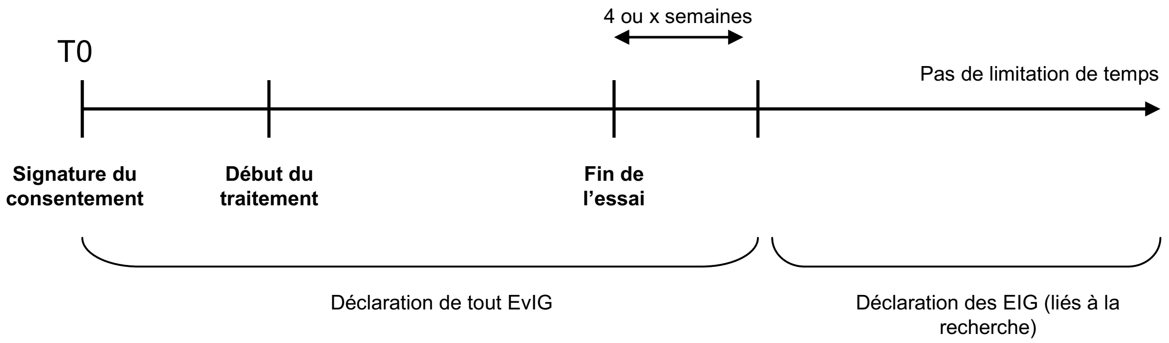

<u>Les évènements indésirables graves non soumis à déclaration immédiate</u> :
Certaines circonstances nécessitant une hospitalisation ne relèvent pas du critère de gravité « hospitalisation » et ne doivent pas être déclarées comme des EvIG :
- admission pour raison sociale ou administrative,
- hospitalisation pour traitement de routine ou surveillance de la pathologie étudiée non associé à une détérioration de l'état du participant,
- hospitalisation pour traitement médical ou chirurgical programmé avant le début de la recherche.

Notification des événements indésirables non graves
Tous les autres EvI seront rapportés sur le formulaire « événement indésirable » du cahier d'observation en précisant la date de survenue, la description, l'intensité, la durée, le mode de résolution, l'étiologie, l'imputabilité et les décisions prises.

8.3.3. Rôle du promoteur

8.3.3.1. Analyse des événements indésirables graves

Le promoteur doit évaluer :

- **la causalité des EvIG** (tous les évènements indésirables, pour lesquels l'investigateur ou le promoteur estime qu'une relation de causalité avec la stratégie peut être raisonnablement envisagée, sont considérés comme des suspicions d'effets indésirables. En cas d'évaluation différente du promoteur et de l'investigateur, les deux avis sont mentionnés sur la déclaration adressée à l'autorité compétente si cette déclaration est nécessaire),
- **et leur caractère attendu ou inattendu** en s'aidant du document de référence (brochure pour l'investigateur) en vigueur

8.3.3.2. Cotation de l'imputabilité

Conformément aux recommandations ICH sur la gestion des évènements indésirables dans les essais cliniques - ICH E2B(R3), version du 12 mai 2005 - une évaluation de l'imputabilité est réalisée pour tout EvIG déclaré. La méthode de cotation utilisée est la suivante :

- **Sans relation** : l'évènement apparaît dans un délai incompatible par rapport à la mise en place de la stratégie, et/ou il existe un nombre suffisant d'informations montrant que la réaction observée est sans rapport avec la stratégie, et/ou il existe une explication alternative vraisemblable.
- **Relation douteuse** : l'évènement a une chronologie (apparition, évolution) peu compatible avec la mise en place de la stratégie et est attribuable, selon toute vraisemblance, à d'autres facteurs que la stratégie, tels que l'état clinique du participant ou l'administration concomitante d'autres produits.
- **Relation possible** : l'évènement apparaît dans un délai compatible après la mise en place de la stratégie et bien que l'on ne puisse exclure la responsabilité de la stratégie, d'autres facteurs peuvent être mis en cause, tels que l'état clinique du participant ou l'administration concomitante d'autres produits. L'information sur l'évolution à l'arrêt de la stratégie peut être absente ou peu concluante.
- **Relation probable** : l'évènement apparaît dans un délai compatible après la mise en place de la stratégie. Il ne peut raisonnablement être dû à un autre facteur, tel que l'état clinique du participant ou l'administration concomitante d'autres produits. L'évolution à l'arrêt de la stratégie doit être cliniquement compatible. Une information sur la réintroduction de la stratégie n'est pas indispensable.
- **Relation hautement probable** : l'évènement apparaît dans un délai très suggestif après la mise en place de la stratégie. Il ne peut pas être expliqué par un autre facteur, tel que l'état clinique du participant ou l'administration concomitante d'autres produits. L'évolution à l'arrêt de la stratégie doit être cliniquement compatible. L'évènement est explicable sur un plan pharmacologique ou physiopathologique.

Les évènements indésirables ayant une relation douteuse, possible, probable ou hautement probable avec la stratégie sont considérés comme liés à celui-ci (ceux-ci). S'ils sont inattendus, ils sont qualifiés comme étant des EIGI et doivent faire l'objet d'une déclaration par le promoteur (cf. Paragraphe suivant).

8.3.3.3. Déclaration des effets indésirables graves inattendus

Le promoteur déclare tous les effets indésirables graves et inattendus (EIGI) à l'ANSM, au Comité de Protection des Personnes (CPP) et aux investigateurs. La déclaration réglementaire est faite dans un délai maximum de **7 jours** calendaires pour les effets indésirables graves inattendus. Des informations complémentaires pertinentes doivent être recherchées et transmises dans un nouveau délai de **7 jours**.

8.3.3.4. Transmission des rapports annuels de sécurité

A la date anniversaire du commencement effectif de la recherche, le promoteur rédige un rapport de sécurité comprenant :

- la liste des effets indésirables graves susceptibles d'être lié(s) à la stratégie de l'essai incluant les effets graves inattendus et attendus,
- une analyse concise et critique de la sécurité des participants se prêtant à la recherche.

Ce rapport peut être soumis à l'investigateur coordonnateur pour approbation. Il est envoyé à l'ANSM et au CPP dans les **60 jours** suivant la date anniversaire du commencement effectif de la recherche .

8.3.3.5. Déclaration des autres données de sécurité

Il s'agit de toute donnée de sécurité ou tout **fait nouveau** qui pourrait modifier significativement l'évaluation du rapport des bénéfices et des risques de la stratégie, ou de l'essai, ou qui pourrait conduire à envisager des modifications concernant la conduite de l'essai, comme par exemple :

a) toute augmentation cliniquement significative de la fréquence d'apparition d'un effet indésirable grave attendu ;
b) des suspicions d'EIGI survenus chez des participants ayant terminé l'essai et qui sont notifiés par l'investigateur au promoteur, ainsi que des rapports de suivi éventuels ;
c) tout fait nouveau concernant le déroulement de l'essai clinique ou le développement de la stratégie, lorsque ce fait nouveau est susceptible de porter atteinte à la sécurité des participants.
d) les recommandations du comité de surveillance indépendant, le cas échéant, si elles sont pertinentes pour la sécurité des personnes,
e) tout EIGI transmis au promoteur par un autre promoteur d'un essai clinique mené dans un pays tiers portant sur le même traitement non médicamenteux.

Le promoteur doit effectuer la déclaration à l'ANSM et au CPP dès que possible et au plus tard dans un délai de 7 jours calendairesà compter du moment où le promoteur en a eu connaissance. Des informations complémentaires pertinentes doivent être transmises, dans un nouveau délai de **7 jours.**

8.3.4. Exposition in utero

Il n'est pas attendu de grossesse au cours de l'étude (critères de non inclusion). Toute grossesse devra être déclarée immédiatement au promoteur.

L'investigateur informe le responsable de Pharmacovigilance des Essais Cliniques du promoteur et envoie par fax le formulaire d'EIG sur lequel doit être noté la date prévisible d'accouchement, les coordonnées de l'obstétricien et de la maternité prévue pour l'accouchement si la grossesse se poursuit.

L'investigateur doit suivre la patiente jusqu'au terme de la grossesse ou de son interruption et en notifier l'issue au promoteur.

Si l'issue de la grossesse entre dans le cadre de la définition des événements indésirables graves (avortement spontané avec hospitalisation, mort fœtale, anomalie congénitale, ...) l'investigateur doit suivre la procédure de déclaration des EIG.

8.3.5. Comité de Surveillance

Sans objet.

8.4. Modalités et durée du suivi des personnes suite à la survenue d'événements indésirables

Chaque événement indésirable sera suivi jusqu'à sa complète résolution (stabilisation à un niveau jugé acceptable par l'investigateur ou retour à l'état antérieur) même si le participant est sorti de la recherche.

9. STATISTIQUES

Logiciel utilisé : logiciel SAS version 9.0.

9.1. Description des méthodes statistiques prévues, y compris du calendrier des analyses intermédiaires prévues

9.1.1. Analyse descriptive

Les variables quantitatives (Scores utilisés, valeur du cortisol, de la fréquence cardiaque, de l'intervalle RR', ..) seront décrites par la moyenne, l'écart-type et/ou la médiane et l'intervalle

interquartile. Les variables qualitatives (âge, sexe, statut professionnel, années d'expérience professionnelle) seront résumées par l'effectif brut et le pourcentage correspondant pour chacune des modalités.

9.1.2. Evaluation de l'effet du stress sur la performance

La relation entre les différentes variables témoignant du niveau de stress (cortisol salivaire, paramètres du Holter, scores aux différentes échelles de stress) évaluées lors de la 1ère séance (stress basal) et lors des séances suivantes avec la performance (scores de performance globale, score VIO et échelles évaluant le travail en équipe) sera étudiée par le calcul d'un coefficient de corrélation de Pearson (ou d'un coefficient de corrélation de Spearman, si nécessaire). Ce calcul sera réalisé sur l'ensemble de la population de l'étude et dans chacun des groupes A et B. On recherchera une hétérogénéité liée au statut (médecins seniors, médecins juniors, infirmiers, ambulanciers).

9.1.3. Evaluation des variations des scores de performance

Les variations des scores de performance globale de l'équipe, des scores de performance de la pose d'une VIO et des échelles évaluant le travail en équipe seront évaluées au cours du temps. Dans chacun des groupes A (9 temps) et B (3 temps), la modification des scores entre les différentes séances de simulation sera testée à l'aide d'une ANOVA pour mesures répétées (ou d'un test non paramétrique de Friedman si nécessaire). Sur l'ensemble de la population d'étude des modèles de type modèles linéaires à effets mixtes pourront être envisagés de façon à prendre en compte dans la même analyse l'ensemble des données recueillies aux différentes séances, notamment le développement de stratégies de gestion du stress, en parallèle au stress et à la répétition des simulations.

9.1.4. Comparaison de la variation des scores de performance entre les groupes A et B = Evaluation de l'effet de la simulation répétée sur la performance

Pour étudier la variation de la performance (scores de performance globale, score VIO et scores aux échelles évaluant le travail en équipe) entre l'inclusion (T0) et la fin du programme de simulation, la variation relative des différents scores sera calculée (score final – score T0)/score T0. Ces variations relatives seront comparées entre les 2 groupes à l'aide d'un test de Student. On recherchera un effet statut du soignant à l'aide d'une ANOVA.

9.1.5. Comparaison du stress entre les groupes A et B = Evaluation de l'effet de la simulation répétée sur le stress

Les différents marqueurs du stress (biologique, électrophysiologique et psychologique) seront comparés entre les 2 groupes A et B à l'aide d'un test de Student ou d'un test ne paramétrique de Mann-Withney si nécessaire.

9.1.6. Reproductibilité inter-observateurs

Elle sera évaluée par le calcul du coefficient de corrélation intra-classe pour les différentes échelles d'évaluation utilisées.

9.2. Nombre prévu de patients à inclure dans cette étude et justification statistique

Le nombre de sujets nécessaires a été calculé pour répondre à l'objectif principal de l'étude : la mise en évidence d'une relation entre stress et performance.

On souhaite mettre en évidence une relation entre stress et performance telle que le coefficient de corrélation atteigne au moins la valeur de 0,50. Pour un risque de 1ère espèce de 5%, avec une puissance de l'ordre de 90% et en situation bilatérale le nombre de sujets à inclure est de 48 (Proc POWER, SAS). Nous inclurons 12 équipes de 4 personnes, chaque équipe comprenant les représentants de chaque catégorie (un médecin senior, un médecin junior (interne), un(e) infirmier(e) et un(e) ambulancier(e).

Pour répondre aux objectifs secondaires, les équipes seront réparties en 2 groupes randomisés A et B.

A noter que l'effectif de 12 personnes dans chacune des catégories professionnelles nous permettrait de mettre en évidence une différence de 2,1 points du score d'évaluation de la VIO sur 20 points (Oriot 2012), sachant que l'écart-type de ce score a été décrit à 1,02 dans une étude antérieure.

En revanche, nous ne disposons pas de renseignements suffisants dans la littérature pour évaluer le nombre de sujets nécessaires à la mise en évidence d'une différence sur la variabilité des différents scores. La puissance de notre étude pour les questions correspondant aux objectifs secondaires sera calculée *a posteriori* à partir des données réellement observées.

9.3. Degré de signification statistique prévu

Un $p<0.05$ sera considéré comme statistiquement significatif

9.4. Critères statistiques d'arrêt de la recherche

Aucun critère statistique d'arrêt de la recherche n'est défini car aucune analyse statistique intermédiaire n'est prévue.

9.5. Méthode de prise en compte des données manquantes, inutilisées ou non valides

Toutes les informations requises par le protocole doivent être consignées et une explication doit être apportée pour chaque donnée manquante.

9.6. Gestion des modifications apportées au plan d'analyse de la stratégie initiale

Les écarts au plan d'analyse statistique seront précisés et justifiés dans le rapport final d'analyse.

10. DROIT D'ACCES AUX DONNEES ET DOCUMENTS SOURCE

10.1. Accès aux données

Conformément aux BPC :

- le promoteur est chargé d'obtenir l'accord de l'ensemble des parties impliquées dans la recherche afin de garantir l'accès direct à tous les lieux de déroulement de la recherche, aux données source, aux documents source et aux rapports dans un but de contrôle de qualité et d'audit par le promoteur,
- les investigateurs mettront à disposition des personnes chargées du suivi, du contrôle de qualité ou de l'audit de la recherche biomédicale, les documents et données individuelles strictement nécessaires à ce contrôle, conformément aux dispositions législatives et réglementaires en vigueur (articles L.1121-3 et R.5121-13 du code de la santé publique).

10.2. Documents source

Les documents source étant définis comme tout document ou objet original permettant de prouver l'existence ou l'exactitude d'une donnée ou d'un fait enregistrés au cours de la recherche seront conservés pendant 15 ans par l'investigateur.

10.3. Confidentialité des données

Conformément aux dispositions concernant la confidentialité des données auxquelles ont accès les personnes chargées du contrôle de qualité d'une recherche biomédicale (article L.1121-3 du code de la santé publique), conformément aux dispositions relatives à la confidentialité des informations concernant notamment la nature des médicaments expérimentaux, les essais, les personnes qui s'y prêtent et les résultats obtenus (article R. 5121-13 du code de la santé publique), les personnes ayant un accès direct prendront toutes les précautions nécessaires en vue d'assurer la confidentialité des informations relatives aux médicaments expérimentaux, aux essais, aux personnes qui s'y prêtent et notamment en ce qui concerne leur identité ainsi qu'aux résultats obtenus.
Ces personnes, au même titre que les investigateurs eux-mêmes, sont soumises au secret professionnel (selon les conditions définies par les articles 226-13 et 226-14 du code pénal).
Pendant la recherche biomédicale ou à son issue, les données recueillies sur les personnes qui s'y prêtent et transmises au promoteur par les investigateurs (ou tous autres intervenants spécialisés) seront rendues anonymes.

Elles ne doivent en aucun cas faire apparaître en clair les noms des personnes concernées ni leur adresse.
Seules les initiales du nom et du prénom du participant seront enregistrées, accompagnées d'un numéro codé propre à l'étude indiquant l'ordre d'inclusion des sujets.
Le promoteur s'assurera que chaque personne qui se prête à la recherche a donné son accord par écrit pour l'accès aux données individuelles la concernant et strictement nécessaires au contrôle de qualité de la recherche

11. CONTROLE ET ASSURANCE DE LA QUALITE

Un Attaché de Recherche Clinique (ARC) mandaté par le promoteur s'assurera de la bonne réalisation de l'étude, du recueil et de la saisie des données, en accord avec les Procédures Opératoires Standards mises en application au sein du CHU de Poitiers et conformément aux Bonnes Pratiques Cliniques ainsi qu'aux dispositions législatives et réglementaires en vigueur.

L'investigateur et les membres de son équipe acceptent de se rendre disponibles lors des visites de Contrôle de Qualité effectuées à intervalles réguliers par l'Attaché de Recherche Clinique.
Lors de ces visites, les éléments suivant seront revus :

- consentement éclairé
- respect du protocole de l'étude et des procédures qui y sont définies
- qualité des données saisies : exactitude, données manquantes, cohérence des données avec les documents "source" (dossiers médicaux, carnets de rendez-vous, originaux des résultats de laboratoire, etc…)
- gestion des produits éventuels.

Les investigateurs s'engagent à accepter les audits d'assurance qualité effectués par le promoteur ainsi que les inspections effectuées par les Autorités Compétentes. Toutes les données, tous les documents et rapports peuvent faire l'objet d'audits et d'inspections réglementaires sans que puisse être opposé le secret médical.

12. CONSIDERATIONS ETHIQUES

12.1. Comité de Protection des Personnes

Le protocole, le formulaire d'information et l'attestation de consentement de l'étude seront soumis pour avis au Comité de Protection des Personnes Ouest III.
La notification de l'avis favorable du CPP sera transmise au promoteur de l'étude et à l'Autorité compétente.
Une demande d'autorisation sera adressée par le Promoteur à l'ANSM avant le début de l'étude.

12.2. Modifications substantielles

En cas de modification substantielle apportée au protocole par l'investigateur, elle sera approuvée par le promoteur. Ce dernier devra obtenir préalablement à sa mise en œuvre un avis favorable du CPP et une autorisation de l'ANSM dans le cadre de leurs compétences respectives.
Un nouveau consentement des personnes participant à la recherche sera recueilli si nécessaire.

12.3. Information du participant et formulaire de consentement éclairé écrit

Les participants seront informés de façon complète et loyale, en des termes compréhensibles, des objectifs et des contraintes de l'étude, des risques éventuels encourus, des mesures de surveillance et de sécurité nécessaires, de leurs droits de refuser de participer à l'étude ou de la possibilité de se rétracter à tout moment.
Toutes ces informations figurent sur un formulaire d'information et de consentement remis au participant.
Le consentement libre, éclairé et écrit du participant sera recueilli par l'investigateur, ou un médecin qui le représente avant l'inclusion définitive dans l'étude.

Le formulaire d'information et le consentement signé par les deux parties sera remise au participant, l'investigateur en conservera l'original.
Une copie sera placée en fin d'étude dans une enveloppe inviolable scellée regroupant l'ensemble des formulaires de consentement, celle-ci sera archivée par le promoteur.

12.4. Définition de la période d'exclusion

La période d'exclusion définie dans le cadre de cette étude est de 1 mois, période pendant laquelle le participant ne peut participer à un autre protocole de recherche clinique après la fin de l'étude.

12.5. Prise en charge relative à la recherche

Aucune

12.6. Indemnisation des sujets

Les participants ne seront pas rémunérés pour leur participation à la recherche.
L'apprentissage par simulation sur mannequin haute-fidélité en équipe étant lui-même facturé 300 € par personne et par jour. Les participants bénéficieront donc gracieusement de cet enseignement contre leur participation à la recherche.

12.7. Inscription au fichier national des personnes se prêtant à une recherche biomédicale

D'un point de vue réglementaire, les participants seront inscrits au fichier national des personnes se prêtant à une recherche biomédicale.

13. TRAITEMENT DES DONNEES ET CONSERVATION DES DOCUMENTS ET DONNEES

13.1. Cahier d'observation

Un CRF papier sera élaboré pour cette étude.
Toutes les informations requises par le protocole doivent être consignées sur les cahiers d'observation et une explication doit être apportée pour chaque donnée manquante. Les données devront être recueillies au fur et à mesure qu'elles sont obtenues, et transcrites dans ces cahiers de façon nette et lisible.
Les données erronées relevées sur les cahiers d'observation seront clairement barrées et les nouvelles données seront copiées, à côté de l'information barrée, accompagnées des initiales, de la date et éventuellement d'une justification par l'investigateur ou la personne autorisée qui aura fait la correction.

13.2. Saisie et traitement des données

La saisie des données sera réalisée sous Access par le technicien d'étude clinique avec l'aide des investigateurs.
L'analyse des données sera réalisée par le Dr Stéphanie RAGOT, statisticienne-méthodologiste.

13.3. CNIL

Cette étude entre dans le cadre de la « Méthodologie de Référence » (MR-001) en application des dispositions de l'article 54 alinéa 5 de la loi n°78-17 du 6 janvier 1978 modifiée relative à l'informatique, aux fichiers et aux libertés. Ce changement a été homologué par décision du 5 janvier 2006. Le CHU de Poitiers, promoteur de l'étude, a signé un engagement de conformité à cette « Méthodologie de Référence ».
Les déclarations devant être effectuées dans le cadre de la MR-001 seront traitées par le référent CIL (Comité Informatique et Liberté) du CHU de Poitiers.

13.4. Archivage

Les documents suivants seront archivés par le nom de l'étude dans les locaux du Laboratoire de Simulation de Poitiers jusqu'à la fin de la période d'utilité pratique.

Ces documents sont :
- Protocole et annexes, amendements éventuels,
- Formulaires d'information et consentements originaux signés
- Données individuelles (copies authentifiées de données brutes)
- Documents de suivi
- Analyses statistiques
- Rapport final de l'étude

A l'issue de la période d'utilité pratique, l'ensemble des documents à archiver, tels que définis dans la procédure de « classement et archivage des documents liés aux recherches biomédicales » du CHU de Poitiers sera transféré sur le site d'archivage (Service Central des Archives – CHU Poitiers) et sera placé sous la responsabilité du Promoteur pendant 15 ans après la fin de l'étude conformément aux pratiques institutionnelles.
Aucun déplacement ou destruction ne pourra être effectué sans l'accord du Promoteur. Au terme des 15 ans, le promoteur sera consulté pour destruction. Toutes les données, tous les documents et rapports pourront faire l'objet d'audit ou d'inspection.

14. ASSURANCE

Le Promoteur souscrit pour toute la durée de l'étude une assurance garantissant sa propre responsabilité civile ainsi que celle de tout intervenant impliqué dans la réalisation de l'étude. Il assurera également l'indemnisation intégrale des conséquences dommageables à la recherche pour la personne qui s'y prête et ses ayants droit, sauf preuve à sa charge que le dommage n'est pas imputable à sa faute ou à celle de tout intervenant, sans que puisse être opposé le fait d'un tiers ou le retrait volontaire de la personne qui avait initialement consenti à se prêter à la recherche.

15. FAISABILITE DE L'ETUDE

Cette recherche biomédicale a été considérée comme faisable à différents égards :
- La simulation est une méthode d'apprentissage en plein essor et peut intéresser de nombreux professionnels de santé de pouvoir en bénéficier gracieusement tout en participant à une recherche biomédicale.
- Le protocole de recherche fait d'une séance de simulation en équipe pluridisciplinaire suivi d'un débriefing orienté vers l'acquisition de savoir et de savoir-faire est attractif pour de nombreux professionnels qui n'ont jamais eu cette possibilité d'apprentissage.
- Les locaux du Laboratoire de Simulation – SiMI offre un environnement très accueillant, à la fois pour le temps de la séance de simulation, que pour le débriefing que la phase de « wash-out » des 3 heures qui suivent, dans une ambiance conviviale.
- Enfin, nous avons obtenu un vif intérêt pour cette étude par le personnel du CHU de Poitiers, ce qui rendra le recrutement sur la base du volontariat très facile.

16. REGLES RELATIVES A LA PUBLICATION

Les communications, rapports scientifiques et les publications correspondant à cette étude seront réalisés sous la responsabilité de l'investigateur principal coordonnateur de l'étude et du responsable scientifique. Les coauteurs du rapport et des publications seront les cliniciens impliqués, au prorata de leur contribution à l'étude, ainsi que les biostatisticiens-méthodologistes et les chercheurs associés.
Les règles de publications suivront les recommandations internationales (N Engl J Med, 1997; 336: 309-315).

17. PROPRIETE – EXPLOITATIONS DES RESULTATS ET VALORISATION

Le Promoteur est seul propriétaire des résultats de la recherche. Il est seul habilité à déposer en priorité toute demande de brevet et extensions y compris, tant en France qu'à l'étranger, en son nom propre, couvrant les résultats de la recherche.
Le Promoteur communiquera, à la demande, au centre associé, les résultats de la Recherche dans un rapport final, au plus tard un (1) an suivant la fin de la Recherche

18. LISTE DES ANNEXES

Annexe 1 : Notice d'information du patient
Annexe 2 : Formulaire de consentement éclairé
Annexe 3 : Déclaration d'Helsinki
Annexe 4 : Formulaire de déclaration d'un événement indésirable grave
Annexe 5 : Echelle d'évaluation de la performance globale d'une équipe
Annexe 6 : Echelle d'évaluation de la pose d'une voie intra-osseuse IOPAS
Annexe 7 : Echelle d'évaluation du leadership BAT
Annexe 8 : Echelle clinique du travail en équipe CTS
Annexe 9 : Echelle de mesure de l'anxiété (fond anxieux et accès anxieux) STAI
Annexe 10 : Questionnaire de stress post-événementiel avec l'échelle IES-R
Annexe 11 : Questionnaire de stress post-événementiel avec l'échelle PCLS
Annexe 12 : Stratégies de gestion du stress (Overview of surgical coping strategies)

Annexe 1 : Notice d'information du participant à la recherche

SIM-STRESS :
EFFETS DU STRESS ET DE LA SIMULATION SUR LA PERFORMANCE D'UNE EQUIPE LORS DE LA PRISE EN CHARGE D'UN NOURRISSON EN ETAT DE CHOC

Madame, Mademoiselle, Monsieur

Le **Docteur Aïham GHAZALI** vous propose de participer à un travail de recherche biomédicale, intitulé : « ***Effets du stress et de la simulation sur la performance d'une équipe lors de la prise en charge d'un nourrisson en état de choc*** », dont le Centre Hospitalier Universitaire de Poitiers (2 rue de la Milétrie, 86021 POITIERS-05.49.44.46.65) est le promoteur.

Le protocole enregistré sous le numéro ID-RCB : 2013-A00648-37 ainsi que la lettre d'information et le consentement éclairé de cette étude, dont le promoteur est le CHU de Poitiers, ont obtenu l'avis favorable du Comité de Protection des Personnes Ouest III (CPP Ouest III) le XX/XX/201X, ainsi que l'autorisation à l'Agence Nationale de Sécurité du Médicament et des produits de santé (ANSM), le XX/XX/201X.

Délai de réflexion

Le document d'information qui vous est remis, va vous permettre de décider si vous souhaitez ou non, prendre part à cette étude. Nous vous remercions de prendre le temps de lire attentivement ce qui suit. N'hésitez pas à demander aux membres de l'équipe de recherche du Laboratoire de Simulation toutes les explications qui vous paraîtront nécessaires.
Votre participation est entièrement volontaire. Si vous ne désirez pas prendre part à cette étude, vos relations professionnelles avec les personnes encadrant la recherche ne seront en aucun cas modifiées.

Justification de l'étude

Il est connu que le stress peut altérer la performance, mais il n'y aucune données dans la littérature sur le bénéfice de la répétition de séances de simulation sur l'importance du stress et sa relation avec la performance obtenue.

Objectif de l'étude

Il s'agit d'une recherche translationnelle – entièrement en simulation – visant à évaluer **l'influence du stress sur la performance d'une équipe**, composée d'un médecin senior, d'un(e) interne, d'un(e) infirmier(ère) et d'un(e) ambulancier(e).
Par ailleurs, le 2e objectif de ce travail est de répéter les séances de simulation et de voir l'influence de la répétition sur la performance de l'équipe.

Déroulement de l'étude

Si vous acceptez de participer à cette recherche, vous serez sollicité pour **participer à des séances de simulation en équipe**.
Au maximum, une fois toutes les 6 semaines (9 séances de simulation en 12 mois) et au minimum, une fois tous les 6 mois (3 séances de simulation en 12 mois). Chaque séance dure 1 heure au maximum, incluant le débriefing.

Les éléments qui nous permettront de mesurer la performance sont des échelles validées sur certains gestes d'urgence et le travail en équipe. De même, toutes les séances de simulation feront l'objet d'un **enregistrement vidéo**. L'utilisation ultérieure de ces images vidéo ne se fera que dans le cadre de la recherche.

Les éléments qui permettront de **mesurer le stress** de façon fiable sont le Holter, le cortisol salivaire et trois questionnaires. Il n'y aura aucun prélèvement sanguin.
Le **Holter** vous sera posé la veille au soir (vers 18h) du jour de la simulation et gardé 3 heures après (total de 24 heures). Une mesure ponctuelle de la pression artérielle et de la fréquence cardiaque (pour évaluer le système nerveux autonome en condition de stress) sera effectué quatre fois : une la veille de la séance de simulation (vers 18h), le jour de la séance de simulation, juste avant et après la séance (avant le débriefing) et après le débriefing.

Le **cortisol salivaire** sera analysé (1 ml de salive) par quatre prélèvements salivaires, un la veille de la séance de simulation (vers 18h), le jour de la séance de simulation, juste avant et après la séance (avant le débriefing) et après le débriefing.
Les **questionnaires de stress** devront être remplis la veille, avant et après la séance de simulation par le remplissage d'un questionnaire sur votre état d'anxiété (la veille, avant et après la séance de simulation, après le débriefing). Deux autres questionnaires, seront remplis par courriel à 7 jours et à 1 mois après la séance de simulation afin d'évaluer votre stress post-événementiel.

Cette étude demande une participation de votre part d'environ 60 minutes par séance, 3 fois ou 9 fois pendant un an, comprenant à chaque fois une séance de simulation (au maximum 30 mn) évaluée par deux superviseurs indépendants, au cours de laquelle vous jouerez un rôle équivalent à votre statut professionnel, de la façon la plus naturelle possible.

Suite à la simulation, vous resterez au repos dans une salle dédiée à cet effet au sein du Laboratoire de Simulation, jusqu'à 17h, correspondant à l'heure de fin des explorations.

Bénéfices attendus

Le **bénéfice** que vous pouvez en tirer est de recevoir gracieusement un enseignement pratique innovant et évalué, sur les difficultés médicales et relationnelles rencontrées lors de prises en charge pédiatriques complexes.

Risques potentiels

Les **risques** liés à la participation sont l'inconfort causé par le port du Holter pendant 24 heures et la réalisation de trois prélèvements salivaires.

Participation volontaire

Votre participation à la recherche est tout à fait volontaire et n'entraînera aucun coût. Vous êtes libre d'accepter ou de refuser de participer. Vous êtes libre de changer d'avis à tout moment et de retirer votre consentement sans avoir à vous justifier et votre décision ne portera aucun préjudice à vos relations professionnelles avec les personnes encadrant la recherche.
Vos données relatives à la recherche ne seront analysées que si vous nous donnez votre accord.
Si des informations nouvelles apparaissaient en cours d'étude, pouvant remettre en cause votre accord de participation, vous en seriez immédiatement informés.
Si cette étude devait être arrêtée ou si votre participation devait être interrompue, vos relations professionnelles avec les personnes encadrant la recherche ne seraient en aucun cas modifiées. Vous êtes libre d'interrompre votre participation à tout moment.

Confidentialité des données

Dans le cadre de la recherche biomédicale à laquelle le CHU de Poitiers vous propose de participer, un traitement de vos données personnelles va être mis en œuvre pour permettre d'analyser les résultats de la recherche au regard de l'objectif de cette dernière qui vous a été présentée.
A cette fin, des données physiologiques vous concernant et les données relatives à vos habitudes de vie, seront transmises au Promoteur de la Recherche. Ces données seront identifiées par vos initiales, et un numéro de code. Ces données pourront également, dans des conditions assurant leur confidentialité, être transmises aux autorités de santé françaises et étrangères.
Conformément aux dispositions de loi relative à l'informatique aux fichiers et aux libertés, vous disposez d'un droit d'accès et de rectification. Vous disposez également d'un droit d'opposition à la transmission des données couvertes par le secret professionnel susceptibles d'être utilisées dans le cadre de cette recherche et d'être traitées.
Vous pouvez également avoir accès aux résultats globaux de l'étude vous concernant sur demande auprès du **Docteur Aïham GHAZALI** (Centre Hospitalier Universitaire de Poitiers - Service des Urgences-SAMU 86, 2 rue de la Milétrie, 86021 POITIERS Cedex - 05.49.44.37.08) mais également accéder directement ou par l'intermédiaire d'un médecin de votre choix à l'ensemble de vos données médicales en application des dispositions de l'article L1111-7 du Code de la Santé Publique.

Considérations éthiques

L'étude sera conduite en conformité avec :

- la déclaration d'Helsinki actualisée (en date d'octobre 2008),
- les Bonnes Pratiques Cliniques édictées par la Communauté européenne

par ICH (Conférence Internationale sur l'Harmonisation est un consensus sur la définition d'une approche harmonisée en matière de bonnes pratiques cliniques),

la loi de Santé Publique n°2004-806 du 9 août 2004 (remplaçant la loi 88-1138 du 20 décembre 1988 modifiée dite « loi Huriet-Sérusclat, relative à la protection des personnes se prêtant à des recherches biomédicales (dites loi Huriet)
et la loi « informatique et liberté ».

Prise en charge

Conformément à la législation en vigueur, le Centre Hospitalier Universitaire de Poitiers a souscrit une assurance auprès de la compagnie SHAM (n°131467), couvrant tous les risques éventuels liés à cette recherche. Cependant, seules les personnes bénéficiant d'un régime de Sécurité Sociale ou d'un régime assimilé seront autorisées à participer à cette étude.

Vous pourrez à tout moment interroger le **Professeur Denis ORIOT**, tel : 05-49-45-43-51, mail : simi.recherche@univ-poitiers.fr pour obtenir des informations complémentaires sur la recherche.

Après avoir lu ce document et obtenu toutes les réponses à vos questions, si vous acceptez de participer à la recherche nous vous demandons de bien vouloir confirmer votre décision en signant le formulaire de consentement éclairé ci-joint. Vous conserverez un exemplaire original de tout le document.

<u>Annexe 2</u> : **Formulaire de consentement du participant à la recherche**

SIM-STRESS :
EFFETS DU STRESS ET DE LA SIMULATION SUR LA PERFORMANCE D'UNE EQUIPE LORS DE LA PRISE EN CHARGE D'UN NOURRISSON EN ETAT DE CHOC

Je soussigné(e), Mme, Melle, Mr (Nom, Prénom) : ....
Né(e) le ....

certifie avoir reçu la lettre d'information concernant l'étude « ***Effets du stress et de la simulation sur la performance d'une équipe lors de la prise en charge d'un nourrisson en état de choc*** », dont le CHU de Poitiers (2 rue de la Milétrie, 86021 POITIERS-05.49.44.46.65) se porte promoteur et dont l'investigateur coordonnateur est le **Docteur Aïham GHAZALI** (Centre Hospitalier Universitaire de Poitiers - Service des Urgences-SAMU 86, 2 rue de la Milétrie, 86021 POITIERS Cedex, **05.49.44.37.08)**

Je déclare avoir disposé d'un temps de réflexion suffisant entre l'information et le consentement.
J'ai eu le temps de la lire et de poser toutes les questions que je souhaitais, et avoir été informé(e) par le Docteur ……………………………………………, des objectifs, des bénéfices, des risques potentiels et des contraintes de cette étude.
J'accepte librement et volontairement de participer à la recherche décrite ci-dessus et je suis parfaitement conscient(e) que je peux retirer à tout moment mon consentement de participation à cette recherche sans avoir à me justifier, ni à engager aucunement ma responsabilité. Ceci n'entraînera en aucune manière des conséquences sur la qualité des relations professionnelles avec les personnes encadrant la recherche.
Par ailleurs, je dois aussi être affilié(e) à, ou bénéficier d'un régime de Sécurité Sociale. Je confirme que c'est bien le cas.
Mon consentement ne décharge en rien le promoteur et l'investigateur de leurs responsabilités morales et légales et je conserve tous mes droits garantis par la loi.
J'ai pris connaissance que le protocole n°ID-RCB :2013-A00648-37 ainsi que la lettre d'information et le consentement éclairé de cette étude, dont le promoteur est le CHU de Poitiers, ont obtenu l'avis favorable du Comité de Protection des Personnes Ouest III (CPP Ouest III) le XX/XX/201X, ainsi que l'autorisation à l'Agence Nationale de Sécurité du Médicament et des produits de santé (ANSM), le XX/XX/201X.
Le promoteur de l'étude a souscrit une assurance de responsabilité civile en cas de préjudice auprès de la société SHAM (numéro de contrat : 131467) conformément à la loi.
Je déclare en outre avoir été informé et avoir accepté, que les données enregistrées à l'occasion de cette recherche puissent faire l'objet d'un traitement informatisé par le promoteur ou pour son compte. Elles ne pourront être consultées que par l'investigateur et ses collaborateurs, par des personnes mandatées par le promoteur et astreintes au secret professionnel et par des personnes mandatées par les autorités sanitaires et judiciaires.
J'ai bien noté que le droit d'accès et de rectification, prévu par la Loi « Informatique et Libertés » (Article 40) s'exerce à tout moment auprès du Docteur Aïham GHAZALI et que je dispose d'un droit de rectification de toutes ces informations par son intermédiaire en cas de données incomplètes, inexactes ou équivoques.
Selon la législation cette étude a fait l'objet d'une déclaration à la CNIL (Commission Nationale Informatique et Libertés).
Ce consentement est fait en trois exemplaires, un m'est remis, un autre reste en possession de l'investigateur et le dernier est remis au promoteur sous enveloppe scellée**.**
J'accepte librement et volontairement de participer à cette recherche dans les conditions décrites ci-dessus. J'accepte la prise de photographies et de vidéos dans l'unique but de la recherche.

Date : Signature du participant Signature de l'investigateur

Annexe 3 : Déclaration d'Helsinki

ASSOCIATION MEDICALE MONDIALE

DECLARATION D'HELSINKI

Principes éthiques applicables à la recherche médicale impliquant des êtres humains

Adoptée par la 18e Assemblée générale de l'AMM, Helsinki, Finlande, Juin 1964 et amendée par les

- 29e Assemblée générale de l'AMM, Tokyo, Octobre 1975
- 35e Assemblée générale de l'AMM, Venise, Octobre 1983
- 41e Assemblée générale de l'AMM, Hong Kong, Septembre 1989
- 48e Assemblée générale de l'AMM, Somerset West (Afrique du Sud), Octobre 1996
- 52e Assemblée générale de l'AMM, Edimbourg, Ecosse, Octobre 2000
- 53e Assemblée générale de l'AMM, Washington, Etats Unis, 2002 (ajout d'une note de clarification pour le paragraphe 29)
- 55e Assemblée générale de l'AMM, Tokyo, Japon 2004 (ajout d'une note de clarification concernant le paragraphe 30)
- 59e Assemblée générale de l'AMM, Séoul, Corée, Octobre 2008

A. INTRODUCTION

1. L'Association Médicale Mondiale (AMM) a élaboré la Déclaration d'Helsinki comme un énoncé de principes éthiques applicables à la recherche médicale impliquant des êtres humains, y compris la recherche sur du matériel biologique humain et sur des données identifiables.
La Déclaration est conçue comme un tout indissociable. Aucun paragraphe ne peut être appliqué sans tenir compte de tous les autres paragraphes pertinents.

2. Cette Déclaration s'adresse principalement aux médecins. L'AMM invite cependant les autres participants à la recherche médicale impliquant des êtres humains à adopter ces principes.

3. Le devoir du médecin est de promouvoir et de sauvegarder la santé des patients, y compris celles des personnes impliquées dans la recherche médicale. Le médecin consacre son savoir et sa conscience à l'accomplissement de ce devoir.

4. La Déclaration de Genève de l'AMM engage les médecins en ces termes: «La santé de mon patient prévaudra sur toutes les autres considérations » et le Code International d'Ethique Médicale déclare qu'un «médecin doit agir dans le meilleur intérêt du patient lorsqu'il le soigne».

5. Le progrès médical est basé sur la recherche qui, en définitive, doit comprendre des études impliquant des êtres humains. Des possibilités appropriées de participer à la recherche médicale devraient être offertes aux populations qui y sont sous représentées.

6. Dans la recherche médicale impliquant des êtres humains, le bien-être de chaque personne impliquée dans la recherche doit prévaloir sur tous les autres intérêts.

7. L'objectif premier de la recherche médicale impliquant des êtres humains est de comprendre les causes, le développement et les effets des maladies et d'améliorer les

interventions préventives, diagnostiques et thérapeutiques (méthodes, procédures et traitements). Même les meilleures interventions courantes doivent être évaluées en permanence par des recherches portant sur leur sécurité, leur efficacité, leur pertinence, leur accessibilité et leur qualité.

8. Dans la pratique médicale et la recherche médicale, la plupart des interventions comprennent des risques et des inconvénients.

9. La recherche médicale est soumise à des normes éthiques qui promeuvent le respect de tous les êtres humains et qui protègent leur santé et leurs droits. Certaines populations faisant l'objet de recherches sont particulièrement vulnérables et ont besoin d'une protection spéciale. Celles-ci incluent les personnes qui, d'elles-mêmes, ne sont pas en mesure de donner ou de refuser leur consentement et celles qui peuvent être vulnérables à la coercition ou à des influences indues.

10. Dans la recherche médicale impliquant des êtres humains, les médecins devraient tenir compte des normes et standards éthiques, légaux et réglementaires applicables dans leur propre pays ainsi que des normes et standards internationaux. Les protections garanties par la présente Déclaration aux personnes impliquées dans la recherche ne peuvent être restreintes ou exclues par aucune disposition éthique, légale ou réglementaire, nationale ou internationale.

B. PRINCIPES APPLICABLES A TOUS LES TYPES DE RECHERCHE MEDICALE

11. Il est du devoir des médecins participant à la recherche médicale de protéger la vie, la santé, la dignité, l'intégrité, le droit à l'auto-détermination, la vie privée et la confidentialité des informations des personnes impliquées dans la recherche.

12. La recherche médicale impliquant des êtres humains doit se conformer aux principes scientifiques généralement acceptés, se baser sur une connaissance approfondie de la littérature scientifique, sur d'autres sources pertinentes d'informations et sur des expériences appropriées en laboratoire et, le cas échéant, sur les animaux. Le bien être des animaux utilisés dans la recherche doit être respecté.

13. Une prudence particulière s'impose dans la conduite de recherches susceptibles de nuire à l'environnement.

14. La conception et la conduite de toutes les études impliquant des êtres humains doivent être clairement décrites dans un protocole de recherche. Ce protocole devrait contenir une déclaration sur les enjeux éthiques en question et indiquer comment les principes de la présente Déclaration ont été pris en considération. Le protocole devrait inclure des informations concernant le financement, les promoteurs, les affiliations institutionnelles, d'autres conflits d'intérêts potentiels, les incitations pour les personnes impliquées dans la recherche et les mesures prévues pour soigner et/ou dédommager celles ayant subis un préjudice en raison de leur participation à l'étude. Le protocole devrait mentionner les dispositions prévues après l'étude afin d'offrir aux personnes impliquées un accès aux interventions identifiées comme bénéfiques dans le cadre de l'étude ou à d'autres soins ou bénéfices appropriés.

15. Le protocole de recherche doit être soumis à un comité d'éthique de la recherche pour évaluation, commentaires, conseils et approbation avant que l'étude ne commence. Ce comité doit être indépendant du chercheur, du promoteur et de toute autre influence indue. Il doit prendre en considération les lois et réglementations du ou des pays où se déroule la recherche, ainsi que les normes et standards internationaux, mais ceux-ci ne doivent pas permettre de restreindre ou exclure l'une des protections garanties par la présente Déclaration aux personnes impliquées dans la recherche. Le comité doit avoir un droit de suivi sur les études en cours. Le chercheur doit fournir au comité des informations sur le suivi, notamment concernant tout évènement indésirable grave. Aucune modification ne peut être apportée au protocole sans évaluation et approbation par le comité.

16. La recherche médicale impliquant des êtres humains doit être conduite uniquement par des personnes scientifiquement qualifiées et expérimentées. La recherche impliquant des patients ou des volontaires en bonne santé nécessite la supervision d'un médecin ou d'un autre professionnel de santé qualifié et compétent. La responsabilité de protéger les personnes impliquées dans la recherche doit toujours incomber à un médecin ou à un autre professionnel de santé et jamais aux personnes impliquées dans la recherche même si celles-ci ont donné leur consentement.

17. La recherche médicale impliquant une population ou une communauté défavorisée ou vulnérable se justifie uniquement si la recherche répond aux besoins et priorités sanitaires de cette population ou communauté et si, selon toute vraisemblance, les résultats de la recherche seront bénéfiques à cette population ou communauté.

18. Toute recherche médicale impliquant des êtres humains doit préalablement faire l'objet d'une évaluation soigneuse des risques et des inconvénients prévisibles pour les personnes et les communautés impliquées dans la recherche, par rapport aux bénéfices

prévisibles pour elles et les autres personnes ou communautés affectées par la pathologie étudiée.

19. Tout essai clinique doit être enregistré dans une banque de données accessible au public avant que ne soit recruté la première personne impliquée dans la recherche.

20. Les médecins ne sont pas autorisés à participer à une recherche impliquant des êtres humains sans avoir la certitude que les risques inhérents ont été correctement évalués et pourront être gérés de manière satisfaisante. Les médecins doivent cesser immédiatement une étude dès que les risques s'avèrent dépasser les bénéfices potentiels ou dès l'instant où des résultats positifs et bénéfiques ont été démontrés.

21. Une recherche médicale impliquant des êtres humains ne peut être conduite que si l'importance de l'objectif dépasse les risques et inconvénients inhérents pour les personnes impliquées dans la recherche.

22. La participation de personnes capables à une recherche médicale doit être un acte volontaire. Bien qu'il puisse être opportun de consulter les membres de la famille ou les responsables de la communauté, aucune personne capable ne peut être impliquée dans une étude sans qu'elle ait donné librement son consentement.

23. Toutes les précautions doivent être prises pour protéger la vie privée et la confidentialité des informations personnelles concernant les personnes impliquées dans la recherche, et pour minimiser l'impact de l'étude sur leur intégrité physique, mentale et sociale.

24. Dans la recherche médicale impliquant des personnes capables, toute personne pouvant potentiellement être impliquée dans la recherche doit être correctement informé des objectifs, des méthodes, des sources de financement, de tout éventuel conflit d'intérêts, des affiliations institutionnelles du chercheur, des bénéfices escomptés et des risques potentiels de l'étude, des désagréments qu'elle peut engendrer et de tout autre aspect pertinent de l'étude. La personne pouvant potentiellement être impliquée dans la recherche doit être informé de son droit de refuser de participer à l'étude ou de s'en retirer à tout moment sans mesure de rétorsion. Une attention particulière devrait être accordée aux besoins d'informations spécifiques de chaque personne pouvant potentiellement être impliquée dans la recherche ainsi qu'aux méthodes adoptées pour fournir les informations. Lorsque le médecin ou une autre personne qualifiée en la matière a la certitude que la personne concernée a compris les informations, il doit alors solliciter son consentement libre et éclairé, de préférence par écrit. Si le consentement ne peut pas être donné par écrit, le consentement non écrit doit être formellement documenté en présence d'un témoin.

25. Pour la recherche médicale utilisant des tissus ou des données d'origine humaine, les médecins doivent normalement solliciter le consentement pour le prélèvement, l'analyse, le stockage et/ou la réutilisation. Il peut se présenter des situations où il est impraticable, voire impossible d'obtenir le consentement ou que cela mettrait en péril la validité de la recherche. Dans de telles situations, la recherche peut être entreprise uniquement après évaluation et approbation d'un comité d'éthique de la recherche.

26. Lorsqu'il sollicite le consentement éclairé d'une personne pour sa participation à une recherche, le médecin devrait être particulièrement attentif lorsque cette dernière est dans une relation de dépendance avec lui ou pourrait donner son consentement sous la contrainte. Dans ce cas, le consentement éclairé devrait être sollicité par une personne qualifiée en la matière et complètement indépendante de cette relation.

27. Lorsque la recherche implique des personnes incapables, le médecin doit solliciter le consentement éclairé de leur représentant légal. Les personnes incapables ne doivent pas être inclues dans une étude qui n'a aucune chance de leur être bénéfique sauf si cette étude vise à améliorer la santé de la population qu'elles représentent, qu'elle ne peut pas être réalisée avec des personnes capables et qu'elle ne comporte que des risques et des inconvénients minimes.

28. Lorsqu'une personne considérée comme incapable est en mesure de donner son assentiment concernant sa participation à la recherche, le médecin doit solliciter cet assentiment en complément du consentement de son représentant légal. Le refus de la personne pouvant potentiellement être impliquée dans la recherche devrait être respecté.

29. La recherche impliquant des personnes physiquement ou mentalement incapables de donner leur consentement, par exemple des patients inconscients, peut être menée uniquement si l'état physique ou mental empêchant de donner un consentement éclairé est une caractéristique nécessaire de la population sur laquelle porte cette recherche. Dans de telles circonstances, le médecin devrait solliciter le consentement éclairé du représentant légal. En l'absence d'un représentant légal et si la recherche ne peut pas être retardée, l'étude peut être lancée sans le consentement éclairé. Dans ce cas, le protocole de recherche doit mentionner les raisons spécifiques d'impliquer des personnes dont l'état les rend incapables de donner leur consentement éclairé et l'étude doit être approuvée par un comité d'éthique de la recherche. Le consentement pour maintenir la personne concernée dans la recherche devrait, dès que possible, être obtenu de la personne elle-même ou de son représentant légal.

30. Les auteurs, rédacteurs et éditeurs ont tous des obligations éthiques concernant la publication des résultats de recherche. Les auteurs ont le devoir de mettre à la disposition du public les résultats de leurs recherches sur les êtres humains. Ils ont la responsabilité de fournir des rapports complets et précis. Ils devraient se conformer aux directives acceptées en matière d'éthique pour la rédaction de rapports. Les résultats aussi bien négatifs et non concluants que positifs devraient être publiés ou rendus publics par un autre moyen. La publication devrait mentionner les sources de financement, les affiliations institutionnelles et les conflits d'intérêts. Les rapports de recherche non-conformes aux principes de la présente Déclaration ne devraient pas être acceptés pour publication.

C. PRINCIPES ADDITIONNELS POUR LA RECHERCHE MEDICALE ASSOCIEE A DES SOINS MEDICAUX

31. Le médecin peut associer la recherche médicale à des soins médicaux uniquement dans la mesure où la recherche se justifie par sa valeur potentielle en matière de prévention, de diagnostic ou de traitement et si le médecin a de bonnes raisons de penser que la participation à l'étude ne portera pas atteinte à la santé des patients concernés.

32. Les bénéfices, les risques, les inconvénients, ainsi que l'efficacité d'une nouvelle intervention doivent être testés et comparés à ceux de la meilleure intervention courante avérée, sauf dans les circonstances suivantes :
- L'utilisation de placebo, ou le fait de ne pas administrer de traitement, est acceptable lorsqu'il n'existe pas d'intervention courante avérée; ou
- l'utilisation d'un placebo afin de déterminer l'efficacité ou la sécurité d'une intervention est nécessaire pour des raisons de méthodologie incontournables et scientifiquement fondées, et les patients recevant le placebo ou aucun traitement ne courent aucun risque de préjudices graves ou irréversibles. Le plus grand soin doit être apporté afin d'éviter tout abus de cette option.

33. A la fin de l'étude, les patients impliqués ont le droit d'être informés des conclusions de l'étude et de profiter de tout bénéfice en résultant, par exemple, d'un accès aux interventions identifiées comme bénéfiques dans le cadre de l'étude ou à d'autres soins ou bénéfices appropriés.

34. Le médecin doit fournir des informations complètes au patient sur la nature des soins liés à la recherche. Le refus d'un patient de participer à une étude ou sa décision de s'en retirer ne doit jamais interférer avec la relation patient-médecin.

35. Dans le cadre du traitement d'un patient, faute d'interventions avérées ou faute d'efficacité de ces interventions, le médecin, après avoir sollicité les conseils d'experts et avec le consentement éclairé du patient ou de son représentant légal, peut recourir à une intervention non avérée si, selon son appréciation professionnelle, elle offre une

chance de sauver la vie, rétablir la santé ou alléger les souffrances du patient. Dans toute la mesure du possible, cette intervention devrait faire l’objet d’une recherche pour en évaluer la sécurité et l’efficacité. Dans tous les cas, les nouvelles informations devraient être enregistrées et, le cas échéant, rendues publiques.

Annexe 4 : Formulaire de déclaration d'un événement indésirable grave

	DECLARATION D'EVENEMENT INDESIRABLE GRAVE (EvIG)	Etude Sim-Stress

EvIG n° |__| : ☐ Déclaration initiale ☐ Déclaration de suivi n°.......

1- PATIENT

Numéro : |__|__|-|__|__|__|__| Initiales : |__|-|__|

Sexe : M ☐ F ☐ Poids (kg) : |__|__|__| Taille (cm) : |__|__|__|

Date de naissance : |__|__| |__|__| |__|__|__|__|

Date d'inclusion : |__|__| |__|__| |__|__|

Antécédents / facteurs de risque en rapport avec l'évènement *(préciser les dates de début et de fin)* :

....

....

....

2- EVENEMENT INDESIRABLE GRAVE

Critère de gravité de l'évènement

☐ Décès
☐ Mise en jeu du pronostic vital
☐ Hospitalisation ou prolongation d'hospitalisation*
  * Date de début : |__|__| |__|__| |__|__|
  Date de fin : |__|__| |__|__| |__|__| ou ☐ en cours
☐ Incapacité ou invalidité importante ou durable
☐ Malformation / Anomalie congénitale
☐ Evènement potentiellement grave / Situation médicalement significative

Description de l'évènement (⇒ Joindre les comptes-rendus et les résultats d'examens complémentaires par fax)

....

....

....

....

....

....

....

....

....

....

....

....

....

→ **Diagnostic retenu** : ....

....

Date de début de l'évènement (1ères manifestations) : |__|__| |__|__| |__|__|

Date d'apparition du critère de gravité : |__|__| |__|__| |__|__|

Mesures prises – Traitements correcteurs :

....

....

....

Evolution de l'évènement :

☐ Résolu sans séquelles
☐ Résolu avec séquelles : ....
} Date de résolution : |__|__| |__|__| |__|__|

☐ En cours de résolution (amélioration)*
☐ Non résolu (état inchangé ou aggravé) *
} *Une déclaration de suivi devra être complétée

☐ Evolution inconnue*
☐ Décès

Patient décédé : Date : |__|__| |__|__| |__|__| Cause : .... En lien avec l'EvIG ? ☐ oui ☐ non

3- PROCEDURE(S) EXPERIMENTALE(S)

Description	Date et heure de début	Date et heure de fin	Arrêt prématuré de la procédure ?	Causalité de l'EvIG<br>0 : non lié à la procédure<br>1 : lié à la procédure<br>2 : ne sait pas	Commentaires

4- TRAITEMENTS CONCOMITANTS

- Noter tous les médicaments pris par le patient avant la survenue de l'événement.
- Indiquer, si applicable, les modifications de traitement suite à l'évènement.
- Donner votre avis sur le lien de causalité entre l'évènement et chaque médicament.

Médicaments et voies d'administration	Indication	Posologie	Date de début	Action prise<br>0 : poursuite<br>1 : arrêt<br>2: ↘ posologie<br>3: ↗ posologie<br>4 : ne sait pas	Date de l'action	Après l'action, l'EvIG :<br>0 : a disparu<br>1 : s'est amélioré<br>2 : est inchangé/aggravé<br>3 : inconnu	Causalité de l'EvIG<br>0 : non lié au médicament<br>1 : lié au médicament<br>2 : ne sait pas

L'évènement est-il réapparu en cas de réintroduction d'un médicament arrêté ? ☐ oui ☐ non ☐ non applicable
Si oui, de quel(s) médicament(s) s'agit-il ? : ....

Autres traitements non médicamenteux	Indication	Date de début	Action prise<br>0 : poursuite 1 : arrêt<br>2: modification<br>3 : ne sait pas	Date de l'action	Après l'action, l'EvIG<br>0 : a disparu 1 : s'est amélioré<br>2 : est inchangé/aggravé<br>3 : inconnu	Causalité<br>0 : non lié au TT<br>1 : lié au TT<br>2 : ne sait pas

5- CONCLUSION INVESTIGATEUR

Au total, selon vous, l'évènement indésirable semble plutôt lié :

☐ à la procédure expérimentale (préciser) : ....

☐ à un médicament concomitant (préciser lequel) : ....

☐ à une autre maladie intercurrente : ....

☐ autre : ....

6- IDENTIFICATION INVESTIGATEUR

Nom - Prénom : ....

Date de déclaration : |__|__| |__|__| |__|__| **Signature :**

Déclaration à faxer au promoteur de l'étude FAX : 05.49.44.30.58
CHU de Poitiers
Rue de la Milétrie BP 577, 86021 Poitiers Cedex

Promoteur
Date de réception promoteur : |__|__| |__|__| |__|__| Numéro Safety Easy : |__|__|__|__| |__|__|__|__|__|

Annexe 5 : Echelle d'évaluation de la performance globale d'une équipe (Oriot 2013)

SIMULATION CHECK-LIST – Médecine/Traumato – V15 (03/11/2012)

Date : Superviseur : Formation : Simulation N° : ....

Team Leader : Aides :

Scénario ***informatique*** utilisé : SimNewB - SimJunior - SimKelly - SimMan

Histoire autour de ce scénario :

Objectifs de la simulation :

Médicotechniques	***Relationnels (CRM)***
1/-	1/-
2/-	2/-
3/-	3/-

SCORE DE CRM (Clinical Teamwork Scale) = SCORE D'ALGORITHME =

SCORE combiné DE PERFOMANCE =

DEBRIEFING Echelles de perception simplifiées : 0 (aucun)-1-2-3-4-5-6-7-8-9-10 (maximal)

Vécu	AVANT		PENDANT	APRES	
	Confiance en soi	*Stress*	*Stress*	*Echec-insatisfaction*	*Réalisme*
**Leader** (médecin senior/junior)					
**Aide** (médecin junior ou IDE)					
**Aide** (IDE/Puéricultrice/SF)					
**Aide** (AS/Auxiliaire puér/ext)					

Analyse	**Médicotechnique / Algorithme**	**CRM / Travail d'équipe / Relationnel**
**Points positifs** (Enoncés par l'équipe lors du débriefing)		
**Points à améliorer** (Repérés par le superviseur pendant la simulation)		

Erreurs humaines : Tunnel, fixation, perte attentionnelle, improvisation

Conclusion

Les messages importants à retenir : énoncés par auto-feedback (« si c'était à refaire, que feriez-vous de différent ? »)

1/- ...
2/- ...
3/- ...
4/- ...

Remarques /suggestions

1-CRM: CLINICAL TEAMWORK SCALE (modifié d'après Guise 2008)

Evaluation globale du travail d'équipe :

Inacceptable	Médiocre	Moyen	Bien	Très bien
0	1 2 3	4 5 6	7 8 9	10

Evaluation détaillée du travail d'équipe :

Leader et aides :

- □ Entre 0 et 10: Identification claire du leader et des aides
- □ Entre 0 et 10: Qualité du rôle de leader
- □ Entre 0 et 10: Rôles ***effectifs*** des aides, entraide

Communication :

- □ Entre 0 et 10: Communication calme, professionnelle
- □ Entre 0 et 10: Utilisation du double-check
- □ Entre 0 et 10: Invitation à rapporter et infos rapportées
- □ Entre 0 et 10: Utilisation du SBAR, I-PASS (nouveaux membres)

Utilisation des ressources :

- □ Entre 0 et 10: Appel d'aide précoce
- □ Entre 0 et 10: Infos (T-MIST, AMPLE, carnet de santé, F de risque)
- □ Entre 0 et 10: Utilisation optimale équipement/examens

Conscience de la situation :

- □ Entre 0 et 10: Anticipation et relevé des constantes
- □ Entre 0 et 10: Résumé verbalisé (penser tout haut)
- □ Entre 0 et 10: Réévaluation/priorisation

Conscience de la famille :

- □ Entre 0 et 10: Information et présence parents/famille

Score TOTAL CTS (maxi = 150)	= ..../.......	= .... /100

2-PREPARATION/INSTALLATION

Score maximal possible =

- □ □ 2 □ 1 □ 0 Vérif matériel: aspi, VMF, O2, IOT, VIO
- □ □ 2 □ 1 □ 0 Scope avant changement de brancard
- □ □ 2 □ 1 □ 0 Déshabillage
- □ □ 2 □ 1 □ 0 Pesée (nourrisson)
- □ □ 2 □ 1 □ 0 Déclenchement du chronomètre
- □ □ 2 □ 1 □ 0 Séchage dos, retrait du champ mouillé, bonnet
- □ □ 2 □ 1 □ 0 Pas de séchage, Sac plastique (prématuré < 33 SA)

3-A – AIRWAY

Score maximal possible =

- □ □ 2 □ 1 □ 0 R. verbale, réactivité (conscience), râles
- □ □ 2 □ 1 □ 0 Position 30° (mise en proclive ou maintien)
- □ □ 2 □ 1 □ 0 Position du cou : neutre, sniffing, extension
- □ □ 2 □ 1 □ 0 Jaw-thrust et chin-lift (± head-tilt)
- □ □ 2 □ 1 □ 0 Bouche : sécrétions, vomissements, CE
- □ □ 2 □ 1 □ 0 Aspiration de la bouche ± nez
- □ □ 2 □ 1 □ 0 Extraction d'un CE visible (finger sweep)
- □ □ 2 □ 1 □ 0 Canule de Guédel (si inconscient)
- □ □ 2 □ 1 □ 0 Tonus, respiration
- □ □ 2 □ 1 □ 0 Laryngoscopie, aspiration bouche ± endotrachéale
- □ □ 2 □ 1 □ 0 *Maintien du rachis cervical dans l'axe*
- □ □ 2 □ 1 □ 0 *Cou : plaie, emphysème, trachée, VJE*
- □ □ 2 □ 1 □ 0 *Retrait/mise d'un collier cervical rigide*
- □ □ 2 □ 1 □ 0 *Oxygène : O2 à 10-15 L/mn par MHC*
- □ □ 2 □ 1 □ 0 *Préparation médicaments pour l'ISR*

4-B – BREATHING

Score maximal possible =

- □ □ 2 □ 1 □ 0 FR, travail, vol pulm (+ abdo), oxygénation
- □ □ 2 □ 1 □ 0 Oxygène : O2 à 0,5-3 L/mn en lunettes
- □ □ 2 □ 1 □ 0 Oxygène : O2 à 10-15 L/mn par MHC
- □ □ 2 □ 1 □ 0 Sonde Gastrique
- □ □ 2 □ 1 □ 0 Aérosols de Salbutamol ± Atrovent*
- □ □ 2 □ 1 □ 0 VMF : 5 VMF de 3'' puis FR = 25-30 (FR=40-60)
- □ □ 2 □ 1 □ 0 Préparation des médicaments pour l'ISR
- □ □ 2 □ 1 □ 0 Manœuvre de Sellick
- □ □ 2 □ 1 □ 0 IOT/INT: laryngoscopie=.......; nb d'essais=.......
- □ □ 2 □ 1 □ 0 Vérification de l'intubation ± FECO2
- □ □ 2 □ 1 □ 0 **ACR: 5 VMF (3''), 2/15 (1/3) ou FR=10**
- □ □ 2 □ 1 □ 0 Stimulations tactiles (dos, plantes des pieds)
- □ □ 2 □ 1 □ 0 Mise en place de la SpO2 (main droite)
- □ □ 2 □ 1 □ 0 Connexion de l'O2 à M .... ; SpO2 = ...
- □ □ 2 □ 1 □ 0 VS-PEP ou VNI
- □ □ 2 □ 1 □ 0 Exsufflation d'un pneumothorax
- □ □ 2 □ 1 □ 0 Drainage thoracique ou préparation
- □ □ 2 □ 1 □ 0 Thyrocricotomie
- □ □ 2 □ 1 □ 0 Surfactant intra-trachéal
- □ □ 2 □ 1 □ 0 Salbutamol : 0,2-1 µg/kg/mn IVSE

5-C – CIRCULATION

Score maximal possible =

- □ □ 2 □ 1 □ 0 FC, peau, pouls, préch (foie, VJE, conj), PA
- □ □ 2 □ 1 □ 0 Douleur thoracique ?
- □ □ 2 □ 1 □ 0 Auscultation cardiaque
- □ □ 2 □ 1 □ 0 ECG (± plusieurs tracés)
- □ □ 2 □ 1 □ 0 VVP (ou VJE)
- □ □ 2 □ 1 □ 0 VIO avec les 5 critères d'évaluation
- □ □ 2 □ 1 □ 0 Surélévation des mbs inférieurs
- □ □ 2 □ 1 □ 0 CVC (fémoral) ou Cathéter Ombilical à 5 cm
- □ □ 2 □ 1 □ 0 Bilan sanguin (avec Dextro* ± Hémocue*)
- □ □ 2 □ 1 □ 0 Exp. Vol. : 20 mL/kg NaCL 0,9% (ou vol. adapté)
- □ □ 2 □ 1 □ 0 Plusieurs exp. volémiques ou transfusions
- □ □ 2 □ 1 □ 0 Commande de sang
- □ □ 2 □ 1 □ 0 Sang O- : 15 mL/Kg + 10 mL/kg NaCL 0,9%
- □ □ 2 □ 1 □ 0 **MCE (2 pouces/mains, 1 main, 2 mains)**
- □ □ 2 □ 1 □ 0 **FC pt RCP=2/15 ou 100 (120)/mn (IOT)**
- □ □ 2 □ 1 □ 0 **Vérification du pouls provoqué**
- □ □ 2 □ 1 □ 0 **Analyse du tracé ECG**
- □ □ 2 □ 1 □ 0 **Adrénaline 10 µg/kg = 0,1 mL/kg IV/IO**
- □ □ 2 □ 1 □ 0 **Causes réversibles 4H/4T (anémie/PNO)**
- □ □ 2 □ 1 □ 0 **Plusieurs doses d'adrénaline/4 mn**
- □ □ 2 □ 1 □ 0 **Défibrillation 4 J/kg**
- □ □ 2 □ 1 □ 0 **Amiodarone (5 mg/kg IVD) ou Lidocaïne**
- □ □ 2 □ 1 □ 0 *Saignement déclive ?*
- □ □ 2 □ 1 □ 0 *Inspection/palpation du cuir chevelu*
- □ □ 2 □ 1 □ 0 *Demande d'un FAST-écho*
- □ □ 2 □ 1 □ 0 *Mobilité bassin ? ± clamp pelvien*
- □ □ 2 □ 1 □ 0 *Déformation cuisse ? ± Donway*
- □ □ 2 □ 1 □ 0 Adrénaline IM/SC

□ □ 2 □ 1 □ 0 Dopamine, Adrénaline, Noradrénaline IVSE
□ □ 2 □ 1 □ 0 Dobutamine IVSE ou Milrinone bolus + IVSE
□ □ 2 □ 1 □ 0 Furosémide : 0,5-1 mg/kg
□ □ 2 □ 1 □ 0 Manœuvres vagales (glace sur le visage)
□ □ 2 □ 1 □ 0 Krénosin (Adénosine): 0,1 mg/kg IVD x 2-3
□ □ 2 □ 1 □ 0 Cardioversion : 1 J/kg
□ □ 2 □ 1 □ 0 Atropine

□ □ 2 □ 1 □ 0 Aspirine IV, Lovenox* IV±SC, Plavix* PO
□ □ 2 □ 1 □ 0 Fibrinolyse : Métalyse* ou Actilyse*
□ □ 2 □ 1 □ 0 CaCL2, HCO3Na, MgSO4, KCL
□ □ 2 □ 1 □ 0 Pace-maker: FC=100; 10-40mA; asynch
□ □ 2 □ 1 □ 0 Sonde de Blakemore
□ □ 2 □ 1 □ 0 Lavages gastriques glacés
□ □ 2 □ 1 □ 0 Prostine

6-D – DISABILITY Score maximal possible =

□ □ 2 □ 1 □ 0 GCS, AVPU, pupilles, mvts aNx, douleur
□ □ 2 □ 1 □ 0 Diazépam IR 0,5 mg/kg ou Midazolam IV 0,1 mg/kg
□ □ 2 □ 1 □ 0 Phénytoïne 20 mg/kg/15 mn IV
□ □ 2 □ 1 □ 0 Phénobarbital 20 mg/kg/15 mn
□ □ 2 □ 1 □ 0 Thiopental 3-5 mg/kg puis IVSE
□ □ 2 □ 1 □ 0 Paracétamol 15 mg/kg IVD (7,5 mg/kg si < 1 an)
□ □ 2 □ 1 □ 0 Pallier II: Codéine, Nalbuphine, Tramadol
□ □ 2 □ 1 □ 0 Pallier III : Morphine, Fentanyl, Sufentanil

□ □ 2 □ 1 □ 0 Tonus, réactivité, fontanelle
□ □ 2 □ 1 □ 0 Naloxone 100 µg/kg IV-IM (2mg si >5A)
□ □ 2 □ 1 □ 0 Flumazénil 30 µg/kg IV
□ □ 2 □ 1 □ 0 Proclive 30°, tête médiane
□ □ 2 □ 1 □ 0 HyperVMF (2 x FR normale) pt 3-5 mn
□ □ 2 □ 1 □ 0 NaCL 20% 1 mL/kg ou 3mL/kg NaCL3% puis IVSE
□ □ 2 □ 1 □ 0 Mannitol 20% 0,5 g/kg IVL
□ □ 2 □ 1 □ 0 Kétamine

7-E – EXPOSURE Score maximal possible =

□ □ 2 □ 1 □ 0 Déshabillage, peau: lésions, purpura, déshydraté
□ □ 2 □ 1 □ 0 Prise de la T°
□ □ 2 □ 1 □ 0 Couvrir et réchauffer
□ □ 2 □ 1 □ 0 Paracétamol 15 mg/kg IVD (7,5 si < 1 an)
□ □ 2 □ 1 □ 0 Dextro et/ou Hémocue*
□ □ 2 □ 1 □ 0 Perf: G5%-G10% polyionique ou NaCL 0,9%
□ □ 2 □ 1 □ 0 Débit de la perf: 421 ou + ou -
□ □ 2 □ 1 □ 0 Recherche d'un empâtement lombaire
□ □ 2 □ 1 □ 0 Recherche d'un globe vésical
□ □ 2 □ 1 □ 0 Sonde vésicale ou poche à urines
□ □ 2 □ 1 □ 0 BU ± prélèvement ECBU, iono urinaire

□ □ 2 □ 1 □ 0 Tt hyperthermie: eau + ventilateur
□ □ 2 □ 1 □ 0 Glace sur la tête, découvert
□ □ 2 □ 1 □ 0 Hypothermie thérapeutique à 33°C
□ □ 2 □ 1 □ 0 Tt hypoglycémie : G10% à 2 mL/kg
□ □ 2 □ 1 □ 0 Glucagon : 0,25 mg/kg IV, IM ou SC
□ □ 2 □ 1 □ 0 ATB : C3G, Amox, Amox-Clav, ± Aminoside
□ □ 2 □ 1 □ 0 Prednisolone 1 mg/kg IVD
□ □ 2 □ 1 □ 0 Hydrocortisone 1 mg/kg (25 mg/m2)
□ □ 2 □ 1 □ 0 (Séro)-Vaccination antitétanique

Score d'algorithme = Points obtenus/maximum de points algorithme possibles

= / = ... /100

En petits caractères → items spécifiques de néo à la naissance

En rouge + italique → items spécifiques de traumato

En encadré → items spécifiques de RCP

<u>Annexe 6</u> : Echelle d'évaluation de la pose d'une voie intra-osseuse IOPAS – *Intra-Osseous Perforamnce Assessment Scale* (Oriot 2012)

**1**	**Installation**	Absence de billot	0
		Billot sous le genou	1
**2**	**Désinfection du site**	Absence de désinfection	0
		Désinfection réalisée	1
**3**	**Gants**	Absence de gants ou gants non stériles	0
		Mise de gants stériles	1
**4**	**Anesthésie locale**	Absence d'anesthésie et patient conscient	0
		Anesthésie locale ou pas d'anesthésie si ACR	1
**5**	**Garde sur l'aiguille**	Absence de garde ou garde > 1,5 cm	0
		Garde de 1 cm avec pouce-index	2
**6**	**Mouvements de rotation**	Absence totale de prono-supination	0
		Prono-supination ébauchée (un à deux mouvements) ou non axiale	1
		Pronation / supination axiale complète	3
**7**	**Aspiration préalable (avec une seringue vide)**	Absence d'aspiration	0
		Aspiration de sang ou de moelle osseuse	2
**8**	**Injection de NaCL 0,9%**	Absence d'injection de NaCL 0,9%	0
		Injection de NaCL 0,9%	2
**9**	**Fixation de la tubulure**	Absence de fixation de la tubulure	0
		Fixation de la tubulure (≥ 2 rappels sur le MI)	1
**10**	**Contention**	Absence de contention	0
		Attelle de jambe (genou + cheville)	1
**11**	**Repérage du site de ponction (après la pose)** (Site = 1,5 cm sous le pôle inférieur de la tibérosité tibiale supérieure au milieu de la face antéro-médiale du tibia)	Hors du site ou aiguille mobile	0
		Au niveau du site ± 0,5 cm	3
**12**	**Angle d'insertion (après la pose)**	Insertion oblique	0
		Insertion perpendiculaire ± 10°	2
**SCORE T O T A L sur 20**			
**SCORE T O T A L sur 100**			

CINQ CRITERES D'EFFICACITE (verbalisés et/ou faits)

**1**	Rupture de la 1ère corticale (sensation de ressaut)	**Oui**	**Non**
**2**	Immobilité dans l'os (aiguille IO solidaire de l'os)	**Oui**	**Non**
**3**	Aspiration de sang ou MO (avec seringue vide de 5 ou 10 ml)	**Oui**	**Non**
**4**	Absence d'extravasation (à l'injection de 2 ml de NaCL 0,9%)	**Oui**	**Non**
**5**	Absence de résistance (à l'injection de 8 ml de NaCL 0,9%)	**Oui**	**Non**
**Enoncés dans l'ordre**		**Oui**	**Non**

CHRONOMETRAGE

	**Heures/minutes**	**Temps en minutes**
**T0** = début installation		néant
**T1** (aiguille IO sur la peau) = *temps d'installation*		
**T2** (tubulure connectée sur VIO)		
**T3** (attelle sur la jambe) = *temps de pose global*		
**T2 – T1** = *temps d'insertion*	Néant	

<u>Annexe 7</u> : Echelle d'évaluation du leadership BAT – *Behavioural Assessment Tool* (Anderson 2010). (Traduction D. Oriot)

Score	0(novice)	1	2(competent)	3	4(expert)
**1-Connaissance de l'environnement**	Désorienté, environnement non familier, ne pose pas de question		L'environnement semble un peu familier; pose quelques questions		Connaît très bien l'environnement; répond aux questions des autres
**2-Anticipation des problèmes**	Non préparé pour le cas; ne demande rien aux autres		Pose 2-3 questions; connaît certains éléments; ne reconnaît pas une situation prévisible		Pose les questions pertinentes; comprend tous les éléments prévisibles de la situation
**3-Identification du rôle de leader**	Ne s'identifie pas clairement comme leader; reste en arrière; manque d'assurance		S'identifie comme leader après question; prend en charge quand demandé de le faire; mauvaise coordination		S'identifie comme responsable du patient et leader de l'équipe; coordonne toutes les activités; inspire confiance
**4-Communication avec les membres de l'équipe**	N'énonce pas clairement les problèmes; voix trop basse ou trop forte; demandes non adressées particulièrement aux acteurs		Identifie correctement les problèmes mais ne communique pas clairement aux autres; voix audible par tous; le plus souvent écoute les interlocuteurs, les nomme et demande de l'aide		Définit les solutions; parle clairement en spécifiant les interlocuteurs; encourage la coopération; écoute les autres; clarifie les propos ambigus
**5-Délégation des tâches**	Essaie de tout faire tout seul; ne reconnaît pas les aides potentielles; ne fait rien ou trop peu quand son aide est demandée; demande trop aux autres sans guidance adéquate		Délègue les tâches de façon appropriée; assigne des tâches mais pas de façon optimale		Assigne clairement des tâches aux aides de façon spécifique; reconnaît quand les aides sont débordés et les assiste ou délègue à quelqu'un d'autre; donne une supervision appropriée
**6-Perception clinique**	Semble préoccupé par les détails mais ne perçoit pas l'essentiel de la situation clinique; ne priorise pas; facilement distrait; incapable de limiter les informations futiles ou le bruit de fond		Reconnaît l'essentiel de la situation clinique et capable de limiter la majorité des détails inutiles; évite les erreurs de fixation		Conscient des détails et organise la PEC globale du patient; ne se distrait pas; priorise correctement; évite les erreurs de fixation
**7-Utilisation de toutes les informations**	De se sert pas des éléments anamnestiques dans l'approche clinique du patient; lent à reconnaître les situations urgentes; ne ré-évalue pas; persiste dans le sens original alors qu'il faut le modifier		Insère des données anamnestiques dans la PEC; ré-évalue souvent; interprète la plupart des éléments cliniques correctement		Insère toutes les données anamnestiques dans la PEC; interprète correctement les données cliniques; ré-évalue en permanence l'état clinique ou quand il existe une discordance; reconnaît rapidement les changements
**8-Utilisation des moyens disponibles**	Ne reconnaît pas les compétences des autres; n'imagine pas des alternatives possibles en cas de manque de matériel ou de personnel		Utilise les compétences des autres la plupart du temps; peine avec le manque de matériel ou de personnel adéquat mais résout finalement à les problèmes		Sollicite et incorpore les compétences des autres; réagit au manque de matériel ou de personnel adéquat en identifiant des solutions alternatives
**9-Reconnaissances des limites/appel d'aide**	N'arrive pas à reconnaître ses propres limites; ne demande aucune aide; tente d'aller au-delà de ses propres compétences avec un risque pour le patient; pas d'appel d'aide		Reconnaît ses limites en connaissance ou habileté mais tarde à demander de l'aide		Reconnaît à temps ses propres limites en connaissances et habileté et demande rapidement de l'aide; appel d'aide demandé de façon appropriée
**10-Comportement professionnel**	Engage des conversations inutiles; commentaires inappropriés; jurons, vulgarité; montre très peu d'intérêt pour le confort du patient; approche brutale de la famille ou inappropriée; devient vite défensif; supervision autoritaire		Garde les conversions inutiles minimales; langage professionnel le plus souvent; considère la famille avec empathie; supervise de façon non autoritaire		Ne s'engage dans aucune conversation inutile; langage et approche toujours professionnels; attitude empathique pour le patient et sa famille; reconnaît le langage non verbal et y répond de façon appropriée; supervise sans jugement
**TOTAL**					

<u>Annexe 8</u> : Echelle clinique du travail en équipe CTS – *Clinical Teamwork Scale* (Guise 2008). (Trad A. Ghazali).

**Global**	Non pertinente	Inacceptable	Médiocre	Moyen	Bien	Parfait
1- Comment évaluez-vous le travail d'équipe au cours de cet accouchement / cette urgence	☐	0	1 2 3	4 5 6	7 8 9	10
**Communication**	Non pertinente	Inacceptable	Médiocre	Moyen	Bien	Parfait
2- Evaluation globale de la communication	☐	0	1 2 3	4 5 6	7 8 9	10
3- Orienter les nouveaux membres (SBAR)	☐	0	1 2 3	4 5 6	7 8 9	10
4- Penser tout haut	☐	0	1 2 3	4 5 6	7 8 9	10
5- Communication directe	☐	0	1 2 3	4 5 6	7 8 9	10
6- Communication en boucle	☐	0	1 2 3	4 5 6	7 8 9	10
**Conscience de la situation**	Non pertinente	Inacceptable	Médiocre	Moyen	Bien	Parfait
7- Evaluation globale de l'état de conscience de la situation	☐	0	1 2 3	4 5 6	7 8 9	10
8- Affectation des ressources	☐	0	1 2 3	4 5 6	7 8 9	10
9- Erreur de fixation	Oui ☐	Non ☐				
**Prise décision**	Non pertinente	Inacceptable	Médiocre	Moyen	Bien	Parfait
10- Evaluation globale de la prise de décision	☐	0	1 2 3	4 5 6	7 8 9	10
11- Priorisation	☐	0	1 2 3	4 5 6	7 8 9	10
**Responsabilité des rôles de leader et des aides**	Non pertinente	Inacceptable	Médiocre	Moyen	Bien	Parfait
12- Evaluation globale des rôles de leader et des aides	☐	0	1 2 3	4 5 6	7 8 9	10
13- Clarté des rôles	☐	0	1 2 3	4 5 6	7 8 9	10
14- Jouer en tant que leader / aide	☐	0	1 2 3	4 5 6	7 8 9	10
**Autre**	Non pertinente	Inacceptable	Médiocre	Moyen	Bien	Parfait
15- Soucieux du patient	☐	0	1 2 3	4 5 6	7 8 9	10
**Score total**						

<u>Annexe 9</u> : Echelle de mesure de l'anxiété (fond anxieux et accès anxieux) STAI – State-Trait Anxiety Inventory) (Spielberger 1983) (Traduction D. Oriot)

<u>Instructions</u> : Lisez chaque phrase et choisissez la réponse appropriée qui indique ce que vous ressentez présentement, c'est-à-dire, à ce moment précis.
Il n'y a pas de bonne ou de mauvaises réponses. Ne passez pas trop de temps sur une phrase particulière, mais donnez la réponse qui semble décrire le mieux vos sentiments présents.

Mettre une croix dans la case qui correspond à votre réponse.

		Pas du tout<br>1	Un peu<br>2	Moyenneme nt<br>3	Beaucoup<br>4
1	Je me sens calme				
2	Je me sens en sécurité				
3	Je me sens tendu(e)				
4	Je me sens contraint(e)				
5	Je me sens à l'aise				
6	Je me préoccupé(e)				
7	Je suis actuellement inquiet(e) en raison de possibles problèmes				
8	Je me sens satisfait(e)				
9	Je me sens apeuré(e)				
10	Je me sens mal à l'aise				
11	J'ai confiance en moi				
12	Je me sens nerveux(se)				
13	Je me sens très anxieux				
14	Je me sens indécis(e)				
15	Je me sens relaxé(e)				
16	Je me sens content(e)				
17	Je suis inquiet(e)				
18	Je me sens perdu(e)				
19	Je me sens assuré(e)				
20	Je me sens affable				
**SCORE TOTAL sur 80**					
**SCORE TOTAL sur 100**					

Annexe 10 : Questionnaire de stress post-événementiel avec l'échelle IES-R – Impact of Event Scale-Revised (Brunet 2003)

Instructions : Voici une liste de difficultés que des personnes peuvent éprouver à la suite d'un événement ayant occasionné un stress aigu. Veuillez indiquer à quel point vous avez été boulversé(e) par chacune de ces difficultés ***au cours des 7 derniers jours*** en ce qui concerne **la dernière séance de simulation**.
Dans quelle mesure avez-vous été affecté(e) ou bouleversé(e) par ces difficultés.

	0	1	2	3	4
	Pas du tout	Un peu	Moyennement	Passablement	Extrêmement
1.Tout rappel de la séance ravivait mes sentiments face à l'événement					
2.Je me réveillais la nuit					
3.Différentes choses m'y faisait penser					
4.Je me sentais irritable et en colère					
5.Quand j'y repensais ou qu'on me le rappelait, j'évitais de me laisser bouleverser					
6.Sans le vouloir, j'y repensais					
7.J'ai eu l'impression que la séance n'était jamais arrivée ou n'était pas réelle					
8.Je me suis tenu loin de ce qui m'y faisait penser					
9.Des images de la séance surgissaient dans ma tête					
10.J'étais nerveux (nerveuse) et je sursautais facilement					
11.J'essayais de ne pas y penser					
12.J'étais conscient(e) d'avoir encore beaucoup d'émotions à propos de la séance, mais je n'y ai pas fait face					
13.Mes sentiments à propos de la séance étaient comme figés					
14.Je me sentais et je réagissais comme si j'étais encore dans la séance					
15.J'avais du mal à m'endormir					
16.J'ai ressenti des vagues de sentiments intenses à propos de la séance					
17.J'ai essayé de l'effacer de ma mémoire					
18.J'avais du mal à me concentrer					
19.Ce qui me rappelait la séance me causait des réactions physiques telles que des sueurs, des difficultés à respirer, des nausées ou des palpitations					
20.J'ai rêvé de la séance					
21.J'étais aux aguets et sur mes gardes					
22.J'ai essayé de ne pas en parler					
SCORE TOTAL sur 88					
SCORE TOTAL sur 100					

<u>Annexe 11</u> : Questionnaire de stress post-événementiel avec l'échelle PCLS – Post-traumatic Check List Scale (Weathers 1993)

<u>Instructions</u> : Voici une liste de difficultés que des personnes peuvent éprouver à la suite d'un événement ayant occasionné un stress aigu. Veuillez indiquer à quel point vous avez été bouleversé(e) par chacune de ces difficultés ***au cours des 30 jours*** qui ont suivi **la dernière séance de simulation**.
Dans quelle mesure avez-vous été affecté(e) ou bouleversé(e) par ces difficultés.

	1	2	3	4	5
	Pas du tout	Un peu	Parfois	Souvent	Très souvent
**Rappel mémoriel**					
**1.Se sentir très perturbé(e) lorsque quelque chose vous rappelle l'événement**					
**2.Etre très perturbé(e) par des souvenirs, des pensées ou des images en relation avec l'événement**					
**Evitement/d'effacement**					
**3.Eviter de penser ou de parler de l'événement ou éviter des sentiments qui sont en relation avec l'événement**					
**4.Eviter des activités ou des situations parce qu'elles rappellent l'événement**					
**Période nocturne**					
**5.Avoir des difficultés pour s'endormir ou rester endormi(e)**					
**6.Etre perturbé(e) par des rêves répétés en relation avec l'événement**					
**Troubles de l'humeur/irritabilité**					
**7.Se sentir énerver ou sursauter facilement**					
**8.Se sentir irritable ou avoir des bouffées de colère**					
**Réactions physiques/hypervigilance**					
**9.Avoir des réactions physiques lorsque quelque chose rappelle l'événement : palpitations, difficultés à respirer, sueurs**					
**10.Etre en état de super alarme, sur la défensive, ou sur ses gardes**					
**Réalité persistante**					
**11.Brusquement agir ou sentir comme si l'événement se reproduisait (comme si vous étiez en train de la revivre)**					
**Fonctions cognitives**					
**12.Avoir du mal à se souvenir des parties importantes de l'événement**					
**13.Avoir des difficultés à se concentrer**					
**14.Se sentir comme si l'avenir était en quelque sorte raccourci**					
**Distanciation/désintérêt**					
**15.Perte d'intérêt dans des activités qui habituellement vous faisaient plaisir**					
**16. Se sentir distant(e) ou coupé(e) des autres personnes**					
**17.Se sentir émotionnellement anesthésié ou incapable d'avoir des sentiments pour ceux qui sont proches de vous**					
**SCORE TOTAL sur 85**					
**SCORE TOTAL sur 100**					

<u>Annexe 12</u> : Stratégies de gestion du stress (Overview of surgical coping strategies) (Wetzel 2010). Traduction A. Ghazali

- Planification et vérification pour éviter des éléments stressants : stratégie de prévention du stress
    - Organiser les interventions
    - Vérifier le matériel
    - Donner des instructions à l'équipe
- Réduction des stress inévitables : stratégie d'anticipation
    - Prendre conscience du stress
    - Se connaître soi-même : ses propres limites de compétences
    - Anticiper les problèmes et réorganiser
    - Reconnaître précocement
    - Accepter les éléments incontrôlables et se concentrer sur les aspects maîtrisables
- Développement de ressources personnelles : avant l'action
    - Se répéter mentalement (l'organisation des interventions)
    - Pratiquer des gestes techniques (entraînement)
    - Elargir ses connaissances
    - Maintenir une bonne condition physique et mentale
    - Se mettre en condition avant l'action (pendant les techniques d'hygiène, lavage des mains)
- Pendant l'action : maîtrise de soi
    - S'arrêter et se reprendre (facilitant la relaxation physique)
    - Se calmer ou rester calme : diminuant ses propres réactions au stress
    - Vérifier les évaluations des autres membres de l'équipe
    - Se parler soi-même
    - Se concentrer
    - Prendre du recul mentalement
    - S'adapter en permanence de façon ergonomique
- Pendant l'action : contrôle de la situation
    - S'arrêter et se reprendre : gagner du temps pour réfléchir et agir
    - Réévaluer et avoir un processus de prise de décision
    - Prioriser, organiser et préparer pendant l'action
    - Communiquer dans l'équipe et assurer un leadership
    - Compenser le manque de soutien de la part de l'équipe : faire le plus d'actes soi-même (assister)
    - Avoir pour chaque étape technique de l'action des procédures systématisées
- Contrôle de l'ensemble des actions et de leur évolution
    - Contrôler la dynamique de l'équipe pendant l'action
    - Monitorer le déroulement de l'action
    - Prêter attention aux activités annexes et se concentrer sur l'objectif
    - Contrôler et maîtriser l'ensemble
