# Supplementary material for: Stress response in the daily lives of simulation repeaters. A randomized controlled trial assessing stress evolution over one year of repetitive immersive simulations
Source: PLoS One. 2019 Jul 25;14(7):e0220111. doi: 10.1371/journal.pone.0220111 (PMC6657860; doi:10.1371/journal.pone.0220111)
Supplement: S2 File — (PDF) [file pone.0220111.s003.pdf]

Randomized controlled trial of multidisciplinary team stress and performance in immersive simulation for management of infant in shock: study protocol

Sim-Stress

Version n°2 - 16/07/2013

Reseach team

SPONSOR
University Hospital of Poitiers - Centre Hospitalier Universitaire de Poitiers
2 rue de la Milétrie
BP 577
86021 POITIERS cedex
Phone: +335.49.44.46 65
Fax: +335.49.44.30.58

PRINCIPAL INVESTIGATOR
Dr Daniel Aiham GHAZALI
Emergency - Medical Ambulance Department
University Hospital of Poitiers - Centre Hospitalier Universitaire de Poitiers
2 rue de la Milétrie
BP 577
86021 POITIERS cedex
Phone: +335 49 44 37 08 (University teaching hospital) /+335 49 45 43 87 (Medical Training and Research Unit)
Fax: +335 49 44 39 01
E-mail: a.d.ghazali@chu-poitiers.fr / aiham@hotmail.com

METHODOLOGIST
Dr Stéphanie RAGOT
CIC - INSERM 0802
Centre Hospitalier Universitaire de Poitiers - University Hospital of Poitiers
2 rue de la Milétrie
BP 577
86021 POITIERS cedex
Phone: +335 49 44 49 13 / Fax: +335 49 44 46 91
E-mail: s.ragot@chu-poitiers.fr

THIS CONFIDENTIAL DOCUMENT IS THE PROPERTY OF THE UNIVERSITY HOSPITAL OF POITIERS

Research study parties

SPONSOR:	University Hospital of Poitiers<br>Centre Hospitalier Universitaire de Poitiers<br>2 rue de la Milétrie-BP 577<br>86 021 Poitiers cedex<br>Phone : 05.49.44.46.65<br>Fax : 05.49.44.30.58
CLINICAL TRIAL PROTOCOL	
TRIAL CODE<br>RCB ID N°	Sim-Stress<br>2013-A00648-37
COMPLETE TITLE<br><br>INDICATION (S) (TARGET)	Effects of stress and simulation on team performance during management of infants in shock<br>Not applicable
PRINCIPAL INVESTIGATOR	Dr Daniel Aiham GHAZALI<br>Emergency - Medical Ambulance Department<br>Centre Hospitalier Universitaire de Poitiers<br>University Hospital of Poitiers<br>2 rue de la Milétrie<br>BP 577<br>86021 POITIERS cedex<br>Phone : 05 49 44 37 08 (University hospital)<br>05 49 45 43 87 (Medical Research Unit)<br>Fax : 05 49 44 39 01<br>E-mail: a.d.ghazali@chu-poitiers.fr
France's Protection Committee of Human Subjects (CPP - "Comité de Protection des Personnes)	Approved on<br>By France's Western Region, Section III, Protection Committee of Human Subjects (Comité de Protection des Personnes ouest III.).
France's National Agency of Medication and Health Product Security ("ANSM - Agence Nationale de Sécurité du Médicament et des produits de santé")	Date of authorisation :<br>Authorisation N°:

THIS CONFIDENTIAL DOCUMENT IS THE PROPERTY OF THE UNIVERSITY HOSPITAL OF POITIERS NO PUBLISHED INFORMATION APPEARING IN THIS DOCUMENT CAN BE REVEALED WITHOUT THE PRELIMINARY WRITTEN CONSENT OF THE UNIVERSITY HOSPITAL OF POITIERS

Table of contents

RESEARCH TEAM 1

RESEARCH STUDY PARTIES 2

TABLE OF CONTENTS 3

SIGNATURES 7

Signature of the investigator 7

RESEARCH STUDY PROTOCOL SUMMARY 8

RESEARCH STUDY PROTOCOL DETAIL 13

1. GENERAL INFORMATION 13
1.1. Title 13
1.2. Sponsor 13
1.2.1. Identity 13
1.2.2. Protocol signatory on the behalf of the sponsor 13
1.2.3. Study director on the behalf of the sponsor 13
1.3. Coordination and study follow-up 13
1.4. Investigators 13
1.4.1. Principal investigator 13
1.4.2. Associated investigators 14
1.5. Vigilance 14
1.6. Data management 14
1.7. Methodologist - biostatistician 15
2. SCIENTIFIC JUSTIFICATION AND GENERAL DESCRIPTION OF THE STUDY 15
2.1. Denomination and description of the clinical situation 15
2.2. Backround information 15
2.3. Teamwork 15
2.4. Stress 16
2.5. Simulation 17
2.6. Summary of benefits, if need be, and predictable risks for those persons lending themselves to the study 17
2.6.1. Benefits 17
2.6.1.1. Individual benefit 17
2.6.1.2. Collective benefit 17
2.6.2. Risks 17
2.6.2.1. Individual risks 17
2.6.2.1.1. Risks and physical constraints 17
2.6.2.2. Collective risk 17
2.6.2.3. Expected individual and general adverse effects 17
2.6.3. Benefit / risk ratio 17
2.7. Statement as to protocol observance, good clinical practices and current legal and statutory measures involved in the

framework of this protocol 18
2.8. Description of the population to be studied 18
2.9. References 19
3. STUDY OBJECTIVES 21
3.1. Primary objective 21
3.2. Secondary objectives 21
4. STUDY DESIGN 21
4.1. Primary outcome measures 21
4.2. Secondary outcome measures 21
4.2.1. Evaluation of performance 21
4.2.2. Evaluation of the effect of repeated simulation sessions on stress and performance 22
4.3. Description of the interventions 22
4.3.1. Initial training of the senior physicians 22
4.3.2. Mannequin and scenarios 22
4.3.3. Stress 22
4.3.4. Supervisors and evaluation 22
4.3.5. Video 22
4.3.6. Debriefing 23
4.3.7. Simulation session repetition 23
4.4. Study schedule 23
4.4.1. Time schedule of measurements 23
4.4.2. Quantification of sessions and various evaluations 24
4.4.2.1. Number of participants included in the study 24
4.4.2.2. Number of team simulation sessions 24
4.4.2.3. Number of individual simulation sessions. 24
4.4.2.4. Number of Holter recordings 24
4.4.2.5. Number of Heart Rate and Blood Pressure measurements 24
4.4.2.6. Number of salivary cortisol concentration measurements 24
4.5. Description of measures taken to reduce or avoid bias 24
4.5.1. Drawing lots 24
4.5.1.1. Drawing lots will constitute representative samples 24
4.5.1.2. Drawing lots for random distribution of each team into one of the 2 of simulation session programs 24
4.5.2. Homogeneity of the study's population 24
4.5.3. Methods of assessment using objective scales 25
4.6. Study duration 25
4.7. Description of rules concerning either definitive or temporary withdrawal 25
4.7.1. Participant withdrawal from this study 25
4.7.2. Partial or complete study interruption 25
4.8. Identification of all data to be collected directly in observation manuals and to be considered as data sources 25
5. INCLUSION AND EXCLUSION CRITERIA 25
5.1. Inclusion criteria 25
5.2. Non-inclusion Criteria 26
5.3. Per-Study Exclusion Criteria (Subject Withdrawals or Discontinuation) 26
5.4. Recruitment modalities 26
5.5. Procedure for exclusion from the study 27
5.5.1. Participant study exclusion criteria and modalities 27
5.5.1.1. Subjects having recalled their assent and having asked to withdraw from the study, will be excluded. 27
5.5.2. Data collection modalities and schedule of withdrawn participants 27
5.5.3. Follow-up modalities of withdrawn participants 27
6. ANALYSIS 27
6.1. Evaluation of the effect of stress on performance 27
6.2. Evaluation of the effect of repeated simulation session 27
7. PERFORMANCE AND STRESS EVALUATIONS 28
7.1. Description of the parameters involved in the evaluation of performance 28
7.2. Description of the parameters involved in the evaluation of stress 28
7.3. Methods and schedule for collection, quantification and analysis of performance and stress evaluation parameters 28
7.3.1. Performance quantification 28
7.3.2. Quantification of stress 28
8. ASSESSMENT OF SAFETY 29
8.1. Evaluation parameters concerning safety 29
8.2. Methods and schedule for collection, quantification and analysis of safety evaluation parameters 29
8.3. Procedures set up for recording and reporting adverse events 29
8.3.1. Characteristics of an adverse event 29

8.3.2. Role of the investigator ... 30
8.3.2.1. Reporting of severe adverse events (SAEs) ... 30
8.3.2.1.1. Information to be passed on to the sponsor ... 30
8.3.2.1.2. Procedures for reporting a severe adverse event to the sponsor ... 30
8.3.2.1.3. Delay period for reporting to the sponsor ... 30
8.3.2.1.4. Time period for reporting to the sponsor ... 30
8.3.2.1.5. Specificities of the protocol ... 31
8.3.2.2. Reporting of non serious adverse events ... 31
8.3.3. Role of the sponsor ... 31
8.3.3.1. Analyze of severe adverse events (SAEs) ... 31
8.3.3.2. Scoring imputability ... 31
8.3.3.3. Reporting Unexpected Serious Side Effects ... 32
8.3.3.4. Annual transmission of safety reports ... 32
8.3.3.5. Reporting other safety data ... 32
8.3.4. *in utero* exposure ... 32
8.3.5. Monitoring Committee ... 32
8.4. Participant follow-up procedures and duration following an adverse event ... 33
9. STATISTICS ... 33
9.1. Description of statistical methods intended for use, including intermediate analyses agenda ... 33
9.1.1. Descriptive analysis ... 33
9.1.2. Evaluation of the effect of stress on performance ... 33
9.1.3. Evaluation of performance score variations ... 33
9.1.4. Comparison of the variation in performance scores between groups 1 and 2 = Evaluation of the effect of repeated simulation on performance ... 33
9.1.5. Comparison of stress between groups 1 and 2 = Evaluation of the effect of repeated simulation on stress ... 33
9.1.6. Inter-observer reproducibility ... 33
9.2. Number of participants to be included in this study and statistical justification ... 34
9.3. Degree of statistical significance ... 34
9.4. Statistical criteria for study termination ... 34
9.5. Method for considering missing, unused or invalid data ... 34
9.6. Management of eventual analysis modifications ... 34
10. RIGHT OF ACCESS TO DATA AND DOCUMENTS SOURCE ... 34
10.1. Access to data ... 34
10.2. Source documents ... 34
10.3. Confidentiality concerning data ... 35
11. CONTROL AND QUALITY ASSURANCE ... 35
11.1.1. Quality assurance of the study safety, progress and data validity. ... 35
12. ETHICAL CONSIDERATIONS ... 35
12.1. Committee of Protection of Human subjects ... 35
12.2. Substantial modifications ... 36
12.3. Information to participants and written consent form ... 36
12.4. Post-study exclusion period from other studies ... 36
12.5. Management related to the study ... 36
12.6. Participant financial compensation ... 36
12.7. Registration in France's national register of persons lending themselves to biomedical research ... 36
13. DATA HANDLING AND CONSERVATION OF DOCUMENTS AND DATA ... 36
13.1. Observation ... 36
13.2. Data capture and processing ... 37
13.3. National Commission for Data Protection and Liberties (CNIL - Commission nationale de l'informatique et des libertés) ... 37
13.4. Filing ... 37
14. INSURANCE ... 37
15. FEASIBILITY OF THE STUDY ... 37
16. PUBLICATION POLICY ... 38
17. PROPERTY - RESULT EXPLOITATION AND VALORISATION ... 38
18. LIST APPENDICES ... 38
18.1. Appendix 1 : Information document given to each research study participant ... 39
After having read this document and been provided with all answers to your questions, and should you agree to participate in this research trial study, we kindly ask you to confirm your decision by signing the consent form enclosed

herewith. You will then keep one original copy of each document. 40
18.2. Appendix 2 : Consent form of the participant in research 41
18.3. Appendix 3: Declaration of Helsinki 42
18.4. Appendix 4: Report form for a serious adverse event 45
18.5. Appendix 5: Evaluation scale of performance of Intra-Osseous access insertion - IOPAS - Intra-Osseous Perforamnce Assessment Scale (Oriot 2012) 47
18.6. Appendix 6: Evaluation Scale of team leadership BAT - Behavioural Assessment Tool (Anderson 2010). (French translation D. Oriot) 48
18.7. Appendix 7: Clinical Scale of the teamwork CTS - Clinical Teamwork Scale (Guise 2008). (French translation A. Ghazali). 49
18.8. Appendix 8 : Anxiety measurement scale (basal and acute anxiety)- STAI - State-Trait Anxiety Inventory) (Spielberger 1983) (French translation D. Oriot) 50
18.9. Appendix 9: Questionnaire of post-event stress with the scale IES-R - Impact of Event Scale-Revised (Brunet 2003)51
18.10. Appendix 10 : PCLS - Post-traumatic Check List Scale (Weathers 1993) 52

SIGNATURES

Signature of the investigator

I have read all the pages of the clinical trial protocol, of which the University Hospital of Poitiers (CHU de Poitiers) is the sponsor. I attest that it contains all the information necessary for the carrying out of this trial. I undertake to realize this trial all in respecting the protocol, terms and conditions that are defined therein. I undertake to realize the trial all in respecting :

the principles of the "Declaration of Helsinki";
both, the International Conference on Harmonisation guidelines and recommendations for Good Clinical Practice (ICH-E6), as well as France's Code of Federal Regulations and guidelines for Good Clinical Practice pertaining to biomedical research concerning medicines for human use (decisions of November 24, 2006);
the French national legislations and the regulations relative to clinical trials;
the conformity with the European Unions' Directive regarding clinical trials on medicinal products for human use [2001/20/EC];
a copy of each, which has been provided to me by the sponsor.

I also commit myself to that the investigators, and all other qualified members of my team, as well as all those involved in the conduct of this study, will have access to copies of this protocol as well as to documents relating to the procedures of this trial, so as to allow them to work all in respecting the dispositions enumerated in these documents.

NAME: Dr Daniel Aiham GHAZALI

Signature: …………………………………………….. Date: ___________________

Signature of the sponsor

Sponsor:

NOM: Mr. Jean-Pierre DEWITTE

Signature: ……………………………………………… Date: ___________________

Research study protocol summary

Title	Randomized controlled trial of multidisciplinary team stress and performance in immersive simulation for management of infant in shock: study protocol
Sponsor	University Hospital of Poitiers<br>Centre Hospitalier Universitaire de Poitiers<br>2 rue de la Milétrie - BP 577<br>86021 Poitiers cedex<br>Telephone : 05.49.44.46.65<br>Fax : 05.49.44.30.58
Investigator	University Hospital of Poitiers<br>Centre Hospitalier Universitaire de Poitiers<br>2 rue de la Milétrie - BP 577<br>86021 Poitiers cedex<br>Telephone : : 05 49 45 37 08 / Fax: 05 49 44 39 01<br>email: a.d.ghazali@chu-poitiers.fr
Justification / context	Patients' security depends on the efficiency of technical skills, the respect of algorithms and the quality of teamwork. Stress modifies all these parameters. We shall study, in simulation conditions, the multi-disciplinary management of an infant in a state of shock, requiring an intraosseous access, as well as the effect of repeated simulations in a climate of stress.
Primary objective	***To seek evidence for the existence of stress,*** as it will be assessed by three processes throughout various scenarios:<br>Biological stress (Salivary Cortisol Concentration [SC]);<br>Electrophysiological stress (24h-Holter, punctual measures);<br>Psychological stress.
Secondary objectives	***To evaluate performance***, as it will be assessed by three processes:<br>Team performance (actions, algorithm, and treatment);<br>IO access insertion;<br>Teamwork and leadership.<br>To evaluate the effect of repeated simulation sessions on performance and stress:<br>Performance will be evaluated using the same assessment tools;<br>Stress will be investigated by studying the variations of given stress markers and coping responses.

Primary outcome measures	Evaluating evidence for the existence of stress:<br>***Biological stress:*** SC will be measured by an Enzyme-Linked Immunosorbent Assay (ELISA) kit (IBL international®, Hamburg, Germany). SC is commonly used to assess stress (Wagner 2010, Beko 2010, Dovio 2010) and this method has been used in simulation (Bong 2010). It will be assessed one day prior to the simulation, just before and right after the simulation session, as well as following debriefing.<br>***Electrophysiological stress:*** Holter parameters (HR analysis, temporal and spectral analysis with PNN50 [Wilhelm 2005] as well as LF/HF ratio) will be obtained using the software Synscope* (Sorin Group) throughout an entire day (24h recording) - during the resting phase, i.e., sleep, during selective periods at the time of SC sampling, as well as during the phases simulation, debriefing, and pauses following simulation (Task Force 1996). Timely measures of HR and BP will be associated with this analysis.<br>***Psychological stress:*** will be assessed during the simulation by self-evaluation (STAI). Participants will be placed in an isolated room of the simulation center so as not to be disturbed.<br>PTSD questionnaires will be emailed to all participants.<br>***Self-assessment*** using STAI - *State-Trait Anxiety Inventory* (Spielberger 1983) just before and right after the simulation session, as well as following debriefing. SOM self-rating scale of stress<br>***Early Post-trauma stress disorder*** will be determined on the 7th day following simulation, using IES-R - *Impact of Event Scale-Revised* (Brunet 2003)<br>***Late Post-trauma stress disorder*** will be determined after one month following simulation, using PCLS - *Post- traumatic Check-List Scale* (Weathers 1993).
Secondary outcome measures	Evaluation of performance:<br>***Global performance***: using a performance assessment scale ("TAPAS"), designed by the Simulation Laboratory, and in validating process (Oriot 2013)<br>***IO access procedure process and success***: insertion technique as evaluated by an assessment scale, time to decision, and duration of procedure (Oriot 2012)<br>Teamwork performance scales:<br>**Leadership assessment:** BAT – *Behavioural Assessment Tool* (Anderson 2010)<br>**Teamwork assessment:** CTS - *Clinical Teamwork Scale*, CTS (Guise 2008)<br>Effect of repeated simulation sessions on stress and performance will be assessed by the same stress parameters and team performance scores
Methodology / Study Design	Interventional Biomedical research with bodily fluid samples obtained from healthy volunteers. Monocentric study, using recruitment that will be regional, prospective, randomized and controlled.<br>Twelve teams will be randomized into two equal groups, 1 and 2. Each team will be composed of 4 persons (1 senior physician, 1 resident, 1 nurse, 1 ambulance driver), all of whom will have received in advance the same theoretical and practical education as concerns IO access. Group 1 will undergo 9 simulations in 12 months whereas group 2, only 3.

Subject Inclusion Criteria	All participants in the trial study will be volunteers;<br>Age ≥ 18;<br>All participants will be legally free and not subject to any custody, guardianship or tutelage measures;<br>Participants should all adhere to France's Social Security Health regime (Sécurité Sociale);<br>Free, prior, clearly informed and written consent;<br>Signed agreement allowing for video-recordings that will be strictly and exclusively used for evaluation purposes during this study;<br>Four-member teams:<br>1/- Emergency physician of the Poitou-Charentes region and its surroundings, having professional experience of less than seven years and having obtained a Complementary University degree in Pediatric Emergency Procedures (Diplôme Universitaire des Gestes d'Urgence en Pédiatrie) during the last 3 years, which abides by the latest recommendations of advanced pediatric resuscitation, established in 2010 by the AHA - *AMERICAN HEART ASSOCIATION* (Kleinman, 2010) and the ERC - *EUROPEAN RESUSCITATION COUNCIL* (Barient, 2010);<br>2/ Medical Resident ("interne") currently registered in a Complementary Specialized Diploma of Emergency Medicine ("DESC - *Diplôme d'Études Spécialisées Complémentaires* - de Médecine d'Urgence") at the University of Poitiers, and having received training in pediatric emergency procedures.<br>3/ Registered Nurse of the Emergency Medical Sustem and Emergency Medical and Resuscitation Mobile Services ("SAMU - *Service d'Aide Médicale d'Ugence*" SMUR - " *Service Mobile d'Urgence et de Réanimation*") of the University Hospital of Poitiers (CHU de Poitiers), having a professional experience of less than seven years, and having obtained a EPILS - *European Pediatric Immediate Life Support* - diploma during the last two years;<br>4/-Ambulance driver of the Emergency Medical System and Emergency Medical and Resuscitation Mobile Services ("SAMU - *Service d'Aide Médicale d'Ugence*", SMUR - " *Service Mobile d'Urgence et de Réanimation*") of the University Hospital of Poitiers (CHU de Poitiers), and having a professional experience of less than seven years.
Subject Exclusion Criteria	- Age < 18 years<br>- Current participation in another clinical research study, with an exclusion period of at least a month between the two research protocols;<br>- Participants not benefiting from a Social Security Health Regime ("Sécurité Sociale");<br>- Pregnant or breast-feeding women, women at an age to procreate and not using an effective contraception method (either hormonal / mechanical [*per os*, injectable, transcutaneous, implantable, intra-uterine device] or surgical [tubal ligation, hysterectomy, total ovariectomy];<br>- Medical and/or psychiatric antecedents that could influence the state of stress.<br>- Cardiac or convulsive neurological antecedents.<br>- Pacemaker or automatic internal cardioverter-defibrillator bearers;<br>- Participants using cardiotropic agents and/or beta-2 mimetic bronchodilator medication (possible modification of cardiac electrophysiolocal parameters).
Per-Study Exclusion Criteria (Subject Withdrawals or Discontinuation)	- Disregard of evaluation dates and schedules that could thus either compromise the simulation sessions or influence the parameters being evaluated.<br>- Exclusion of the entire team in case of withdrawal of one of its members.

Treatments / Strategies / Procedures	Two randomized groups, 1 and 2, of 6 teams each. Each team will consist of 4 persons (1 senior physician, 1 resident, 1 nurse, and 1 ambulance drive), all of whom will be exposed to simulated multidisciplinary management of an infant in shock (among a predefined data base of scenarios), requiring IO access.<br>Group 1: 9 simulation sessions within 12 months;<br>Group 2: 3 simulation sessions within 12 months.
Number of SUBJECTS	48
Study Duration	Estimated Time to Complete Volunteer Subject Enrolment: one year<br>Volunteer Subject Participation Duration: one year<br>Total study duration: Two years<br>Presumed study start date: May, 2013<br>Presumed study end date: May, 2015
Expected results	Stress decreases team performance, and repeated simulation within a climate of stress allows maintaining high performance levels.

Research study protocol detail

1. GENERAL INFORMATION

1.1. Title

Effects of stress and simulation on team performance during management of an infant in shock

1.2. Sponsor

1.2.1.Identity

University Hospital of Poitiers
Centre Hospitalier Universitaire de Poitiers
2 rue de la Milétrie-BP 577
86 021 Poitiers cedex
Telephone: +335 49 44 46.65
Fax: +335 49 44 30 58

1.2.2.Protocol signatory on the behalf of the sponsor

Jean-Pierre DEWITTE, General Director
University Hospital of Poitiers
Centre Hospitalier Universitaire de Poitiers
2, Street of Milétrie - BP 577
86021 Poitiers cedex (postal code for corporate users)
Phone: +335 49 44 39 29
Fax: +335 49 44 39 80

1.2.3.Study director on the behalf of the sponsor

Research Director
University Hospital of Poitiers
Centre Hospitalier Universitaire de Poitiers
2 rue de la Milétrie-BP 577
86 021 Poitiers cedex
Telephone: +335 49 44 46 65
Fax: +335 49 44 30 58

1.3. Coordination and study follow-up

Dr Daniel Aiham GHAZALI
University Hospital of Poitiers
Centre Hospitalier Universitaire de Poitiers
Emergency Department and Emergency Medical and Resuscitation Mobile Services
Urgences-SAMU86

Accompanied by a Clinical research associate, as designated by the sponsor.

1.4. Investigators

1.4.1.Principal investigator

Dr Daniel Aiham GHAZALI
University Hospital of Poitiers
Centre Hospitalier Universitaire de Poitiers
Emergency Department and Emergency Medical and Resuscitation Mobile Services
Urgences-SAMU86
Phone: +335 49 45 43 87/ Fax: +335 49 44 39 01
E-mail: a.d.ghazali@chu-poitiers.fr

1.4.2.Associated investigators

Prof. Denis ORIOT
University Hospital of Poitiers
Centre Hospitalier Universitaire de Poitiers
Pediatric emergency department, Medical and Surgical services
2 rue de la Milétrie- BP 577 - 86021 Poitiers cedex
Tél: +335 49 44 61 71/ Fax : +335 49 44 37 59
E-mail: d.oriot@chu-poitiers.fr; denis.oriot@gmail.com

Prof. Michel SCEPI
University Hospital of Poitiers
Centre Hospitalier Universitaire de Poitiers
Emergency Department and Emergency Medical and Resuscitation Mobile Services
Urgences-SAMU86
Telephone: +335 49 44 40 88/ Fax: +335 49 44 39 01
E-mail: d.oriot@chu-poitiers.fr; denis.oriot@gmail.com

Dr Christine MILLET
University Hospital of Poitiers
Centre Hospitalier Universitaire de Poitiers
Biophysics Laboratory - Laboratoire de Biophysique
2 rue de la Milétrie- BP 577 - 86021 Poitiers cedex
Telephone : +335 49 44 49 59/ Fax : +335 49 44 40 58
E-mail : c.millet@chu-poitiers.fr

Dr Philippe SOSNER
University Hospital of Poitiers
Centre Hospitalier Universitaire de Poitiers
Cardilogy Department - Service de Cardiologie
2 rue de la Milétrie- BP 577 - 86021 Poitiers cedex
Phone : +335 49 44 48 13/ Fax : +335 49 44 48 14
E-mail : philippe.sosner@chu-poitiers.fr

Dr Jean-Jacques CHAVAGNAT
Henri Laborit Hospital Centre
Health services and medical logistics pole
Pierre Janet Pavilion
Pôle de santé publique et de logistique médicale
Pavillon Pierre Janet
370 avenue Jacques Cœur - 86021 Poitiers cedex
Phone : +335 49 44 58 13/ Fax : +335 49 44 58 14
E-mail : Jean-jacques.chavagnat@ch-poitiers.fr

1.5. Vigilance

Dr Sophie DURANTON
University Hospital of Poitiers
Direction of Clinical Research
2 rue de la Milétrie - BP 577 - 86 021 Poitiers cedex
Telephone: +335 49 44 30 50/Fax : +335 49 44 30 58
E-mail: sophie.duranton@chu-poitiers.fr

1.6. Data management

Data Management and Bibliometry Unit
Direction of research
University Hospital of Poitiers
Centre Hospitalier Universitaire de Poitiers
2 rue de la Milétrie - BP 577 - 86021 Poitiers cedex

University Hospital of Poitiers - CHU de Poitiers Sim-Stress
Telephone: +335 49 44 30 08/ Fax: +335 49 44 30 58
E-mail: sophie.duranton@chu-poitiers.fr

1.7. Methodologist - biostatistician

Dr Stéphanie RAGOT
CIC - INSERM 0802
University Hospital of Poitiers
Centre Hospitalier Universitaire de Poitiers
2 rue de la Milétrie - BP 577 - 86021 Poitiers cedex
Tél: +335 49 44 46 89/ Fax : +335 49 44 46 91
Telehone: +335 49 44 46 89/ Fax : +335 49 44 46 91
E-mail: s.ragot@chu-poitiers.fr; stephanie.ragot@univ-poitiers.fr

2. SCIENTIFIC JUSTIFICATION AND GENERAL DESCRIPTION OF THE STUDY

2.1. Denomination and description of the clinical situation

We shall set an "infant in shock" as a model for professional stress during management by a multidisciplinary team.

2.2. Backround information

An infant of six months, held in the arms of his parents, was admitted into an acute, level one (high priority) emergency room ("salle d'accueil des urgences vitales [SAUV]"), for hypovolemic shock following dehydration, in a context of acute gastro-enteritis. The medical team, of which the leader was a young doctor, was facing tremendous stress due to the impossibility to establish a peripheral venous access despite numerous attempts. The insertion of an intraosseous access (IO), recommended procedure in this context (Biarent 2010), was not evoked by lack of competence in this procedure. What was there to do? The lethal prognosis of this child was overturned following reinforced assistance by a physician trained in the practice of IO access, which allowed to establish a rapid volume expansion, and thus ensure the treatment of this type of shock.

How can one guarantee the security of such an infant in shock after being admitted into a level one (high priority) emergency room? This clinical case well reflects the three elements that are involved in the safety of a patient: the mastery of a technical skill (success rate or efficiency), such as IO access performance (Oriot 1994), the respect for algorithms (effectiveness) (Biarent on 2010) and the quality of teamwork (Eppich 2008). Performance is the resultant of these elements, all indispensable so as to obtain maximal security for the patient.

Management of an infant in life-threatening distress corresponds to a complex clinical situation where the main objective is to coordinate and accomplish numerous procedures, which involve several actors, who will proceed according to a pre-established algorithm, determined by international recommendations. Performance, that is, the global quality of management, depends not only on actions taken according to an algorithm but, also, on the quality of teamwork necessary to best coordinate management of the patient (Rosen 2008). Stress, generated by the fact that the situation evolves an infant in life-threatening distress, is a factor that can modify all these parameters - control of mastered technical skills, respect of algorithms and coordinated teamwork - and, by consequence, affect global performance in management of such a case.

2.3. Teamwork

One of the constituent characteristics of a team is that its members are required to interact with each other so as to successfully realize a team task (Beaubien 2004). Teamwork becomes a crucial element for effective patient care. Unfortunately, only few courses have focused on training towards effective teamwork (Shapiro 2008). Education targeting teamwork can establish a more collaborative working style within a group of clinicians, so as to improve patient safety (Morey 2002). Functioning within a team imposes technical competencies such as distribution of tasks, control of communication, awareness of the situation and decision-making within a team (Yee 2005). Research in the field of non-technical skills led towards the development of scales evaluating behavior and communication. Among the existing tools, the CTS - Clinical Teamwork Scale - was used and validated in emergency situation simulations, and showed excellent interobserver reproducibility, accounted for by an intra-class coefficient of correlation = 0.98 (Way 2008).

Furthermore, strong and effective team leadership is absolutely necessary to attain higher performance levels, because team leaders have a triple role: to develop team spirit, to resolve problems they are presented with, and, finally, to motivate teamwork. Competencies required to be a leader consist of a clear perception of team dynamics,

an understanding of personal interactions impact, and the importance of communication on team function (Shapiro 2008). Teaching leadership is highly insufficient for the time being, and deserves to be further developed so as to attain a degree of excellence. Evaluation of leadership is complex - in simulation, a frequently used scale is that of BAT - Behavioural Assessment Tool (Anderson 2010).

2.4. Stress

The effect of stress on a medical team during emergency management of patients must be considered, in that it compromises the security of patients (Gaba 2002). Stress covers all the biologic, electro-physiological and psychological disturbances that may be provoked by an aggression of some kind on the body. Stress provokes an emotional response - in particular, acute anxiety - as well as an alteration of cognitive processes (Kirschbaum 1996, Wolf 2003). The aeronautical industry has long ago admitted to the effects of stress on performance, with risks of decreased performance and human error (Kohn 2000, Loewenthal 2000). Performance losses were also reported in military and sports domains (Driskell 1991, Anshel 2002, Wallenius 2004). Stress decreases recognition of errors, by loss of continuous attention. It increases unsuitable answers during critical management (Sexton 2000). On the other hand, recognizing errors and factors of stress, associated to simulation-based training, improves aeronautical team performance and thereby increases passenger security (Sexton 2000).

Stress has an important psychological effect. Post-traumatic stress state (PTSS) is a frequent pathology in clinical psychiatry, and in persons having undergone one (or more) traumatic event(s) during their lifetime (Yao 2003). One of the scales for evaluating the impact of post-traumatic stress one month later is the Post-traumatic Check List Scale (PCLS), which allows for a reliable approach of PTSS by auto-evaluation (Weathers 1993). There are other validated stress evaluation scales in English, translated and validated in French, such as the IES-R - Impact of Events Scale-Revised, very widely used by and recognized at an international level (Brunet 2003). The latter was created by Weiss and Marmar in 1996, based on Horowitz’s Impact of Event Scale (Horowitz 1979), which was translated and validated in French by Brunet in 2003 (Laurent 2007). IES-R measures the presence of traumatic stress connected to recent, traumatizing events. The initial version of Horowitz's scale consisted of two axes: avoidance and repetition behaviour associated to traumatic symptomatology. In 1996, Weiss and Marmar added a third dimension: neurovegetative hyperactivity. Twenty two items thus evaluate the intensity of each symptom, using a Likert type scale ranging from "extremely" to "not at all" (Laurent 2007). The IES-R scale was validated by using numerous accident or aggression victims. It differentiates well subjects in a state of acute stress from presenting post-traumatic stress (Brunet 2003). A score of 22 would be indicative of an acute stress whereas a score of 36 would suggest the presence of PTSS, without, however, providing any diagnosis (Brunet 2003).

Professionals subjected to acute stress causes them to implement various stress management strategies (Anshel 2002). It was demonstrated that bad stress management has negative effects, notably in performance (Hassan 2006). It was recently reported in simulation studies, that the management of surgeons' stress was independent from any professional experience (Wetzel 2010). This particular team used an interview method of those involved, following simulation, so as to determine which of 6 predefined stress management strategies were applied.

Stress induces biologic modifications such as the increase of cortisol in the body. Just as blood plasma cortisol, so salivary cortisol is a faithful reflection of stress, with the major advantage that it does not require invasive sampling techniques (Weibel 2003). Salivary cortisol is a good biologic marker of acute stress, and has been measured in a number of stress models (Schreinicke 1990, Wagner 2010). It is frequently used to monitor athletes: it is more elevated during or right after a competition, as compared to training sessions, regardless of the athlete's competition level (Maso 2002). This rise in salivary cortisol seems to be connected to physiological and psychological stress during a competition (Filaire 1997). Also, in models of long-term stress, such that of being exposed to road traffic noise (Wagner on 2010) or plane motion (Berthon 2001), it has been reported that there is a significant increase salivary cortisol concentrations.

Stress is also at the source of physiological disturbances, such as rise in arterial blood pressure, acceleration of heart rate and modification in its variability and electrocardiographic RR' interval. A number of variables can be measured by Holter and indicate the presence of stress: blood pressure, average heart rate (HR) and its variation, variation in statistical standard deviation of average HR, percentage of RR' intervals > 500 ms, Variability Index (percentage of the average difference between two successive RR' intervals), combined indices such as pNN50, ASDRR'/5 (measurements of RR' using mean standard deviations on 5 minute intervals) and the SDARR '/5 (standard deviations of average RR' on 5 minute intervals) (Anonymous 1996, Task Force 1996). Analysis of the RR' interval is an effective non-invasive method to estimate the autonomous nervous system under conditions of stress (Zheng 1997). A correlation has been established between the state of stress and a decrease of the RR' interval (Sloan 1994). The global effect of stress, confirmed by an increase of salivary cortisol, consists of an increase in blood pressure and a decrease in the RR' interval (Lucini 2002). This marker was also used to assess the effect of stress on individuals with no cardiovascular pathology, and its causation of increased arterial blood pressure, such as what would occur in everyday real life stress conditions (Lucini 2005). Furthermore, the RR' interval has also been correlated with the effect of the stress in individuals with no cardiovascular pathology, but responsible for an increase in blood pressure, such as what would occur in everyday real life stress conditions

(Lucini 2005), or in per-anesthesia states, allowing for assessment, *a contrario*, of the depth of sedation (Nishiyama 2010).

In medicine, the effect of stress on professionals has until now been underestimated. Self-assessment of stress can be evaluated by the Stress-O-Meter (SOM) score (Olpin 2010). Some even deny any errors committed under the effect of the stress, during patient management, in contrast to aviation contexts (Sexton 2000). Certain authors were able to prove a state of post-traumatic stress in Emergency Medical Assistance (SAMU) personnel in France (Laurent 2007, Vaiva 2008), notably using the IES-R scale (Horowitz 1979, Brunet 2003, Craemer 2003). Quite recently, it was demonstrated, in simulation, that stress (assessed by a questionnaire, heart rate variability and salivary cortisol concentrations) altered surgical performance, and was a key element in the quality of care (Wetzel 2010). This team used a questionnaire for a self-assessment of stress, so as to analyze underlying anxiety and anxiety induced by the situation: the STAI - State-Trait Anxiety Inventory, elaborated by Spielberger (Spielberger 1983).

2.5. Simulation

Medical knowledge does not restrict itself to theoretical knowledge, as exhaustive as it may be, it involves, as well, practical know-how, represented by the performance of medical - technical procedures, allowing one to acquire and develop competency and way of being with the surrounding medical team, patients and families. Simulation is an educational tool that allows such learning. Medical simulation is clearly organized in the emergency treatment educational programs of North America (Issenberg 1999, Issenberg 2008). Indeed, in 2009, this learning method has even been recognized by The American Senate (The Enhancing SIMULATION Act of 2009), as the standard method of learning of emergency care (www.medsim.org, Forbes and Kennedy 2009). In order to attain a high level of performance, the aptitudes (competence) of everyone involved must be improved. Simulation allows one to attain such an educational objective, by a learning *ex-vivo* (in complete safe conditions for the patient and the operator), and to provide educational evaluations as to the quality of various acquired practical aptitudes (competencies). Even though training on a simulator allows one to reduce errors, it is also more effective to associate entire teams to simulation training (Blum 2004, Birnbach 2008). Simulation allows to improve teamwork, notably in adult (Shapiro 2004) and pediatric (Gilfoyle 2007, Eppich 2008) emergency services.

2.6. Summary of benefits, if need be, and predictable risks for those persons lending themselves to the study

2.6.1.Benefits

2.6.1.1. Individual benefit

The benefit for any given participant in this research study would be to receive, free of any charge, an education by simulation - an innovative practical education, evaluated on medical and relational difficulties faced during management of complex pediatric cases.

2.6.1.2. Collective benefit

Collective profit consists of simulation training of health professionals of the University Hospital of Poitiers and other hospital centers of Poitou-Charentes region in France.

2.6.2.Risks

2.6.2.1. Individual risks

2.6.2.1.1. Risks and physical constraints

Risks related to the participation are those of the discomfort caused by the bearing of a Holter during 24 hours, and the sampling of three salivary samples.

2.6.2.2. Collective risk

None.

2.6.2.3. Expected individual and general adverse effects

None.

2.6.3.Benefit / risk ratio

This study offers a very positive benefit / risks ratio.

2.7. Statement as to protocol observance, good clinical practices and current legal and statutory measures involved in the framework of this protocol

The sponsor and the investigator also commit themselves to that this study will proceed:

- According to the protocol,
- According to France's best clinical practices guidelines and recommendations,
- According to current legal and statutory measures in France.

2.8. Description of the population to be studied

We shall study multidisciplinary teams consisting of four members each: a senior physician, a junior doctor (resident), a nurse and an ambulance driver.

For each of these statuses, an exhaustive list will be established and will serve for probing. Participants drawn at random will be contacted by e-mail so as to be informed of the study, and to invite them, so as to ask for their consent to participate. (The definitive, signed agreement will be obtained the day before the first session, in the presence of the principal investigator of this study). Modalities as to the drawing of lots are described in paragraph 4.5.1.

2.9. References

Advanced Initiatives in medical simulation. Available at http://www.medsim.org/documents/H.R.855.pdf Accessed March 17, 2009

Anderson JDM, Meckler G, Boyle K, LeFlore J, Warre J. How to use the Behavioral Assessment Tool: assessment of CRM-based behavioral skills in simulation using a validated, reliable tool. Available at http://www.ipssw2010.com/img/IPSSW2010%20Programme%20final.pdf Accessed March 24, 2011

Anonymous. Heart rate variability. Standards of measurement, physiological interpretation, and clinical use. Task Force of the European Society of Cardiology and the North American Society of Pacing and Electrophysiology. Eur Heart J. 1996;17:354-81

Anshel MH, Anderson DI. Coping with acute stress in sport: Linking athletes' coping style, coping strategies, affect, and motor performance. Anxiety Stress Coping Int J 2002;15:193-209

Beaubien JM, Baker DP. The use of simulation for training teamwork skills health care: how low can you go? QualSaf Health Care 2004;13:i51-6

Beko G, Varga I, Glaz E, Sereg M, Feldman K, Toth M et al. Cutoff values of midnight salivary cortisol for the diagnosis of overt hypercortisolism are highly influenced by methods. Clinica Chimica Acta 2010;411:364-7

Berthon P, Lac G. Etude des réactions du cortisol salivaire au déplacement en avion pour une équipe de rugby. Science et Sports 2001;16:45-7

Biarent D, Bingham R, Eich C, López-Herce J, Maconochie I, Rodríguez-Núnez A et al. European Resuscitation Council Guidelines for Resuscitation 2010. Section 6. Paediatric life support. Resuscitation 2010;81:1364-88

Birnbach DJ, Salas E. Can medical simulation and team training reduce errors in labor and delivery? Anesthesiol Clin 2008;26:159-68

Blum RH, Reamer DB, Carroll JS, Sunder N, Felstein DM, Cooper JB. Crisis resource management training for an anaesthesia faculty: a new approach to continuing education. Med Educ 2004;38:45-55

Bong CL, Lightdale JR, Fredette ME, Weinstock P. Effects of simulation versus traditional tutorial-based training on physiological stress levels among clinicians: a pilot study. Sim Healthcare 2010;5:272-8

Brunet A, Saint-Hilaire A, Jehel L, King S. Validation of a French version of the impact of Event Scale-Revised. Can J Psychiatry 2003;48:56-61

Creamer M, Bell R, Failla S. Psychometric properties of the Impact of Event Scale-Revised. Behav Res Ther 2003;41:1489-96

DASH. www.harvardmedsim.org/dash.html

Dovio A, Roveda E, Sciolla C, Montaruli A, Raffaelli A, Saba A et al. Intense physical exercise increases systemic 11beta-hydroxysteroid dehydrogenase type 1 activity in healthy adult subjects. Eur J Appl Physiol 2010;108:681-7

Driskell JE, Salas E. Overcoming the effects of stress on military performance: Human factors, training, and selection strategies. In: Gal R, Mangelsdorff D, eds, Handbook of military psychology, John Wiley and Sons Ed, Oxford 1991:183-93

Eppich WJ, Brannen M, Hunt FA. Team training: implications for emergency and critical care paediatrics. Curr Opin Pediatr 2008;20:255-60

Filaire E, Duché P, Robert A, Lac G. Influence d'une compétition officielle et d'une session d'entraînements sur les concentrations de cortisol salivaire. Science et Sports 1997;12:66-71

Forbes and Kennedy. HR 855 - A Bill to amend the Public Health Service Act to authorize medical simulation enhancement programs, and for other purposes. February 4, 2009

Gaba DM, Howard SK. Patient safety: fatigue among clinicians and the safety of patients. N Engl J Med 2002;347:1249-55

Gilfoyle E, Gottesman R, Razack S. Development of a leadership skills workshop in paediatric advanced resuscitation. Med Teach 2007;29:276-83

Guise JM, Deering SH, Kanki BG, Osterweil P, Li H, Mori M et al. Validation of a tool to measure and promote clinical teamwork. Simul Healthc 2008;3:217-23

Hassan I, Weyers P, Maschuw K et al. Negative stress-coping strategies among novices in surgery correlate with poor virtual laparoscopic performance. Br J Surg 2006;93:1554-9

Horowitz M, Wilner N, Alvarez W. Impact of Event Scale: a measure of subjective stress. Psychosom Med 1979;41:209-18

Issenberg SB, McGaghie WC, Hart IR, Mayer JW, Felner JM, Petrusa ER et al. Simulation technology for health care professionals skills training and assessment. JAMA 1999;282:861-6

Issenberg SB, Scalese RJ. Simulation in health care education. Perspect Biol Med 2008;51:31-46

Kleinman ME, Chameides L, Schexnayder SM, Samson RA, Hazinski MF, Atkins DL et al. American Heart Association Guidelines for Cardiopulmonary American Heart Association Guidelines for Cardiopulmonary 2010. Part 14: Pediatric Advanced Life Support. Circulation. 2010;122:S876-S908

Kirschbaum C, Wolf OT, May M et al. Stress and treatment-induced elevations of cortisol levels associated with impaired declarative memory in healthy adults. Life Sci1996;58:1475-83

Kohn LT, Corrigan J, Donaldson MS, Committee on Quality of health Care in America. To err is human: building a safer health system. National Academy Press Ed, Washington DC 2000

Laurent A, Chahraoui K, Carli P. Les répercussions psychologiques des interventions médicales urgentes sur le personnel SAMU. Etude portant sur 50 intervenants SAMU. Annales Médico Psychologiques 2007;165:570-8

Loewenthal KM, Eysenck M, Harris D et al. Stress, distress and air traffic incidents: Job dysfunction and distress in airline pilots in relation to contextually-assessed stress. Stress Med 2000;16:179-83
Lucini D, Norbiato G, Clerci M, Pagani M. Hemodynamic and autonomic adjustments to real life stress conditions in humans. Hypertension 2002;39:184-8
Lucini D, Di Fede G, Parati G, Pagani M. Impact of chronic psychosocial stress on autonomic cardiovascular regulation in otherwise healthy subjects. Hypertension 2005;46:1201-6
Maso F, Cazorla G, Godemet M, Michaux O, Lac G, Robert A. Influence d'une compétition de rugby sur le taux de cortisol salivaire. Science et Sports 2002;17:302-5
Morey JC, Simon R, Jay DG, Wears RL, Salisbury M, Dukes KA et al. Error reduction and performance improvement in the emergency department through formal teamwork training: evaluation results of the Med Teams project. Health Serv Res 2002;37:1553-81
Nishiyama T. Recent advance in patient monitoring. Korean J Anesthesiol 2010;59:144-59
Olpin M, Hesson M. Stress Management for Life: A Research-Based, Experimental Approach. 2nd ed. Belmont, CA: Wadsworth, Cengage Learning; 2010. Self-assessment; pp. 17–19
Oriot D, Cardona J, Berthier M, Nasimi A, Boussemart T. La voie intra-osseuse : une voie d'abord vasculaire méconnue en France. Arch Pédiatr 1994;1:684-8
Oriot D, Darrieux E, Boureau-Voultoury A, Ragot S, Scépi M. Validation of a performance assessment scale for simulated intraosseous access. Sim Healthcare 2012;7:171-5
Rosen MA, Salas E, Wu TS, Silvestri S, Lazzara EH, Lyons R et al. Promoting teamwork: an event-based approach to simulation-based teamwork training for emergency medicine residents. Acad Emerg Med 2008;15:1190-8
Rudolph JW, Simon R, Dufresne RL, Raemer DB. There's no such thing as "non judgmental" debriefing: a theory and method for debriefing with good judgment. Simul Healthc 2006;1:49-55
Rudolph JW, Simon R, Raemer DB, Eppich WJ. Debriefing as formative assessment: closing performance gaps in medical education. Acad Emerg Med 2008;15:1010-6
Schreinicke G, Hinz A, Kratzsch J, Hüber B, Voigt G.Stress-related changes of saliva cortisol in VDU operators. Int Arch Occup Environ Health 1990;62:319-21
Sexton JB, Thomas EJ, Helmreich LR. Error, stress, and teamwork in medicine and aviation: cross sectional surveys. BMJ 2000;320:745-9
Shapiro MJ, Morey JC, Small SD, Langford V, Kaylor CJ, Jagminas L et al. Simulation based teamwork training for emergency department staff: does it improve clinical team performance when added to an existing didactic teamwork curriculum? Qual Saf Health Care 2004;13:417-21
Shapiro MJ, Gardner R, Godwin SA, Jay GD, Lindquist DG, Salisbury ML et al. Defining team performance for simulation-based training: methodology, metrics, and opportunities for emergency medicine. Acad Emerg Med 2008;15:1088-97
Sloan RP, Shapiro PA, Bagiella E, Boni SM, Paik M, Bigger JT Jr et al. Effect of mental stress throughout the day on cardiac autonomic control. Biol Psychol 1994;37:89-99
Spielberger CD. Manual for the Ste-Trait Anxiety Inventory (STAI). Consulting Psychologisyts Press, Palo Alto, CA, 1983
Task Force of the European Society of Cardiology and the North American Society of Pacing and Electrophysiology. Heart variability: standards of measurements, physiological interpretation and clinical use. Circulation 1996;93:1043-65
Vaiva G, Jehel L, Cottencin O, Ducrocq F, Duchet C, Omnes C et al. Prevalence of trauma-related disorders in the French WHO study. Encephale. 2008;34:577-83
Wagner J, Cik M, Marth E, Santner BI, Gallasch E, Lackner A et al. Feasibility of testing three salivary stress biomarkers in relation to naturalistic traffic noise exposure. Int J Hyg Environ Health 2010;213:153-5
Wallenius C. Military observers' reactions and performance when facing danger. Mil Psychol2004;16:211
Weathers FW, Litz BT, Herman DS, Huska JA, Keane TM. The PTSD Checklist (PCLS): Reliability, validity, and diagnostic utility. 9th Annual Conference of the ISTSS, San Antonio, Texas, USA, 1993.
Weibel L, Gabrion I, Aussedat M, Kreutz G. Work-related stress in an emergency medical dispatch center. Ann Emerg Med 2003;41:500-6
Wetzel CM, Black SA, Hanna GB, Athanasiou T, Kneebone RL, Nestel D et al. The effects of stress and coping on surgical performance during simulations. Ann Surg 2010;251:171-6
Wilhelm FH, Grossman P, Roth WT. Assessment of heart rate variability during alterations in stress: complex demodulation vs. spectral analysis. Biomed SciInstrum 2005;41:346-51
Wolf OT. HPA axis and memory. Best Pract Res Clin Endocrinol Metab 2003;17:287-99
Yao SN, Cottraux J, Note I, De Mey-Guillard C, Mollard E, Ventureyra V. Evaluation de l'état de stress post-traumatique : validation d'une échelle, la PCL-S. Encephale 2003;29:232-8
Yee B, Naik VN, Joo HS, Savoldelli GL, Chung DY, Houston PL et al. Non-technical skills in anesthesia crisis management with repeated exposure to simulation-based education. Anesthesiology 2005;103:241-8
Zheng D, Shen L, Wu G, Jiang Y, Zhou Y, Wang W. Spectral analysis of R-R intervals in adolescent persons during mental stress. J Biomed Engin 1997;14:38-41

3. STUDY OBJECTIVES

3.1. Primary objective

To seek evidence for the existence of stress, as it will be assessed by three processes throughout the various scenarios:
- Biological stress (Salivary Cortisol [SC]);
- Electrophysiological stress (Holter 24h, timely measures);
- Psychological stress.

3.2. Secondary objectives

To evaluate performance, as it will be assessed by three processes:
- Team performance (actions, algorithm, and treatment);
- IO access performance;
- Teamwork and leadership.

To evaluate the effect of repeated simulation sessions on performance and stress:
- Performance will be evaluated using the same assessment tools;
- Stress will be investigated by studying the variations of given stress markers and coping responses.

4. STUDY DESIGN

4.1. Primary outcome measures

Our primary outcome measure will consist of revealing and evaluating evidence for the existence of stress during the simulation. Such stress will be estimated by 3 different methods:

Biological stress:

SC will be measured by an Enzyme-Linked Immunosorbent Assay (ELISA) kit (IBL international®, Hamburg, Germany). SC is commonly used to assess stress (Wagner 2010, Beko 2010, Dovio 2010) and this method has been used in simulation (Bong 2010). It will be assessed one day prior to the simulation, just before and right after the simulation session, as well as following debriefing.

Electrophysiological stress:

Holter parameters (Wilhelm 2005) (HR analysis, temporal and spectral analysis with PNN50 as well as LF/HF ratio) will be obtained using the software Synscope* (Sorin Group) throughout an entire day (24h recording) - during the resting phase, i.e., sleep, during selective periods at the time of SC sampling, as well as during the phases of simulation, debriefing, and pauses following simulation (Task Force 1996). Timely measures of HR and BP will be associated with this analysis.

Psychological stress:

will be assessed using 4 self-assessments:

- Self-assessment using STAI - *State-Trait Anxiety Inventory* (Spielberger 1983) just before and right after the simulation session, as well as following debriefing.

- Using **SOM self-rating scale** (Olpin 2010). For each self-assessment, participants will be placed in an isolated room so as not to be disturbed.

- Early post-trauma stress disorder will be determined on the 7th day following simulation, using IES-R - *Impact of Event Scale-Revised* (Brunet 2003), which will be emailed at that time to each participant.

- Late post-trauma stress disorder will be determined one month following simulation, using with PCLS - *Post-traumatic Check-List Scale* (Weathers 1993), which will be emailed at that time to each participant.

4.2. Secondary outcome measures

4.2.1.Evaluation of performance

Team performance of a simulated infant shock management will be assessed by three ways:

- ***Global team clinical performance*** will be evaluated using performance assessment scale ("TAPAS"), designed by the Simulation Laboratory and in validating process (Oriot 2013, unpublished data).
- ***IO access procedure process and success*** of the insertion technique: evaluated by
  - An assessment scale - IOPAS - *Intraosseous Performance Assessment Scale* (Oriot 2012),
  - Time to decision,
  - And duration of procedure (Oriot 2012) (cf. Appendix).
- ***Teamwork performance*** will be evaluated using the following validated scales:
  - concerning **Leadership assessment:** BAT – *Behavioural Assessment Tool* (Anderson 2010) (cf. Appendix)
  - concerning **Global Teamwork assessment:** CTS - *Clinical Teamwork Scale*, CTS (Guise 2008) (cf. Appendix)

4.2.2. Evaluation of the effect of repeated simulation sessions on stress and performance

Effect of repeated simulation sessions on stress and performance will be assessed by the same stress parameters and team performance scores.

To study evolution of stress, we shall calculate the relative variation of different variables obtained from performance scores. Relative variation will be defined by the following calculation: ([final score] - [score T0]) / (score T0)

We shall study the effect of repeated simulation on the parameters of stress. Finally, the senior physician's stress management strategies will be deduced from previous evaluations and evaluated according to 2 different manners, inspired by the axes proposed by Wetzel (Wetzel 2010):

- Observation during simulation
- Video recording viewing by the supervisors

4.3. Description of the interventions

4.3.1. Initial training of the senior physicians

All the senior physicians will have been administered, in advance in this study, identical training as to the complete procedure of pediatric IO access (theoretical and practical course using task-trainer). Theoretical and practical evaluations using an assessment scale established by the Simulation Laboratory (Oriot on 2012) for the University Course of Pediatric Emergency Procedures (Simulation Laboratory, Faculty of Medicine of Poitiers, Prof. Oriot; (Diplôme Universitaire des Gestes d'Urgence en Pédiatrie (Laboratoire de Simulation, Faculté de Médecine de Poitiers, Pr Oriot).

4.3.2. Mannequin and scenarios

We shall use a high-fidelity mannequin, SimNewB* (Laerdal*), available in the Simulation Laboratory of Poitiers, along with our bank of scenarios simulating shock in an infant (every scenario will be drawn by lots among the cases remaining). These scenarios will oblige teams to manage a simulated infant shock, within a highest level of priority, requiring insertion of an IO access. We shall use nine scenarios of infant shock, drawn by lots, among 18 available: Hypovolemic shock (3rd digestive sector, acute dehydration due to gastroenteritis, extensive burns, diabetic ketoacidosis, diabetes insipidus, post-traumatic cerebral salt wasting, adrenal failure due to congenital adrenal hyperplasia), hemorrhagic shock (subgaleal [subaponeurotic] hematoma, fulminating hemoptysis due to pulmonary artery hypertension, severe trauma with retro-peritoneal hemorrhage, severe trauma with peritoneal and femoral hemorrhage), medication or food-induced anaphylactic shock, cardiogenic shock (viral myocardiopathy, supra-ventricular tachycardia), septic shock (purpura fulminans), shock due to hemolytic anemia (malaria with acute hemolysis), and cardio-respiratory arrest complicating a decompensated state of shock due to a tamponnade.

4.3.3. Stress

Stress will be that which is connected to the very scenarios (hypoxia, haemodynamic decompensation, onset of convulsions), but also to an environment inducing a stressful atmosphere (monitor beeps and alarms), including the untimely entry of simulating parents into the emergency room, if this latter case has been foreseen in the scenario.

4.3.4. Supervisors and evaluation

Two independent supervisors at a time will evaluate each simulation session of the Sim-Stress research protocol. These supervisors, who are members of the University Hospital of Poitiers (CHU de Poitiers) Simulation Laboratory, will be chosen in random manner. They each have different specialities, and are all simulation trainers, qualified in performing debriefing sessions.

This team of supervisors will consist of Prof. Denis Oriot (pediatric intensivist), Dr Amélie Boureau (pediatric emergency physician), Dr Franck Petitpas (surgical intensivist), Dr Youcef Guechi (adult emergency physician), and 4 emergency or pediatric fellows. They will analyze global performance with the respect to algorithm, IO access performance and global teamwork - leadership and communication. The mean between the two supervisors' scores will be used as the reference value. Interobserver reproducibility will be calculated according to the scale used.

4.3.5. Video

All the simulation sessions will be videotaped, so as to be viewed for analysis of actions and interventions performed during the simulation scenario (evaluation of the global competence and strategies used to manage the induced stress). Furthermore, videos will also further be viewed for analysis by two other independent observers, who will not know the team members. The mean between the two supervisor scores will be used as the reference value. Interobserver reproducibility of the teamwork scale (leadership and communication) will be calculated. Stress

management strategies implemented by the senior physician will also re-evaluated. A comparison will be performed with the supervisors observing the live simulation sessions.

4.3.6. Debriefing

All the simulation sessions will be followed by a debriefing session, according to Rudolph's method of *"good judgment"* (Rudolph 2006, Rudolph 2008), and led by supervisors previously trained in this technique (University course Training at the University of Paris Descartes).

4.3.7. Simulation session repetition

The frequency of repeated simulation sessions will be one of the varying parameters, and will define distribution into one of two randomized groups, necessary for providing answers to secondary objectives. Repetition will take place either every 6 weeks i.e. 9 / year (group 1), or every 6 months *i.e.* 3 / year (group 2).

4.4. Study schedule

4.4.1. Time schedule of measurements

Time schedule of measurements

**Variables**		**day prior**	**Before Sim**	**Sim**	**Post Sim**	**Debrief**	**Post debrief**	**H+2**	**1 week**	**1 month**
PERFORMANCE	Global Performance			**X**						
	IO access			**X**						
	Leadership (BAT)			**X**						
	Teamwork (CTS)			**X**						
SRESS PARAMETERS	Salivary cortisol	**X**	**X**		**X**		**X**			
	Holter parameters	**X**	**X**	**X**	**X**	**X**	**X**	**X**		
	BP HR	**X**	**X**		**X**		**X**			
	SOM		**X**	**X**						
	STAI scale	**X**	**X**		**X**		**X**			
	EIS-R scale								**X**	
	PCLS scale									**X**

This plan will be repeated on each and every simulation session, that either every 6 weeks (as concerns the teams of group 1) or every 6 months (as concerns the teams of group 2).

4.4.2. Quantification of sessions and various evaluations

4.4.2.1. Number of participants included in the study

12 teams of 4 persons = **48 participants**

4.4.2.2. Number of team simulation sessions:

Participants of group 1: 6 teams x 9 sessions = 54 simulation sessions
Participants of group 2: 6 teams x 3 sessions = 18 simulation sessions
TOTAL = 54 + 18 = 72 team stimulation sessions

4.4.2.3. Number of individual simulation sessions :

Group 1: 6 teams x 4 members per team x 9 sessions = 216
Group 2: 6 teams x 4 members per team x 3 sessions = 72
TOTAL = 216 + 72 = 288 individual simulation sessions

4.4.2.4. Number of Holter recordings

Group 1: 9 Holter recordings by participant
Group 2: 3 Holter recordings by participant
Total: 1 Holter recording per session and per participant, that is, **288 Holter recordings**

4.4.2.5. Number of Heart Rate and Blood Pressure measurements

Measurements will be obtained the day before the session, then just before and just after simulation, as well as after debriefing that is 4 times per simulation session.
Group 1: 36 measurements per participant
Group 2: 12 measurements per participant
Total: 288 x 4 = 1152 blood pressure and heart rate measurements

4.4.2.6. Number of salivary cortisol concentration measurements

Salivary cortisol concentrations will be measured the day before the session, then just before and just after simulation, as well as after debriefing that is 4 times per simulation session.
Group 1: 36 cortisol concentration measurements per participant
Group 2: 12 cortisol concentration measurements per participant
Total: 288 x 4 = 1152 cortisol concentration measurements

4.5. Description of measures taken to reduce or avoid bias

4.5.1. Drawing lots

4.5.1.1. Drawing lots will constitute representative samples

Lists of persons corresponding to each of the 4 professional statuses previously defined, will be established, and will serve as the basis of random drawing lots, for allocation into each of the categories, with samples representative of each professional status, thus avoiding any selection bias. The sample size (24 persons per group) will be the double of that required by the protocol, so as take into account possible participation refusals. Those selected by the drawing lots will be contacted in the order of appearance on the drawing lots list, until 12 consenting participants per group are obtained.

Teams consisting 4 participants, one from each representative status, will be established by following the order of appearance on drawing lots list. As an example, Team 1 will consist of the 1st person on the drawing lot list who will have given consent, and who will abide by all the inclusion / exclusion criteria pertaining to each of the 4 professional statuses. It is thus that 12 teams will be constituted.

4.5.1.2. Drawing lots for random distribution of each team into one of the 2 of simulation session programs

Once the teams have been constituted, drawing lots will be performed according to a list that will been previously established by the study's methodologist, so as to distribute the 12 teams in 2 groups of 6 teams :

Group 1 will be constituted by 6 teams of 4 persons each, and each of these teams will undergo simulation sessions once every ***6 weeks***, for a total of 9 sessions in 12 months.

Group 2 will be constituted by 6 teams of 4 persons each, and each of these teams will undergo simulation sessions once every ***6 months***, for a total of 3 sessions in 12 months.

4.5.2. Homogeneity of the study's population

We have established relatively narrow inclusion criteria, so as to homogenize participants' training and/or professional experience for each of the four statuses.

4.5.3. Methods of assessment using objective evaluation scales

All the assessments using objective evaluation will be made by two observers, independent of each other, and in single manner, as far as their affectation to both groups and management of scenarios is concerned. Calculation of intra-class correlation coefficients will be performed for each of the scales used evaluation, even if such scales may have already been validated, so as to measure inter-observer reproducibility.

4.6. Study duration

Total duration of the study is estimated at **24 months**

Total duration of participation in the study for each participant: **12 months**

Starting from very first inclusion, the sponsor must immediately, and without any delay, inform the proper authorities and France's Protection Committee of Human Subjects (CPP - "Comité de Protection des Personnes) as to the effective date of study onset (effective date of study onset = date of signature of consent made by the first participant included in the study).

The date of study end will be transmitted by the sponsor to the France's National Agency of Medication and Health Product Security ("ANSM - Agence Nationale de Sécurité du Médicament et des produits de santé") and to the CPP within a period of 90 days following that date.

The date of study end corresponds to the participation end term of the last participant included in the study, or if need be, **to the end term as defined in the protocol.**

4.7. Description of rules concerning either definitive or temporary withdrawal

4.7.1. Participant withdrawal from this study

Participants will be allowed to withdraw their assent and ask leave the study at any moment, and for whatever reason. In case of a premature exit, the investigator must inform as to the reasons in as complete a way as possible.

The investigator will be able to interrupt, either temporarily or definitively, the participation of a participant in the study, for any reason that may be considered as being in the best of interest to the participant, especially in the case of serious adverse effects.

4.7.2. Partial or complete study interruption

This study may eventually be interrupted prematurely, in the case of unexpected and severe adverse events, that would merit an in depth review as to the strategy's profile. Similarly, unforeseen events that would not allow the study objectives to be credibly attained can incite the sponsor to interrupt the study prematurely.

The University Hospital of Poitiers (CHU de Poitiers) reserves the right to interrupt this study, at any time, should the objectives of inclusion not be attained. In case of a premature interruption of this study, this information will be transmitted by the sponsor to France's Protection Committee of Human Subjects (CPP - "Comité de Protection des Personnes) within the next 15 days.

4.8. Identification of all data to be collected directly in observation manuals and to be considered as data sources

A CRF paper will be provided to the investigators, in which clinical data will be directly noted by the included participants.

Data collected in the CRF will so be able to be considered as data sources. The investigator should date and sign this copy at the end of data collection.

5. INCLUSION AND EXCLUSION CRITERIA

5.1. Inclusion criteria

- All participants in the trial study will be volunteers;
- Age ≥ 18;
- All participants will be legally free and not subject to any custody, guardianship, or tutelage;
- Participants should all adhere to France's Social Security Health regime;

- Free, prior, clearly informed and written consent
- Signed agreement allowing for video-recordings that will be striclty and exclusiveley used for evaluation purposes during this study;
- Four-member teams:
  1/- Emergency physician of the Poitou-Charentes region and its surroundings, having professional experience of less than seven years and having obtained a complementary University Course in Pediatric Emergency Procedures (Diplôme Universitaire des Gestes d'Urgence en Pédiatrie) during the last 3 years, which abides by the latest recommendations of advanced paediatric resuscitation, established in 2010 by the AHA - *AMERICAN HEART ASSOCIATION* (Kleinman, 2010) and the ERC - *EUROPEAN RESUSCITATION COUNCIL* (Barient,2010);
  2/ Medical Resident ("interne") currently registered in a Complementary Specialized Diploma of Emergency Medicine ("DESC - *Diplôme d'Études Spécialisées Complémentaires* - de Médecine d'Urgence") at the University of Poitiers, and having received training in pediatric emergency procedures.
  3/ Registered Nurse of the Emergency Medical Service and Emergency Medical and Resuscitation Mobile Services ("SAMU - *Service d'Aide Médicale d'Ugence*" SMUR - " *Service Mobile d'Urgence et de Réanimation*") of the University Hospital of Poitiers (CHU de Poitiers), having a professional experience of less than seven years, and having obtained a EPILS - *European Pediatric Immediate Life Support* - diploma during the last two years;
  4/-Ambulance driver of the of the Emergency Medical Service and Emergency Medical and Resuscitation Mobile Services ("SAMU - *Service d'Aide Médicale d'Ugence*" SMUR - " *Service Mobile d'Urgence et de Réanimation*") of the University Hospital of Poitiers (CHU de Poitiers), having a professional experience of less than seven years.

5.2. Non-inclusion Criteria

- Age < 18 years
- Current participation in another clinical research study, with an exclusion period of at least month between the two research protocols;
- Participants not benefiting from a Social Security Health Regime ("Sécurité Sociale");
- Pregnant or breast-feeding women, women at an age to procreate and not using an effective contraception method (either hormonal / mechanical [*per os*, injectable, transcutaneous, implantable, intra-uterine device] or surgical [tubal ligation, hysterectomy, total ovariectomy];
- Medical and/or psychiatric antecedents that could influence the state of stress.
- Cardiac or convulsive neurological antecedents.
- Pacemaker or automatic internal cardioverter-defibrillator bearers;
- Participants using cardiotropic agents and/or beta-2 mimetic bronchodilator medication (possible modification of cardiac electrophysiological parameters).

5.3. Per-Study Exclusion Criteria (Subject Withdrawals or Discontinuation)

- Disregard of evaluation dates and schedules that could thus either compromise the simulation sessions or influence the parameters being evaluated.
- Exclusion of the entire team in case of withdrawal of one of its members.

5.4. Recruitment modalities

According to the participants' status, the mode of recruitment will differ as follows:

- 12 senior emergency physicians of the Poitou-Charentes region and its surroundings, having professional experience of less than seven years and having obtained a complementary University Course in Pediatric Emergency Procedures (Diplôme Universitaire des Gestes d'Urgence en Pédiatrie) during the last 3 years, which abides by the latest recommendations of advanced paediatric resuscitation, established in 2010 by the AHA - AMERICAN HEART ASSOCIATION (Kleinman, 2010) and the ERC - EUROPEAN RESUSCITATION COUNCIL (Barient,2010);
- 12 Medical Residents ("internes") currently registered in a Complementary Specialized Diploma of Emergency Medicine ("DESC - Diplôme d'Études Spécialisées Complémentaires - de Médecine d'Urgence") at the University of Poitiers, and having received training in pediatric emergency procedures.
- 12 Registered Nurses of the Emergency Medical Service and Emergency Medical and Resuscitation Mobile Services ("SAMU - Service d'Aide Médicale d'Ugence" SMUR - " Service Mobile d'Urgence et de Réanimation") of

the University Hospital of Poitiers (CHU de Poitiers), having a professional experience of less than seven years, and having obtained a EPILS - European Pediatric Immediate Life Support - diploma during the last two years;

- 12 Ambulance drivers of the of the Emergency Medical Service and Emergency Medical and Resuscitation Mobile Services ("SAMU - Service d'Aide Médicale d'Ugence" SMUR - " Service Mobile d'Urgence et de Réanimation") of the University Hospital of Poitiers (CHU de Poitiers), having a professional experience of less than seven years.

For each of these statuses, an exhaustive list will be established serving as a basis for random sampling. The drawn lots of participants will be contacted by e-mail, to presentation them the study and to invite them to participate. Definitive, signed consent will be obtained the day before the first simulation session, in the presence of the coordinating investigator of this study.

The modalities of the drawing lots have been described in paragraph 4.5 .1

5.5. Procedure for exclusion from the study

5.5.1. Participant study exclusion criteria and modalities

5.5.1.1. Subjects having recalled their assent and having asked withdraw from the study, will be excluded.

The investigator will be able to interrupt the participation of a study participant for any reason that would be to the best of the subject's interests, and, in particular, in the case of serious adverse effects.

A participant will be excluded from the study in case of disregard of evaluation dates and schedules, that could compromise the simulation sessions or influence studied parameters.

The exclusion of any one member will result in the exclusion of the member's entire team, and will necessitate the integral inclusion of a new team.

5.5.2. Data collection modalities and schedule of withdrawn participants

Data involving participants having withdrawn their consent will be analyzed only if those participants have responded favourably to this request.

5.5.3. Follow-up modalities of withdrawn participants

A participant's exclusion from the study will bear no consequence on ensuing professional relations regarding this person. In case of any adverse effect, be it serious or not, a precise follow-up will be put in place, in accordance with the gravity and severity of the adverse effect in cause.

6. ANALYSIS

Analysis will concern a number of study questions:

6.1. Evaluation of the effect of stress on performance

At first, we shall estimate the effect of ***stress*** on team ***performance***, using three parts:
- By obtaining evidence of the existence of a biological, electrophysiological and psychological stress, during the progress of various scenarios;
- By estimating the impact of stress on global team performance (determined by degree of respect for the appropriate algorithm);
- By estimating the impact of stress on insertion of an IO access: insertion technique (evaluation scale), decision-making timing and duration;
- By estimating the impact of stress on teamwork: evaluation of leadership (evaluation scale) and communication (evaluation scale).

6.2. Evaluation of the effect of repeated simulation sessions

Next, we shall estimate the effect of ***repeated simulation sessions*** on a team's ***performance evolution*** in terms of safety preservation as concerns an infant in a state of shock, using three parts:

- By estimating the impact of repeated simulation sessions on global team performance (determined by degree of respect for the appropriate algorithm);

- By estimating the impact of repeated simulation sessions on insertion of an IO access: insertion technique (evaluation scale), decision-making timing and duration;

- By estimating the impact of repeated simulation sessions on teamwork: evaluation of leadership (evaluation scale) and communication (evaluation scale).

Finally, we shall study the effect of the *repeated simulation sessions* on stress by evaluating various stress markers (biological, electrophysiological and psychological), as well as its impact on stress management strategies. We shall so be able to determine if *simulation session repetition*, expected to improve performance, will be accompanied by high stress levels following implementation of stress management strategies, or if it will be accompanied by a decrease in stress levels. Evaluation of the effect of the *repeated simulation session* will be evaluated by comparison of the 2 groups, i.e. the teams that will participate 9 vs. 3 simulation sessions, as described in paragraph 9

7. PERFORMANCE AND STRESS EVALUATIONS

7.1. Description of the parameters involved in the evaluation of performance

Performance will be evaluated using ***global team performance***, ***IO access insertion*** and ***teamwork*** scales, described in paragraph 4.1

Furthermore, we shall use a double evaluation (direct observation during simulation and indirect observation through viewing of the corresponding video recordings) to analyze the strategies of stress management of the implemented by each senior physician during the simulation sessions.

7.2. Description of the parameters involved in the evaluation of stress

Parameters involved in the *evaluation of stress* will be estimated for every participant, and are described in paragraph 4.2.1

7.3. Methods and schedule for collection, quantification and analysis of performance and stress evaluation parameters

7.3.1. Performance quantification

Performance quantification will be based upon objective evaluations obtained using a number of evaluation scales (global team performance, IO access, leadership, and teamwork), as conducted by two independent supervisors, direct observers of each simulation session.

Subsequent video recording visualisation by the same two, direct observers, will allow for evaluation of stress management strategies implemented by the team leader.

7.3.2. Quantification of stress

Quantification of stress will make of use various tools and particular attention:

- Salivary samples for cortisol concentration measurements will be taken one day before the simulation session (at approximately 18H00), the day of the simulation session, just before and right after the simulation session - and preceding as well as following - the debriefing session. Tubes will be preserved in a refrigerator allocated for that purpose in the Simulation Laboratory, and then be sent to the Biochemistry laboratory of the University Hospital of Poitiers (CHU de Poitiers) for analysis (Dr Christine Millet and Dr Aiham Ghazali).

- A Holter device will be placed on each of the male participants by Dr A. Ghazali, and on each of the female participants by Emmanuelle Fournier, the day before the simulation session (at approximately 18H00). The Holter device will be worn for a total duration of 24 hours. Since the simulation sessions will take place between 14H00 and 15H00 in the Simulation Laboratory, the Holter device will remain in place during 3 hours following the simulation session's end. The recording cassettes will be removed, collected and read once a week in the cardiology department of the University Hospital of Poitiers (CHU de Poitiers) (Dr Philippe Sosner and Dr Aiham Ghazali).

- A non-invasive measurement of blood pressure will be made the day before the simulation session (at approximately 18H00), the day of the simulation session, just before and right after the simulation session - and preceding, as well as following - the debriefing session. Every measure of blood pressure will be associated to a timely measure of the participant's heart rate.

- Quantification of psychological stress will make use of three questionnaires and SOM score after simulation:
    - SOM: is a score between 0 and 10 obtained by self-assessment after simulation sessions

- STAI is a self-assessment questionnaire, used for estimating both basal and induced, acute stress. Participants will fill out the questionnaire in an isolated room, the day before the simulation session (at approximately 18H00), the day of the simulation session, just before and right after the simulation session - and preceding, as well as following - the debriefing session.
- EIS-R is a self-assessment questionnaire, used for estimating early post-traumatic stress, 7 days following a trauma (in our case, "trauma" corresponds to the simulation session). This questionnaire will be sent out to the participants by e-mail at this time. Answers will be obtained by email return, and collected by a clinical research assistant.
- PCLS is a self-assessment questionnaire, used for estimating late post-traumatic stress, 28 days following a trauma (in our case, "trauma" corresponds to the simulation session). This questionnaire will be sent out to the participants by e-mail at this time. Answers will be obtained by email return, and collected by a clinical research assistant.

8. ASSESSMENT OF SAFETY

8.1. Evaluation parameters concerning safety

Clinical tolerance during the research study will be objectivised by analysis of all adverse and/or intercurrent events that may arising during the research study, and which should systematically be reported within the observation notebook.

8.2. Methods and schedule for collection, quantification and analysis of safety evaluation parameters

Adverse events will be evaluated at each and every session during the entire study phase.

It will be asked of every investigator to note: the severity of the event, the type of event, its intensity, its relation with the simulation session (improbable, likely, not assessable), the date of onset, its duration, and treatment initiated.

8.3. Procedures set up for recording and reporting adverse events

8.3.1. Characteristics of an adverse event

Adverse event (AE): any untoward or unfavourable medical occurrence in a human subject participating in biomedical research, whether that occurrence is connected or not to either the study or the experimental procedures carried out during the study.

A **severe adverse event (SAE)** meets one or more of the following criteria:

- Results in death;
- Is life-threatening (places the subject at immediate risk of death, at the time the event occurs, and this, independently of all consequences any corrective or palliative therapy might provide);
- Results in important or durable incapacity or handicap;
- Results in inpatient hospitalization or prolongation of existing hospitalization;
- Results in a persistent or significant disability or incapacity;
- Any alarming event (undesired clinical event or alarming laboratory result or that may be considered as such by the investigator).

Side Effect (SE): any harmful and undesirable reaction related to the strategy

Serious Side Effect (SSE): any serious and undesirable reaction attributable to the strategy.

Unexpected Side Effect (USE): any undesirable effect of which nature, severity, intensity or evolution is not concordant with information appearing in the investigator's brochure.

Imputability: relationship between an Adverse Event (AE) and the study's strategy. Any AE found to be related to the study's strategy will be thereafter considered as a **Side Effect (SE)**. Factors to be taken into account for the determination of imputability are:

- Timeline of events;
- The Adverse Event (AE) abates when the intervention is discontinued once the strategy has ended, and/or reappears upon a re-challenge with the intervention;
- Notion of a similar event having occurred during the strategy's implementation
- Existence of some other aetiology (causation).

Intensity: the severity of a given Adverse Event (AE) will be estimated by the investigator, according to the following classification scale:

- **Mild, level 1:** an Adverse Event that is generally transitory, with no impact on activities of daily living (ADL);
- **Moderate, level 2:** an Adverse Event annoying enough so as to incur moderate impact on ADL;

- Major, level 3: an Adverse Event that either incurs considerable impact on the participant's activities of daily living (ADL), or causes some form of invalidity, or constitutes a threat for the life of the participant.

Remark: Adverse Event *Intensity* should not be confused adverse event *Severity*, which serves as a guide to define the various reporting obligations and procedures.

8.3.2.Role of the investigator

8.3.2.1. Reporting of severe adverse events (SAEs)

8.3.2.1.1. Information to be passed on to the sponsor

Every SAE will be described on the form provided for that purpose ("Initial Report of a Severe adverse event" - « Déclaration Initiale d'Evénement Indésirable Grave » or "Follow-up Report of a Severe adverse event" - « Déclaration de Suivi d'Evènement Indésirable Grave ») all in trying to as most exhaustive as possible.

Information to be reported includes the following:
- Participant's Identification (number, code, date of birth, date of inclusion, sex, weight, height),
- Severity of the adverse event,
- Date (and time) of onset and end of the adverse event,
- Clear and detailed description of the adverse event (diagnosis, symptoms, intensity, chronology, actions - Initiated and various results obtained),
- Evolution of the adverse event,
- Participant's current diseases or relevant antecedents
- Treatments received by the participant,
- Link of causality of the adverse event with the simulation session, the eventual treatments been taken, the study itself, or with any other criteria.

Along with the adverse event report, the investigator should also include the following, whenever possible:
- A copy of the hospitalization report of (if the participant were to be hospitalized);
- A copy of the autopsy report, in the case of death;
- A copy of all the results of all complementary exams performed, including relevant negative results, by appending, as well, all the normal value ranges as provided by laboratory;
- Any other document that the investigator might consider as either useful or relevant.

These documents will be processed by making the participant's identity anonymous, using the participant's identification number alone.

8.3.2.1.2. Procedures for reporting a severe adverse event to the sponsor

All SAEs, whatever their relation of causality with the present study (with the exception of those are listed in the protocol as not requiring any immediate reporting), must be reported:
- By fax: +335 49 44 30 58.

One can join the person responsible for the University Hospital of Poitiers Vigilance Center (Centre de Vigilance, Direction de la Recherche, CHU de Poitiers), Dr. Sophie DURANTON:

- By call: +335 49 44 03 50
- By fax: +335 49 44 30 58
- By email sophie.duranton@chu-poitiers.fr

8.3.2.1.3. Delay period for reporting to the sponsor

All SAEs must necessarily be reported to the study's sponsor within **24 hours** of their onset (or as soon as the investigator physician has been notified).

The initial report may be followed by further relevant information either within **8 days** in the case of a fatal event or life-threatening prognosis, or within **15 days** in all other cases.

8.3.2.1.4. Time period for reporting to the sponsor

The investigator has the responsibility to note and report all SAEs that may arise during the entire course of the study:
- Starting from the date of signature of the consent,
- Pursuing throughout the entire duration of participant follow-up foreseen by the protocol,
- And extending up to 4 weeks after participant follow-up termination.

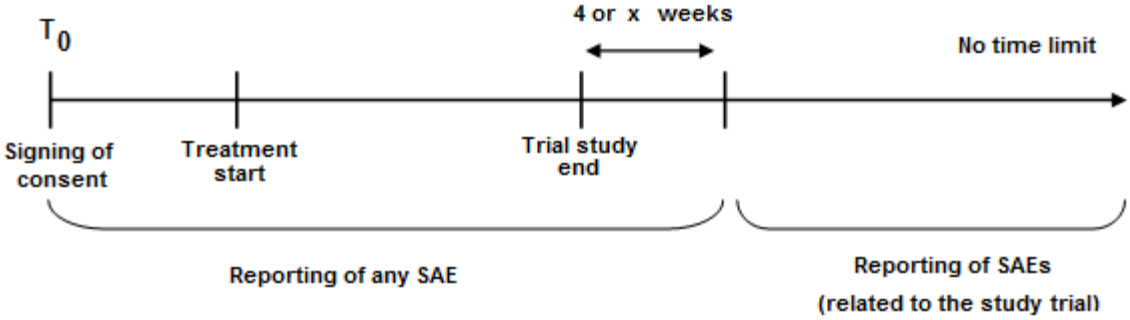

8.3.2.1.5. Specificities of the protocol

Severe adverse event not subjected to immediate reporting:

Certain circumstances requiring hospitalization may not actually arise from severity criterion "hospitalization" and, as such, should not be declared as severe adverse events

- Admittance for social or administrative reasons
- Hospitalization for routine treatment or surveillance of the studied pathology, and not associated to any deterioration of the participant's initial health state
- Hospitalization for scheduled medical or surgical treatment before the trial's start.

8.3.2.2. Reporting of non-severe adverse events

All the other Adverse events will be reported on the form "Adverse event" (« événement indésirable ») included in the observation notebook, while noting the following: date of onset, description, intensity, duration, mode of resolution, aetiology, imputability and decisions taken.

8.3.3.Role of the sponsor

8.3.3.1. Analyze severe adverse events(SAEs)

The sponsor shout evaluate:

- **The causality of each SAE:** all adverse events, for which the investigator or the sponsor estimates that there exists reasonable argument in favour of a relation of causality with the study's strategy, will be considered as putative undesirable effects. In case of evaluation divergence between the sponsor and the investigator, the two opinions will be mentioned in a report that will be sent to the proper authorities, should such a report be necessary,
- **The expected/unexpected character of each SAE,** by using the most current reference document (brochure for the investigator)

8.3.3.2. Scoring imputability

According to ICH recommendations on management of clinical trial adverse events - ICH E2B (R3), version of May 12, 2005 - an evaluation of imputability is to be performed for every SAE reported. The scoring method is as follows:

- **No relation:** the event appears in an incompatible timeframe period (delay) with regard to the study's implementation, and/or there is a sufficient information indicating that the observed reaction has no relation with the study, and/or there exists a likely alternative explanation.
- **Doubtful relation:** the event's chronology (appearance, evolution) is weakly compatible with regard to the study's implementation timeframe, and is apparently attributable to factors other than those of the study's strategy, such as the participant's clinical state, or the concomitant administration of other products.
- **Possible relation:** the event appears at a moment that is compatible with regard to the study's implementation timeframe, and could not reasonably be attributed to some other factor, such as the participant's clinical state, or the concomitant administration of other products. The evolution after study termination should also be compatible, and no other pharmacological or pathophysiological explanation should account for the event.
- **Highly plausible relation:** the event appears at a very suggestive moment within study's implementation timeframe, and could not reasonably be attributed to some other factor, such as the participant's clinical state, or the concomitant administration of other products. The evolution after study termination should also be compatible, and some pharmacological or pathophysiological explanation should account for the event.

Adverse events presenting a doubtful, possible, likely or highly likely relation with the study's strategy, should be considered as being connected to the trial. Should they be unexpected, they are to be qualified as Unexpected Adverse Events, and should be reported by the sponsor (cf. Following paragraph).

8.3.3.3. Reporting Unexpected Serious Side Effects

The sponsor will declare all serious, unexpected side effects to the France's National Agency of Medication and Health Product Security ("ANSM - Agence Nationale de Sécurité du Médicament et des produits de santé"), to France's Protection Committee of Human Subjects (CPP - "Comité de Protection des Personne") and to the investigators. A statutory report will be made within a maximum delay of **7** calendar **days** for all unexpected serious side effects. Any additional, relevant information must tracked, and be transmitted within a further maximum delay of **7** calendar **days**.

8.3.3.4. Annual transmission of safety reports

One year after the study's onset, the sponsor will write out a safety report that will include:

- A list of all serious side effects, expected or unexpected, susceptive to being connected to the study's strategy,
- A concise and critical analysis as to the safety of all participants who will lending themselves to this study.

This report can eventually be handed to the coordinating investigator for approval. It will be sent to both, the France's National Agency of Medication and Health Product Security ("ANSM - Agence Nationale de Sécurité du Médicament et des produits de santé") and France's Protection Committee of Human Subjects (CPP - "Comité de Protection des Personne"), within a delay of **60 days,** starting one year following the study's implementation onset.

8.3.3.5. Reporting other safety data

This report will include any data concerning safety, or any new fact that could significantly modify the trial study's benefit/risk evaluation or that could lead to envisage modifications concerning study implementation. For example:

A) Any clinically significant increase in the frequency of appearance of an expected serious side effect;

B) Any suspicion of unexpected, serious side effect, that will have manifested itself in participants having already finished with the trial, and that will have been reported by the investigator or the sponsor, accompanied by any eventual follow-up reports;

C) Every new fact concerning the progress of the clinical trial or the development of the study strategy, when this new fact is susceptible to pose a potential threat to the health and safety of the participants.

D) The recommendations of the independent surveillance committee, if need be, if they are deemed to be relevant for the safety of the participants.

E) Any unexpected serious side effect reported to the sponsor by another sponsor, of a clinical led in anothe country, and concerning the same not pharmacological intervention.

The sponsor must make a report to the France's National Agency of Medication and Health Product Security ("ANSM - Agence Nationale de Sécurité du Médicament et des produits de santé") and France's Protection Committee of Human Subjects (CPP - "Comité de Protection des Personne") as soon as possible, and at the latest within a delay of 7 calendar from the moment the sponsor has been informed. Any additional, relevant information must tracked, and be transmitted within a further maximum delay of **7** calendar **days**.

8.3.4. *In utero* exposure

Pregnancy is not expected during the study (exclusion criteria). Any pregnancy should be declared at once to the sponsor.

The investigator should then inform the person responsible for the sponsor's Clinical Trial Pharmacovigilance Center (Pharmacovigilance des Essais Cliniques), and send a Serious Adverse Effect report by fax, that will include the predicted date of birth, as well as coordinates of the obstetrician and maternity ward that will be involved with delivery, should the pregnancy be continued.

The investigator should pursue patient follow-up until pregnancy comes to term, or is interrupted, and report the overall evolution to the sponsor.

Should the pregnancy's evolution be accounted for by the framework defining of severe adverse effects (miscarriage with hospitalization, foetal death, innate abnormality...) the investigator must then follow the procedure for reporting Severe Adverse Effects.

8.3.5. Monitoring Committee

8.4. Participant follow-up procedures and duration following an adverse event

Every adverse event will be followed until its complete resolution (stabilization at a level considered as acceptable by the investigator, or return to the initial state), even in the case the participant went out of the research.

9. STATISTICS

Software utilised: software SAS version 9.0.

9.1. Description of statistical methods intended for use, including intermediate analyses agenda

9.1.1. Descriptive analysis

Quantitative variables (various scores, cortisol concentration values, participant heart rates, RR' intervals,...) will be described by the mean and standard deviation, or the median and interquartile intervals.

Qualitative variables (age, sex, professional status, years of professional experience) will be summarized by the actual samples size and corresponding percentages for each of the modalities.

9.1.2. Evaluation of the effect of stress on performance

The relationship between

- The various variables and indicators of stress (salivary cortisol concentration, Holter parameters of, scores from the various stress scales) evaluated during the 1-st session (basal stress) and during the following sessions,
- And performance (global performance, IO insertion and teamwork evaluation scores), evaluated during the latter sessions,

will be studied by calculating a Pearson correlation coefficient (or Spearman correlation coefficient if need be). This calculation will be realized on the entire study population as well as within each of the groups 1 and 2. A search for any sort of heterogeneity based on status (senior physician, junior physician, nurse, ambulance driver) will be undertaken, as well.

9.1.3. Evaluation of performance score variations

Variations in global team, IO access, and teamwork performance scores will be estimated in terms of temporal sequence. In each of the groups 1 (9 sessions) and 2 (3 session), score modifications between the various simulation sessions will be tested by means of an ANOVA for repeated measures (or the Friedman non parametric test, if need be).

Concerning the entire study population, linear mixed effects models (for continuous responses observed over time) will eventually be used envisaged so as to take into account, in a common analysis, the entire set of data collected throughout the various sessions, and, more notably, parameters involved in the development of stress management strategies compared to stress and repeated simulations.

9.1.4. Comparison of the variation in performance scores between groups 1 and 2 = Evaluation of the effect of repeated simulation on performance

To study variation in performance (global performance, IO access and teamwork evaluation scores) between the time point of inclusion (T0) and the simulation program's endpoint, the relative variation of various scores will be calculated {([final score] - [score T0]) / (score T0)}. These relative variations will be compared between the 2 groups using a Student's t-test. An ANOVA test will be used to search for a possible effect due to participant status.

9.1.5. Comparison of stress between groups 1 and 2 = Evaluation of the effect of repeated simulation on stress

The various stress markers (biological, electrophysiological and psychological) will be compared between the 2 groups 1 and 2 by means of a Student's t test or, if necessary, a Mann-Whitney non parametric test.

9.1.6. Inter-observer reproducibility

Inter-observer reproducibility will be estimated by the calculation of the intra-class correlation coefficient for the various evaluation scales used.

9.2. Number of participants to be included in this study and statistical justification

The necessary number of participants was calculated to answer the main objective of the study: determine if there is relationship between stress and performance.

To determine if there is a relationship between stress and performance, such that the correlation coefficient will attain a value of at least 0,50, with type I error at 5 % and power at approximately 90%, using a bilateral test, the number of subjects to be included is calculated to be 48 (Proc POWER, SIEVE). Our study will include 12 teams of 4 persons each, and each team will be include representatives from four status categories (a senior physician, a junior physician (resident ["interne"]), a nurse and an ambulance driver.

To answer secondary objectives, the teams will be distributed in 2 randomised groups, 1 and 2.

It is noteworthy that in a previous study, a sample size of 12 participants each of the professional categories allowed for finding a difference of 2,1 points on an IO access evaluation scale of 20 points (Oriot 2012), knowing that the standard deviation of this score was described to be 1,02 in a previous study.

On the other hand, there is lack of sufficient information in the literature with regard to the number of subjects necessary to determine if there is a difference in the variability of various scores. The power of our study for questions corresponding to secondary objectives will thus be calculated *a posteriori* from the data actually observed.

9.3. Degree of statistical significance

A ***p*** score < 0.05 will be considered as statistically significant

9.4. Statistical criteria for study termination

No statistical criterion for study can be defined because no intermediate statistical analysis is foreseen.

9.5. Method for considering missing, unused or invalid data

All the information required by the protocol must be recorded, and an explanation must be provided for any missing, unused or invalid data.

9.6. Management of potential analysis modifications

Any deviation from the statistical analysis scheme will be clarified and justified in the final analysis report.

10. RIGHT OF ACCESS TO DATA AND DOCUMENTS SOURCE

10.1. Access to data

According to the BPC:

- The sponsor is commissioned to obtain an agreement from all the parties involved in research so as to guarantee direct access to all the localities, data sources, document sources and reports, for the purpose of quality control and audit by the sponsor.
- The investigators will provide biomedical research personnel in charge with the follow-up, quality control and audit, all documents and individual data strictly necessary for this control, according to current legal and statutory measures (articles L.1121-3 and R.5121-13 of France's Public Health Code - "*Code de la santé publique*").

10.2. Source documents

Source documents being defined as any original document or object allowing to prove the existence or exactness of data or facts recorded during study, will be kept during 15 years by the investigator.

10.3. Confidentiality concerning data

In conformity with the dispositions pertaining to the confidentiality of data that can be accessed by biomedical research personnel in charge with the follow-up, quality control and audit (article L.1121-3 of France's Public Health Code - "*Code de la santé publique*"),

in conformity with the dispositions pertaining to the confidentiality of information, notably; the nature of experimental medicines, trial studies, participants who lend themselves to experimentation and obtained results (article R. 5121-13 of France's Public Health Code - "Code de la santé publique"),

all persons having direct access to such data and information will take all the necessary precautions to insure the confidentiality of information relative to the experimental medicines, to study trials, participants who lend themselves to experimentation (notably with regards to their identity) as well as to obtained results.

These persons, similarly to the investigators themselves, are bound by professional secret (according to conditions defined in articles 226-13 and 226-14 of France's Penal code).

During the biomedical research study, or upon its termination, all data collected on the persons who lend themselves to experimentation and passed on to the sponsor by the investigators (or all other specialized speakers), will previously have been made anonymous.

Under no circumstance will the participants' names or addresses appear clearly.

Only participants' name and surname initials will be recorded, followed by a code number appropriate for the study, indicating the participants' order of inclusion.

The sponsor will make sure that every person who lends himself to research experimentation will have previously granted written consent, allowing for access to personal data, and strictly necessary for quality control of the study.

11. CONTROL AND ASSURANCE OF THE QUALITY

A clinical research assistant ("Attaché de Recherche Clinique" or "ARC") appointed by the sponsor will guarantee good trial study conduct as well as data collection and entry, in accordance with the Standard Operating Procedures ("Procédures Opératoires Standards") applied within the Poitiers University Teaching Hospital (CHU de Poitiers) and according to Good Clinical Practice guidelines as well as to current legal and statutory measures.

The principal investigator and all team members will agree to be available during Quality Control visits that will be made at regular intervals by the clinical research assistant.

The following elements will be reviewed during each such visit:

- Free, prior, clearly informed and written consent
- Abidance by the study protocol and the procedures which are defined therein
- Quality of data input: exactness, missing data, coherence of input data with "source" documents (medical files, appointment diaries, originals of laboratory results of, etc. …)
- Management of eventual products.

11.1.1. Quality assurance of the study safety, progress and data validity.

The investigators must commit to accepting quality assurance reviews, audits, and evaluation that will be realised by the sponsor, as well as inspections made by competent authorities. All data, documents and reports can be the object of audits and statutory inspections, without breach of medical confidentiality.

12. ETHICAL CONSIDERATIONS

12.1. Committee of Protection of Human subject

The trial study protocol, information form and certificate of consent, shall all be subjected for review to France's Western Region, Section III, Protection Committee of Human Subjects (CPP - "Comité de Protection des Personnes Ouest III")

Announcement of favorable endorsement by CPP will be passed on to the study's sponsor and competent authorities.

A request for authorisation will be sent by the Sponsor to France's National Agency of Medication and Health Product Security ("ANSM - Agence Nationale de Sécurité du Médicament et des produits de santé") before the beginning of the study.

12.2. Substantial modifications

In case of any substantial modification should brought to the protocol by the investigator, it must priorly be approved by the sponsor. The latter should have obtained, before its implementation, a favorable endorsement from the behalf of France's Protection Committee of Human Subjects (CPP - "Comité de Protection des Personnes), as well as authorisation from France's National Agency of Medication and Health Product Security ("ANSM - Agence Nationale de Sécurité du Médicament et des produits de santé") within the framework of their respective competence.

A new consent from the participating study subjects shall be obtained, if need be.

12.3. Information to participants and written consent form

Participants will be informed in understandable terms, in a complete and loyal manner, as to the objectives and constraints of the study, the possible incurred risks, the necessary surveillance and safety methods, their right to refuse to participate in the study, and of the possibility withdrawal at any time.

All this information appears on an information and consent form that will be handed to the participant.

The participant's free, prior, clearly informed and written consent, shall collected by the investigator, or a physician who will act as his representative, before the participant's definitive inclusion in the study.

The information and consent forms signed by two parties shall be handed back to the participant, and the investigator shall keep the original

At the end of study, a copy will be placed in a sealed, tamper-proof envelope, which will contain the entire set of consent forms, which will be archived by the sponsor.

12.4. Post-study exclusion period from other studies

The post-study exclusion period defined within the framework of this study is of 1 month, and corresponds to the time period during which the participant will not be able participate in any other clinical research following the present study's endpoint.

12.5. Management related to the study

Non applicable

12.6. Participant financial compensation

Participants will not receive any money for their participation in this study.

Learning by simulation on high-fidelity mannequin, with an entire team, costs 300€ per person per day. All participants will therefore benefit, free of charge, from this education in return for their participation in the study.

12.7. Registration in France's national register of persons lending themselves to biomedical research

From a statutory point of view, all participants will be registered in France's national register of persons lending themselves to biomedical research.

13. DATA HANDLING AND CONSERVATION OF DOCUMENTS AND DATA

13.1. Observation

A paper CRF - case report form - will be elaborated for this study.

All information required by the protocol must be recorded in the appropriate observation notebooks, and an explanation must be provided for each and every missing datum. Data should be captured as they are progressively obtained, and transcribed in these observation notebooks in a net and legible any way.

Any erroneous data found in the observation notebook will be clearly stricken, and the correct data will be copied, next to the barred information, accompanied with initials, date and, eventually, justification by the investigator or authorized person who will have made the correction.

13.2. Data capture and processing

Data capture will be realized electronically, using Office Access®, by the clinical study's engineer, who will be assisted by the investigators.

Data analysis will be realized by Dr Stéphanie RAGOT, the statistician and research methodologist.

13.3. National Commission for Data Protection and Liberties (CNIL - Commission nationale de l'informatique et des libertés*)*

This study is consistent with France's Reference methodology (MR-001) (« Méthodologie de Référence » [MR-001]), under the provisions of article 54 paragraph 5 of law n°78-17 of January 6, 1978 - article 54 alinéa 5 de la loi n°78-17 du 6 janvier 1978), which has been modified relative to digital data, databases and citizen liberties. This homologation change was approved by the decision of January 5, 2006. The Poitiers University Teaching Hospital (CHU de Poitiers), sponsor of this study, has signed a commitment of conformity to this "Reference methodology".

All reports having to be made within MR-001framework shall be handled by a of the Poitiers University Teaching Hospital's (CHU de Poitiers) LASH Committee of Data Protection and Liberties (CIL - Comité Informatique et Liberté) .

13.4. Filing

The following documents will be archived, using the study's name, in the premises of the Simulation Laboratory of Poitiers, until expiration of the practical utility period:

- Protocol and appendices, and eventual amendments,
- Original information and signed consent forms
- Personal identification data (authenticated copies of the raw data)
- Follow-up Documents of
- Statistical Analyses
- Final Report of the study

Following expiration of the practical utility period, the documents to be archived, as defined in the procedure of of the Poitiers University Teaching Hospital (CHU de Poitiers) "Classification and filing of documents related to biomedical research" (« classement et archivage des documents liés aux recherches biomédicales »), will be transferred to the Poitiers University Teaching Hospital's (CHU de Poitiers) Central Archives Service, and will be placed under the responsibility of the Sponsor for a period 15 years as to the study's endpoint, and according to institutional practices.

No movement and no destruction whatsoever shall be done without the signed consent of the Sponsor. At the end of this 15 year period, the sponsor will be consulted for destruction. In the meanwhile, all the data, documents and reports will be available for any eventual audit or inspection.

14. INSURANCE

The Sponsor will subscribe, for a period covering the entire duration of this study, an insurance policy that will guaranteeing his own civil liability as well as that of every person involved with the realization of the study. The sponsor will also subscribe an insurance policy guaranteeing complete compensation of any harmful consequences attribuable to the study, to all those persons who shall lend themselves to experimentation in this study, and their legal benefactors, except if proof can be provided that the harm in case is not attributable to the study or to the fault of any given person involved with the realization of the study, and without the possibility of opposition due to a third party's action, or due to the voluntary retreat of the person who had initially consented to participating in the study.

15. FEASIBILITY OF THE STUDY

This biomedical study was considered as feasible, from a number of perspectives:

- Simulation is a learning method that is rapidly expanding, and can be of great interest to numerous health professionals who will be able to benefit from it for free all in participating in some biomedical research study.
- The research study protocol will unite in one time point both a multi-disciplinary simulation session and a subsequent debriefing session, directed towards knowledge and know-how acquisition, which will be interesting for numerous professionals who have never had this type of learning possibility.
- The premises of the Simulation Laboratory (SiMI - "Laboratoire de Simulation") – offer a very friendly environment, during the simulation session as well as the debriefing, 3 hour "wash-out" phase that will ensue.

- Finally, we have thus far obtained great interest for this study from the behalf Poitiers University Teaching Hospital (CHU de Poitiers) personnel, which facilitate voluntary recruitment.

16. PUBLICATION POLICY

Communications, scientific reports and publications corresponding to this study will be realized under the responsibility of the principle coordinating investigator of the study and that of the scientific leader. Co-authors of the report and publications will be the clinicians involved, as to the proportion of their contribution to the study, as well as the biostatisticians-methodologists and all associated researchers.

The publication policy will follow international recommendations (N Engl J Med, 1997; 336: 309-315).

17. PROPERTY - RESULT EXPLOITATION AND VALORISATION

The Sponsor is the unique owner of the results that will be obtained in this research study. The Sponsor will be the only entity authorized to apply, in all priority and in his proper name, for a patent in France, with extensions abroad, that will cover the research study results

Following request, the Sponsor will communicate to the associated centre, the results of the research study in a final report, at the latest one year following the research study's endpoint.

18. LIST APPENDICES

Appendix 1: Form of participant information

Appendix 2: Form of participant consent

Appendix 3: Declaration of Helsinki

Appendix 4: Form of statement of a severe adverse event

Appendix 5: Intra-Osseous Performance Assessment Scale IOPAS

Appendix 6: Behavior Assessment Tool BAT

Appendix 7: Clinical Teamwork Scale CTS

Appendix 8: State-Trait Anxiety Inventory STAI

Appendix 9: Impact of Event Scale-Revised IES-R

Appendix 10: Post-traumatic Check List Scale PCLS

18.1. Appendix 1 : Information document given to each research study participant

Madam, Sir

Doctor Aïham GHAZALI offers you to participate in biomedical research study called « *SIM-STRESS :EFFECTS OF STRESS AND SIMULATION IN TEAM PERFORMANCE DURING MANAGEMENT OF AN INFANT IN A STATE OF SHOCK* », whose sponsor is the Poitiers Universty Teaching hospital (Centre Hospitalier Universitaire de Poitiers, 2 rue de la Milétrie, BP 577, 86021 Poitiers cedex)

The protocol, registered as ID-RCB: 2013-A00648-37, as well as the letter of complete, loyal information before consent, the promoter of which is the TEACHING HOSPITAL of Poitiers, has obtained favorable positive endorsement from the behalf of the of the France's third Western Region Protection Committee of Human Subjects (CPP - "Comité de Protection des Personnes Ouest III") on XX / XX / 201X, as well as permission to pursue from France's National Agency of Medication and Health Product Security ("ANSM - Agence Nationale de Sécurité du Médicament et des produits de santé") (ANSM), XX / XX / 201X.

Reflection period The document of information that has been handed to you, will allow you to decide as to whether or not you wish to participate in this study. We wish to thank you for taking the time to read the following attentively. Do not hesitate to ask members of the Simulation Laboratory research team for any explanations you might consider necessary. Your participation must be entirely voluntary. If you do not wish to take part in this study, your professional relations with the persons supervising this research project will, under not be changed under any circumstances.

Justification of the study It is known that stress can corrupt performance, but there are no facts available scientific literature regarding the benefit of repeated simulation sessions on the importance of stress and its relation with performance.

Study objectives This study concerns translational research - entirely by simulation - aiming at assessing **the influence of stress on the performance of a team**, composed of a senior physician, a resident ("interne"), a registered nurse and an ambulance driver. Furthermore, the secondary objective of this study will be to repeat simulation sessions of and to determine the influence of repetition on the performance of a team.

Conduct of the trial study Should you agree to participate in this research, you will be solicited **to participate in team simulation sessions of**, at most, once every 6 weeks (9 sessions of simulation in 12 months) or at the very least, once every 6 months (3 sessions of simulation in 12 months). Every session will last 1 hour at most, which includes a debriefing session.

The elements which will allow us to measure performance are validated scales concerning a number of urgent procedures and teamwork. Also, all simulation sessions of will be subjected to **video recording**. The subsequent use of these video pictures will be made only as part of research.

The elements that will allow **to measure stress** in a reliable way are a Holter ECG, salivary cortisol concentrations and three questionnaires. There will be no blood samples taken. A **Holter ECG** will be put on you the night before (at about 18H00) simulation session, and will be removed 3 hours following the session (total of 24 hours). A punctual measure of arterial pressure and of cardiac frequency (to assess the autonomous nervous system in conditions of stress) will be performed four times: the day before the simulation session (at about 18H00), and the day of the simulation session - just before and right after session (before the debriefing session) and after the débriefing session.

Salivary cortisol levels will be analyzed (1 ml of saliva) by four salivary samples: the day before the session of simulation (at about 18 h), the day of the simulation session of, just before and after session (before the debriefing session) and after the debriefing session. **Questionnaires on stress**, concerning basal and stress-induced anxiety, will have to be filled out the day before the session of simulation (at about 18 h), the day of the simulation session of, just before and after session (before the debriefing session) and after the debriefing session. Two other questionnaires, concerning early and late post traumatic stress, will have to be filled out by electronic mail, 7 days and then 1 month following each simulation session, respectively.

This study asks for a participation from your behalf of approximately 60 minutes per session, either 3 times or 9 times within a one year time span. Each session will begin at 14H, and will include a simulation session (at most 30 mn), that will be assessed by two independent supervisors, in the course of which you will play a role equivalent to your professional status, in a most natural possible way.

Following the simulation session, you will remain in room devoted to relaxation, within Simulation Laboratory's premises, until 17h, which corresponds to the end of explorations.

Expected benefits: The **benefits** you may gain include receiving free, assessed and innovative practical education, on medical and relational difficulties faced during complex pediatric management.

Potential risks: Risks linked to participation include the lack of comfort caused by wearing the Holter ECG during 24 hours, and being subjected to four salivary samples.

Voluntary participation Your participation in this research study must be entirely on a volunteer basis, and will be totally free of cost. You are free to accept or to refuse to participate. You are free to change the mind any time, and to withdraw your consent without having to justify yourself or your decision - this will cause no harm to your professional relations with regard to the persons supervising the research trial study. Data obtained from you during this research study will be analyzed only if you give us your consent. If new information should appearedin the course of study, that could put to question your agreement to participation, you would be immediately informed. If this study should be stopped or if your participation must be interrupted, your professional relations with the persons supervising this research trial study will not be changed under any circumstances. You are free to interrupt your participation any time.

Confidentiality of data As part of the biomedical research in which the TEACHING HOSPITAL of Poitiers offers you to participate, your personal data will be processed folowing a number of steps and procedures implemented that will their analysis with regard to the objectives you were presented with. To this end, physiological data concerning you, and data relating to your habits of life, will be transmitted to the research study's sponsor. These data will be identified by your initials followed by number code. These data, and in conditions assuring their confidentiality, might also be be transmitted to French and foreign health authorities. In accordance with the dispositions of law relating to information technology filing and individual freedoms, you have a right to access and correction. You also have a right to oppose transmission of data, already covered by professional confidentiality (medical secrecy), and likely to be used as part of this research study and be analysed. You can also have access to the integrality of study results concerning you, by referring to **doctor Aïham GHAZALI** (Emergency - Medical Ambulance Department, Centre Hospitalier Universitaire de Poitiers , University of Poitiers Teaching Hospital, 2 rue de la Milétrie, BP 577, 86021 POITIERS cedex Phone : 05 49 44 37 08. You may also consult these and all medical data either directly or through a doctor of your choice, in accordance with the dispositions of article L1111-7 of France's Public health Code.

Ethical considerations: This study will proceed in compliance with:

- the statement of Helsinki updated (October, 2008),

- the Good Clinical Practice Directive (Directive 2005/28/EC of 8 April 2005) of the European Parliament and of the Council

- the International Conference on Harmonistaion guidelines and recommendations for Good Clinical Practice (ICH-E6)

- France's Code of Federal Regulations and guidelines for Good Clinical Practice pertaining to biomedical research concerning medicines for human use (decisions of November 24, 2006);

- the France's national legislations and the regulations relative to clinical trials;

- the conformity with the European Unions' Directive regarding clinical trials on medicinal products for human use [2001/20/EC];

- Public Health law n°2004-806 of August 9th, 2004 (replacing law 88-1138 of December 20th, 1988; amendment called « Huriet-Sérusclat law, relating to the protection of those persons taking part in biomedical researche (better known as "law Huriet") - loi de Santé Publique n°2004-806 du 9 août 2004 (remplaçant la loi 88-1138 du 20 décembre 1988 modifiée dite « loi Huriet-Sérusclat, relative à la protection des personnes se prêtant à des recherches biomédicales (dites loi Huriet)

Management In accordance with currently active legislation, the Teaching hospital of Poitiers has signed an insurance policy with company SHAM (N°131467), so as to cover all possible risks linked to this research study. However, only those persons benefiting from France's Social security regime, or some assimilated regime, will be authorised to participate in this study.

For any further information, yoy may contact and question **professor Denis ORIOT**, any time, at the following Telephones 05-49-45-43-51, mail: simi.recherche@univ-poitiers.fr

After having read this document and been provided with all answers to your questions, and should you agree to participate in this research trial study, we kindly ask you to confirm your decision by signing the consent form enclosed herewith. You will then keep one original copy of each document.

18.2. *Appendix 2 : Form of assent of the participant in the search*

SIM-STRESS:
Effects of stress and simulation in team performance during management of an infant in a state of shock

I undersigned, Mrs, Melle, Mr (Name, First name): ____

Born: ____

Guarantee to have received the letter of information concerning study " ***Effects of the stress and the simulation in the performance of a team during the coverage of an infant in state of shock*** ", of which the Poitiers University Teaching Hospital (CHU de Poitiers) (2 street of Milétrie, 86021 POITIERS-05.49.44.46.65) goes sponsor and of which the investigator coordinator is the **Physician Aïham GHAZALI**(University of Poitiers Teaching Hospital - Service of Urgences-SAMU 86 , 2 street of Milétrie, 86021 POITIERS CEDEX, **05.49.44.37.08)**

I declare to have had a sufficient time of reflection between the information and the assent.

I had the time to read it and to ask all the questions that I wished, and to have been informed (e) by the Physician ………………………………………. Objectives, profits, potential risks and constraints of this study.

I agree freely and voluntarily to participate in the search described above and I am perfectly aware(conscious) that I can remove at any time my assent of participation in this search without having to justify itself, or at all to engage(open) my responsibility. This will pull(entail) in no way consequences on the quality of professional relations with the persons framing(supervising) search.

Besides, I must be also affiliated in, or benefit from a regime of Social Security. I confirm that it is well case.
My assent does not unload(unburden) at all the sponsor and the investigator of their moral and legal responsibilities and I keep(preserve) all my rights guaranteed by the law.

I acquainted that the protocol n°ID-RCB 2013-A00648-37 as well as the letter of information and the assent lit(enlightened) by this study, of which the sponsor is the Poitiers University Teaching Hospital (CHU de Poitiers), obtained the favourable opinion of France's ***third Western Region*** Protection Committee of Human Subjects (CPP Ouest III - "Comité de Protection des Personnes - Ouest III) the XX / XX / 201X, as well as the license in the France's National Agency of Medication and Health Product Security ("ANSM - Agence Nationale de Sécurité du Médicament et des produits de santé"), the XX / XX / 201X.

The sponsor of the study signed a third-party insurance in case of damage with the SHAM company(society) (number of contract : 131467)according to the law.

I declare besides to have been informed to have accepted and, that data registered(recorded) on the occasion of this search can be the object of a treatment computerized by the sponsor or for its account. They will be able to be consulted only by the investigator and his co-workers, by persons appointed by the sponsor and liable in professional secret and by persons appointed by sanitary and judicial authorities.

I noted well that the right of access and rectification, statutory " Data processing and Liberties " (Article 40) exercises at any time with the Physician Aïham GHAZALI and that I arrange a right of rectification of all this information by his(her) intermediary in case of incomplete, inaccurate or ambiguous data.

According to the legislation this study was the object of a statement in the CNIL (Computer National Committee (commission) and Liberties).

This assent is made in three copies, one is put back(handed) to me, another rest in ownership of the investigator and the last one is put back(handed) to the sponsor under sealed envelope**.**

I agree freely and voluntarily to participate in this search in conditions described above. I accept the grip of photos and videos in the unique (only) purpose of the search.

Date: Signature of the participant Signature of the investigator

18.3. Appendix 3: Declaration of Helsinki

Adopted by the 18th WMA General Assembly, Helsinki, Finland, June 1964 and amended by the:
29th WMA General Assembly, Tokyo, Japan, October 1975
35th WMA General Assembly, Venice, Italy, October 1983
41st WMA General Assembly, Hong Kong, September 1989
48th WMA General Assembly, Somerset West, Republic of South Africa, October 1996
52nd WMA General Assembly, Edinburgh, Scotland, October 2000
53rd WMA General Assembly, Washington DC, USA, October 2002 (Note of Clarification added)
55th WMA General Assembly, Tokyo, Japan, October 2004 (Note of Clarification added)
59th WMA General Assembly, Seoul, Republic of Korea, October 2008

A. Introduction

1. The World Medical Association (WMA) has developed the Declaration of Helsinki as a statement of ethical principles for medical research involving human subjects, including research on identifiable human material and data.

   The Declaration is intended to be read as a whole and each of its constituent paragraphs should be applied with consideration of all other relevant paragraphs.
2. Although, the Declaration is addressed primarily to physicians. The WMA encourages others who are involved in medical research involving human subjects to adopt these principles.
3. It is the duty of the physician to promote and safeguard the health, well-being and rights of patients, including those who are involved in medical research. The physician's knowledge and conscience are dedicated to the fulfilment of this duty.
4. The Declaration of Geneva of the WMA binds the physician with the words, “The health of my patient will be my first consideration,” and the International Code of Medical Ethics declares that, “A physician shall act in the patient's best interest when providing medical care.”
5. Medical progress is based on research that ultimately must include studies involving human subjects.
6. In medical research involving human subjects, the well-being of the individual research subjects must take precedence over all interests
7. The primary purpose of medical research involving human subjects is to understand the causes, development and effects of diseases and improve preventive, diagnostic and therapeutic interventions (methods, procedures and treatments). Even the best proven interventions must be evaluated continually through research for their safety, effectiveness, efficiency, accessibility and quality.
8. In medical practice and in medical research, most interventions involve risk and burdens
9. Medical research is subject to ethical standards that promote and ensure respect for all human subjects and protect their health and rights.
10. Physicians should consider the ethical, legal and regulatory norms and standards for research involving human subjects in their own countries as well as applicable international norms and standards. No national or international ethical, legal or regulatory requirement should reduce or eliminate any of the protections for research subjects set forth in this Declaration.

B. Principles for all medical research

11. It is the duty of physicians who are involved in medical research to protect the life, health, dignity, integrity, right to self-determination, privacy, and confidentiality of personal information of research subjects. The responsibility for the protection of research subjects must always rest with the physician or other health care professionals and never with the research subjects, even though they have given consent.

11\. Medical research should be conducted in a manner that minimises possible harm to the environment.

12\. Medical research involving human subjects must conform to generally accepted scientific principles, be based on a thorough knowledge of the scientific literature, other relevant sources of information, and adequate laboratory and, as appropriate, animal experimentation. The welfare of animals used for research must be respected.

13\. Appropriate caution must be exercised in the conduct of medical research that may harm the environment.

14\. The design and performance of each research study invoving human subjects must be clearly described in a research protocol. The protocol should contain a statement of the ethical considerations involved and should indicate how the principles in this declaration have been addressed. The protocol should include information regarding funding, sponsors, institutional affiliations, other potential of interest, incentives for subjects and provisions for treating and/or compensating subjects who are harmed as a consequence of participation in the research study. The protocol should describe arrangements for post-study accss by study subjects to interventions identified as beneficial in the study or access to other appropriate care or benefits

15\. The research must be submitted for consideration, comment, guidance and approval to a research ethics committee before the study begins. This committee must be independent of the researcher, the sponsor and the other undue influence. It must take into consideration the laws and regulations of the country or countries in which the research is to be performed as well as applicable international norms and

standards but these must not be allowed to reduce or eliminate any of the protections for research subjects set forth in this declaration. The committee must have the right to monitor ungoing studies. The researcher must have provide monitoring information to the committee, especially information about any serious adverse events. No change to the proptocol may be made without consideration and approval by the committee.

16. Medical research involving human subjects must be conducted only by individuals with the appropriate scientific training and qualifications. Research on patients or healthy volunteers requires the supervision of a competent and appropriately qualified physician or other health care professional. The responsibility of the protection of the research subjects must always rest with the physician or other health care professional and never the research subjects, even though they have given consent.
17. Medical research invoving a disadvantaged or vulnerable population or community is only justified if the research is responsive to the health needs and priorities of this population or community and if there is a reasonable likelihood that this population or community stands to benefit from the results of the research.
18. Every medical research study involving human subjects must be preeded by careful assessment of predictable risks and brudens to the individuals and communities involved in the research in comparison with foreseeable benefits to them and to other individuals or communities affected by the condition under investigation
19. Every clinical trial must be registered in a publicly accessible database before recrtment of the first subject.
20. Physicians may not participiate in a research study involving human subjects unless they are confident that the risks involved have been adequately assessed and can be satisfactorily manged. Physicians must immediately stop a study when the risks are found to outweigh the potential benefits or when there is conclusive proof of positive and beneficial results.
21. Medical research involving human subjects may only be conducted if the importance of the objective outweighs the inherent risks and burdens to the research subjects.
22. Participation by competent individuals as subjects in medical research must be voluntary. Although it may be appropriate to consult family members or community leaders, no competent individual may be enrolled in a research study unless he or she freely agree.
23. Every precaution must be taken to protect the privacy of research subjects and the confidentiality of their personal information and to minimize the impact of the study on their physicial, mental and social integrity.
24. In medical research involving competent human subjects, each potential subject must be adequately informed of the aims, methods, sources of funding, any possible conflict of interest, institutional affiliations of the researcher, the anticipated benefits and risks of the study and the discomfort it may entail, and any other relevant aspect of the study. The potential subject must be informed of his right to refuse to participate in the study or to withdraw consent to participate at any time without reprisal. Special attention should be given to the specific information needs of individual potential subjects as well as to the methods used to deliver the information. After ensuring that the potential subject has understood the information, the physician or another appropriately qualified individual must then seek the potential subject's freely-given informed consent, preferably in writing. If the consent can not be expressed in writing, the non-written consent must be formally documented and witnessed.
25. For medical research using identifiable human material or data, physicians must normally seek consent for the collection, analysis, storage and / or reuse. There may be situations where consent would be impossible or impractical for such research or would ose a threat to the validity of research. In such situations, the research may be done only after consideration and approval of a research ethics committee.
26. When seeking informed consent for participation in a research study, the physician should be particularly catious if the potential subject is in a dependent relationship with the physician or may consent under duress. In such situations the informed consent should be sought by an appropriately qualified individual who is completely independent of this relationship.
27. For a potential research subject who is incompetent, the physician must seek informed consent from the legally authorized representative. These individuals must not be included in a research study that has no likelihood of benefit for them unless it is intended to romote the health of the population represented by the potential subject, the research cannot instead be performed with comptent persons, and the research cannot instead be performed with competent persons, and the research entails only minimal risk and minimal burden.
28. When a potential research subject who is deemed incompetent is able to give assent to decisions about participation in research, the physician must seek that assent in adition to the consent of the legally authorized representative. The potential subject's dissent should be respected
29. Research involving subjects who are physically or mentally incapable of giving consent, for example unconscious patients, may be done only if the physical or mental condition that prevents giving informed consent is a necessary characteristic of the research population. In such circumstances the physician should seek informed consent from the legally authorized representative. If no such representative is available and if the research can not be delayed, the study may proceed without informed consent provided that the specific reasons for involving subjects with a condition that renders them unable to give

informed consent have ben stated in the research and the study must be approved by a research ethics committee. Consent to remain in the research should be obtained as soon as possible the subject or a legally authorized representative.

30. Authors, editors and publishers all have ethical obligations with regard to the publication of the results of research. Authors have a duty to make publicly available the results of their research on human subjects and are accountable for the completeness and accuracy of their reports. They should adhere to accepted guidelines for ethical reporting. Negative and inconclusive as well as positive results should be published or otherwise made publicly available. Sources of funding, institutional affiliations and conflicts of interest should be declared in the publication. Reports of research not in accordance with the principles of this Declaration should not be accepted for publication.

C. Additional principles for all medical research combined with medical care

31. The physician may combine medical research with medical care only to the extent that the research is justified by its potential preventive, diagnositic or therapeutic value and if the physician has good reason to believe that participation in the research study will not adversely affect the health of the patients who serve as research subjects.
32. The benefits, risks, burdens and effectiveness of a new intervention must be tested against those of the best current proven interention, except in the following circumstances:

- The use of placebo, or no treatment, is acceptable when no current proven intervention exists; or
- Where for compelling and scientifically sound methodological reasons the use of placebo is necessary to determine the efficacy or safety of an intervention and the patients who receive placebo or no treatment will not be subjected to any risks of serious or irreversible harm. Extreme care must be taken to avoid abuse of this option.

33. At the conclusion of the study, patients entered into the study are entitled to be informed about the outcome of the study and to share any benefits that result from it, for example, access to interventions identified as beneficial in the study or to other appropriate care or benefits.
34. The physician must fuly inform the patient which aspects of the care are related to the research. The refusal of a patient to participate in a study or the patient’s decision to withdraw from the study must never interfere with the patient-physician relationship.
35. In the treatment of a patient, where proven interventions do not exist or other known interventions have been ineffective, the physician, after seeking expert advice, with informed consent from the patient or a legally authorized representative, may use an unproven intervention if in the physician's judgement it offers hope of saving life, re-establishing health or alleviating suffering. Where possible, this intervention should be made the object of research, designed to evaluate its safety and efficacy. In all cases, new information should be recorded and, where appropriate, made publicly available.

18.4. Appendix 4: Report form for a severe adverse event

	DECLARATION D'EVENEMENT INDESIRABLE GRAVE (EvIG)	Etude Sim-Stress

EvIG n° |__| : ☐ Déclaration initiale ☐ Déclaration de suivi n°.......

1- PATIENT

Numéro : |__|__|-|__|__|__| Initiales : |__|-|__|

Sexe : M ☐ F ☐ Poids (kg) : |__|__|__| Taille (cm) : |__|__|__|

Date de naissance : |__|__| |__|__| |__|__|__|__|

Date d'inclusion : |__|__| |__|__| |__|__|

Antécédents / facteurs de risque en rapport avec l'évènement *(préciser les dates de début et de fin)* :

....

....

....

2- EVENEMENT INDESIRABLE GRAVE

Critère de gravité de l'évènement

☐ Décès

☐ Mise en jeu du pronostic vital

☐ Hospitalisation ou prolongation d'hospitalisation*

* Date de début : |__|__| |__|__| |__|__|

Date de fin : |__|__| |__|__| |__|__| ou ☐ en cours

☐ Incapacité ou invalidité importante ou durable

☐ Malformation / Anomalie congénitale

☐ Evènement potentiellement grave / Situation médicalement significative

Description de l'évènement (⇒ Joindre les comptes-rendus et les résultats d'examens complémentaires par fax)

....

....

....

....

....

....

....

....

....

....

....

....

→ **Diagnostic retenu** : ....

....

Date de début de l'évènement (1ères manifestations) : |__|__| |__|__| |__|__|

Date d'apparition du critère de gravité : |__|__| |__|__| |__|__|

Mesures prises – Traitements correcteurs :

....

....

....

Evolution de l'évènement :

☐ Résolu sans séquelles

☐ Résolu avec séquelles : ....

} Date de résolution : |__|__| |__|__| |__|__|

☐ En cours de résolution (amélioration)*

☐ Non résolu (état inchangé ou aggravé) *

☐ Evolution inconnue*

} *Une déclaration de suivi devra être complétée

☐ Décès

Patient décédé : Date : |__|__| |__|__| |__|__| Cause : .... En lien avec l'EvIG ? ☐ oui ☐ non

Page 1/2

3- Procédure(s) expérimentale(s)

Description	Date et heure de début	Date et heure de fin	Arrêt prématuré de la procédure ?	Causalité de l'EvIG<br>0 : non lié à la procédure<br>1 : lié à la procédure<br>2 : ne sait pas	Commentaires

4- Traitements concomitants

- Noter tous les médicaments pris par le patient avant la survenue de l'événement.
- Indiquer, si applicable, les modifications de traitement suite à l'évènement.
- Donner votre avis sur le lien de causalité entre l'évènement et chaque médicament.

Médicaments et voies d'administration	Indication	Posologie	Date de début	Action prise<br>0 : poursuite<br>1 : arrêt<br>2: ↘ posologie<br>3: ↗ posologie<br>4 : ne sait pas	Date de l'action	Après l'action, l'EvIG :<br>0 : a disparu<br>1 : s'est amélioré<br>2 : est inchangé/aggravé<br>3 : inconnu	Causalité de l'EvIG<br>0 : non lié au médicament<br>1 : lié au médicament<br>2 : ne sait pas

L'évènement est-il réapparu en cas de réintroduction d'un médicament arrêté ? ☐ oui ☐ non ☐ non applicable
Si oui, de quel(s) médicament(s) s'agit-il ? : ....

Autres traitements non médicamenteux	Indication	Date de début	Action prise<br>0 : poursuite 1 : arrêt<br>2: modification<br>3 : ne sait pas	Date de l'action	Après l'action, l'EvIG<br>0 : a disparu 1 : s'est amélioré<br>2 : est inchangé/aggravé<br>3 : inconnu	Causalité<br>0 : non lié au TT<br>1 : lié au TT<br>2 : ne sait pas

5- Conclusion investigateur

Au total, selon vous, l'évènement indésirable semble plutôt lié :

☐ à la procédure expérimentale (préciser) : ....

☐ à un médicament concomitant (préciser lequel) : ....

☐ à une autre maladie intercurrente : ....

☐ autre : ....

6- Identification investigateur

Nom - Prénom : ....

Date de déclaration : |__|__| |__|__| |__|__| **Signature :**

Déclaration à faxer au promoteur de l'étude FAX : 05.49.44.30.58
CHU de Poitiers
Rue de la Milétrie BP 577, 86021 Poitiers Cedex

Promoteur	
Date de réception promoteur : |__|__| |__|__| |__|__|	Numéro Safety Easy : |__|__|__|__|__| |__|__|__|__|__|__|

Page 2/2

18.5. Appendix 5: Intra-Osseous Performance Assessment Scale IOPAS - (Oriot 2012)

1. **Position of the knee**

Unflexed	**0**
Flexed	**1**

2. **Topical antiseptic**

Not used	**0**
Used on the puncture site	**1**

3. **Gloves**

Absence of gloves or nonsterile gloves	**0**
Sterile gloves	**1**

4. **Local anesthesia**

Absence of anesthesia (in conscious patient)	**0**
Local anesthesia	**1**

5. **Safety guard on the needle**

Absence of guard or guard > 1.5 cm	**0**
Safety guard of 1 cm with thumb-index	**2**

6. **Insertion technique**

Absence of twisting motion	**0**
Mild twisting motion (1 or 2 moves) or non axial insertion	**1**
Axial twisting motion	**3**

7. **Fluid aspiration from marrow cavity with an empty syringe**

Absence of aspiration	**0**
Aspiration of fluid	**2**

8. **Infusion of 0.9% NaCl**

Absence of infusion of 0.9% NaCl	**0**
Infusion of 0.9% NaCl	**2**

9. **Securing the line**

Absence of taping	**0**
Taping the line twice or more on limb and/or abdomen	**1**

10. **Stability of the leg**

Absence of splint	**0**
Splint from knee to ankle	**1**

11. **Location of the puncture site (observed after insertion)**
Landmarks: 1.5 cm below the lower end of the proximal tibial tuberosity, between the anterior and posterior borders of the tibia

Outside the puncture site or mobile needle	**0**
On the puncture site ± 0.5 cm	**3**

12. **Angle of insertion (observed after insertion)**

Oblique insertion	**0**
Perpendicular insertion ± 10°	**2**

TOTAL SCORE:

FIVE KEYS TO SUCCESS
1. Sudden loss of resistance;
2. Needle stands freely and upright without support;
3. Withdrawal of blood or bone marrow;
4. Absence of swelling at the puncture site after infusion of 2 ml of normal saline (NS);
5. Absence of resistance during infusion [illegible]

T1: Preparation time
(IO needle on the skin)
= ...
T2: Placement time
(Tubing attached to the hub of the IO needle)
= ...
T3: Procedure time
(Tubing taped and splint fixed)
= ...

IO access functional?
YES - NO

18.6. Appendix 6: Behavioural Assessment Tool BAT (Anderson 2010). (French translation D. Oriot)

Score	0 (novice)	1	2 (competent)	3	4 (expert)
**1-Knowledge of Environment**	Appears disoriented; is unfamiliar with quipment; fails to ask questions of others in the environment.		Seems somewhat familiar with equipment, asks questions of others in the environment after struggling on their own, appears somewhat familiar with environment.		Appears familiar with surroundings; appears thoroughly familiar with all equipment; readily queries others in the environment when questions arise.
**2-Anticipation and Planning for Potential Problems**	Does not appear prepared for the case; does not inquire of others to gather information; fails to assemble appropriate personnel; fails to react to changing circumstances as case evolves.		May ask 2 to 3 important questions regarding the patient; displays some understanding of possible problems and consequences; may not recognize predictable situations but adapts to changing circumstances; insures presence of necessary personnel and equipment.		Asks pertinent questions indicating an in--depth understanding of potential problems and subsequent consequences of the evolving case; does not appear surprised by predictable situations; insures presence of all necessary personnel and equipment.
**3-Leadership/ Followership**	Fails to clearly identify himself/ herself; stands back, takes a "hands off" approach; appears nervous, "rabled", uncomfortable; fails to inspire confidence.		Identifies self after questioned; enters the situation and takes "hands on" approach when asked to; assumes leadership role but does not clearly coordinate activities of team.		Clearly identifies himself/herself as responsible for patient care; readily enters the situation, takes a "hands on" approach when necessary; coordinates activities of all team members; calmly inspires confidence in leadership.
**4-Communication**	States problem in incorrect or confusing terminology; does not speak clearly; voice is either too soft or too loud; talks down to team members; does not clearly identify to whom he/she is speaking (thin air communications).		Identifies problem but may not communicate clearly to others; tone of voice varies from soft to loud but audible by others in team; clearly identifies those to whom he/she speaks the majority of the time; requests cooperation and listens to others		Specific in problem definition; speaks clearly, succinctly, and in even tones; can easily be heard by the other members; clearly identifies those to whom he/she speaks; listens to others; clarifies ambiguous communication
**5-Distribution of Workload**	Tries to "do it all"; fails to recognize the (potential) contributions of all care providers; does little/ nothing when his/her assistance is required; asks that others exceed their abilities without providing appropriate guidance.		Delegates workload appropriately; assigns specific tasks to care providers but may not utilize those around to the best of their abilities.		Clearly assigns specific tasks to specific care providers; recognizes when other personnel may be over-extended and assists or relieves them as indicated; provides appropriate level of supervision.
**6-Abention Allocation**	Becomes caught up in the details and fails to see the big picture; does not prioritize demands for attention; easily distracted; unable to tune out unimportant input such as background noise.		Recognizes big picture and able to tune out the majority of unnecessary details; adequately prioritizes; avoids fixation errors.		Cognizant of details yet adequately monitors patient's overall condition; does not become distracted; prioritizes well; avoids fixation errors
**7-Use of all Information**	Does not incorporate historical information Into approach to patient; fails to recognize disease states requiring intervention; is slow to recognize emergency situations; ignores part of data in formulating a diagnosis; avoids reaching a conclusion despite a reasonable data base; fails to continually reassess; persists in original course despite indications to change.		Incorporates pertinent historical information Into approach to patient; interprets most physical findings accurately; assess need for intervention; recognizes changes in patient condition; re-assesses patient status as needed.		Incorporates pertinent historical information into approach to patient; interprets findings accurately; repeats examination when findings are equivocal; correctly assesses need for intervention; readily recognizes changes in patient condition and keeps assessment of patient and management plan current; persistent in seeking information.
**8- Utilization of all Available Resources**	Fails to recognize professional skills of others; does not recognize potential alternatives when presented with equipment or personnel failures.		Utilizes expertise of other team members appropriately most of the time; may struggle with equipment or personnel failures but eventually problem solves for other solutions.		Solicits and incorporates expertise of other caretakers appropriately; reacts to equipment or personnel failures by identifying alternative solutions.
**9- Recognition of Limitations/Calls For Help Early Enough**	Fails to recognize own limitations; does not request assistance when needed; recognizes own limitations but attempts to exceed these limitations, thereby placing patient at risk; calls for help when not indicated.		Recognizes own limitations in knowledge and skill but delays calling for help.		Recognizes when at his/her limits in medical knowledge and technical skill and readily asks for assistance; recognizes situations where additional help will be required and requests such assistance early.
**10- Professionalism**	Engages in unnecessary conversation; makes inappropriate comments; uses profanity; shows little concern for patient's comfort; approach to family members is demeaning, abrupt, clumsy or otherwise inappropriate; is unable to give or take advice gracefully; becomes defensive.		Keeps unnecessary conversation to a minimum; language and approach are professional most of the time; regards family with a caring attitude; recognizes and responds to the majority of nonverbal and verbal cues; supervises and teaches in a non threatening manner.		Maintains composure; does not engage in unnecessary conversation; demonstrates a caring attitude toward patients and families; recognizes and responds to nonverbal cues; encourages input from other team members; supervises and teaches effectively; non-judgmental; non-defensive.
**TOTAL over 40**					
**TOTAL over 100**					

18.7. Appendix 7: Clinical Teamwork Scale CTS (Guise 2008). (French translation A. Ghazali).

CTS - Clinical Teamwork Scale™ (Global)

Please note: **Not relevant**- The task was not applicable to the scenario.

**Overall**	Not Relevant	Unacceptable	Poor			Average			Good			Perfect
**1. How would you rate teamwork during this delivery/emergency?**	□	0	1	2	3	4	5	6	7	8	9	10

**Communication**	Not Relevant	Unacceptable	Poor			Average			Good			Perfect
**Overall Communication Rating:**	□	0	1	2	3	4	5	6	7	8	9	10
**1. Orient new members (SBAR)**	□	0	1	2	3	4	5	6	7	8	9	10
**2. Transparent thinking**	□	0	1	2	3	4	5	6	7	8	9	10
**3. Directed communication**	□	0	1	2	3	4	5	6	7	8	9	10
**4. Closed loop communication**	□	0	1	2	3	4	5	6	7	8	9	10

**Situational Awareness**	Not Relevant	Unacceptable	Poor			Average			Good			Perfect
**Overall Situational Awareness Rating:**	□	0	1	2	3	4	5	6	7	8	9	10
**1. Resource allocation**	□	0	1	2	3	4	5	6	7	8	9	10
**2. Target fixation**	□ Yes	□ No										

**Decision Making**	Not Relevant	Unacceptable	Poor			Average			Good			Perfect
**Overall Decision Making Rating:**	□	0	1	2	3	4	5	6	7	8	9	10
**1. Prioritize**	□	0	1	2	3	4	5	6	7	8	9	10

**Role Responsibility**	Not Relevant	Unacceptable	Poor			Average			Good			Perfect
**Overall Role Responsibility (Leader/Helper) Rating:**	□	0	1	2	3	4	5	6	7	8	9	10
**1. Role clarity**	□	0	1	2	3	4	5	6	7	8	9	10
**2. Perform as a leader/helper**	□	0	1	2	3	4	5	6	7	8	9	10

**Other**	Not Relevant	Unacceptable	Poor			Average			Good			Perfect
**1. Patient friendly**	□	0	1	2	3	4	5	6	7	8	9	10

18.8. Appendix 8 : State-Trait Anxiety Inventory STAI (Spielberger 1983) (French translation D. Oriot)

Instructions: Read each statement and select the appropriate response to indicate how you feel right now, that is, at this very moment. There are no right or wrong answers. Do not spend too much time on any one statement but give the answer which seems to describe your present feelings best.

		At all 1	A little 2	Averagely 3	A lot(many) 4
1	I feel calm				
2	I feel secure				
3	I feel tense				
4	I feel strained				
5	I feel at ease				
6	I feel upset				
7	I am presently worrying over possible misfortunes				
8	I feel satisfied				
9	I feel frightened				
10	I feel uncomfortable				
11	I feel self confident				
12	I feel nervous				
13	I feel jittery				
14	I feel indecisive				
15	I feel relaxed				
16	I feel content				
17	I am worried				
18	I feel confused				
19	I feel steady				
20	I feel pleasant				
TOTAL SCORE on 80					
TOTAL SCORE on 100					

18.9. Appendix 9: Impact of Event Scale-Revised IES-R (Brunet 2003)

INSTRUCTIONS:

Below is a list of difficulties people sometimes have after stressful life events. Please read each item, and then indicate how distressing each difficulty has been for you DURING THE PAST SEVEN DAYS with respect to ______________________________, which occurred on _______________. How much were you distressed or bothered by these difficulties?

Item Response Anchors are 0 = Not at all; 1 = A little bit; 2 = Moderately; 3 = Quite a bit; 4 = Extremely.

The Intrusion subscale is the MEAN item response of items 1, 2, 3, 6, 9, 14, 16, 20. Thus, scores can range from 0 through 4.
The Avoidance subscale is the MEAN item response of items 5, 7, 8, 11, 12, 13, 17, 22. Thus, scores can range from 0 through 4.
The Hyperarousal subscale is the MEAN item response of items 4, 10, 15, 18, 19, 21. Thus, scores can range from 0 through 4.

1. Any reminder brought back feelings about it.
2. I had trouble staying asleep.
3. Other things kept making me think about it.
4. I felt irritable and angry.
5. I avoided letting myself get upset when I thought about it or was reminded of it.
6. I thought about it when I didn't mean to.
7. I felt as if it hadn't happened or wasn't real.
8. I stayed away from reminders of it.
9. Pictures about it popped into my mind.
10. I was jumpy and easily startled.
11. I tried not to think about it.
12. I was aware that I still had a lot of feelings about it, but I didn't deal with them.
13. My feelings about it were kind of numb.
14. I found myself acting or feeling like I was back at that time.
15. I had trouble falling asleep.
16. I had waves of strong feelings about it.
17. I tried to remove it from my memory.
18. I had trouble concentrating.
19. Reminders of it caused me to have physical reactions, such as sweating, trouble breathing, nausea, or a pounding heart.
20. I had dreams about it.
21. I felt watchful and on-guard.
22. I tried not to talk about it.

Total IES-R score:_____________

18.10. Appendix 10 : Post-traumatic Check List Scale PCLS (Weathers 1993)

Patient's Name: ____ Instructions: Below is a list of problems and complaints that people sometimes have in response to stressful life experiences. Please read each one carefully, put an "X" in the box to indicate how much you have been bothered by that problem in the past month. The event you experienced was ________________________ on ______________(date)

	1	2	3	4	5
	**Not at all**	**A little bit**	**Moderately**	**Quite a bit**	**Extremely**
1. Repeated, disturbing memories, thoughts, or images of a stressful experience from the past?					
2. Repeated, disturbing dreams of a stressful experience from the past?					
3. Suddenly acting or feeling as if a stressful experience were happening again (as if you were reliving it)?					
4. Feeling very upset when something reminded you of a stressful experience from the past?					
5. Having physical reactions (e.g., heart pounding, trouble breathing, or sweating) when something reminded you of a stressful experience from the past?					
6. Avoid thinking about or talking about a stressful experience from the past or avoid having feelings related to it?					
7. Avoid activities or situations because they remind you of a stressful experience from the past?					
8. Trouble remembering important parts of a stressful experience from the past?					
9. Loss of interest in things that you used to enjoy?					
10. Feeling distant or cut off from other people?					
11. Feeling emotionally numb or being unable to have loving feelings for those close to you?					
12. Feeling as if your future will somehow be cut short?					
13. Trouble falling or staying asleep?					
14. Feeling irritable or having angry outbursts?					
15. Having difficulty concentrating?					
16. Being "super alert" or watchful on guard?					
17. Feeling jumpy or easily startled?					
**TOTAL SCORE on 85**					
**TOTAL SCORE on 100**					
